# Supplementary material for: Genome-wide identification, characterization and gene expression of BES1 transcription factor family in grapevine (Vitis vinifera L.)
Source: Sci Rep. 2023 Jan 5;13:240. doi: 10.1038/s41598-022-24407-y (PMC9816167; doi:10.1038/s41598-022-24407-y)
Supplement: Supplementary file 3 — Supplementary Information. [file 41598_2022_24407_MOESM3_ESM.zip › Vvi_Atr/Vitis_vinifera.PN40024.v4.dna_sm.toplevel.fa.vs.Amborella_trichopoda.AMTR1.0.dna_sm.toplevel.fa.html/Vvi-7.html]

|  |  |  |  |  |  |  |  |  |  |  |  |  |  |
| --- | --- | --- | --- | --- | --- | --- | --- | --- | --- | --- | --- | --- | --- |
| Duplication depth | Reference chromosome | Collinear blocks | | | | | | | | | | | |
| 0 | Vvi-Vitvi07g00002\_t001 |  |  |  |  |  |  |  |  |  |  |  |  |
| 0 | Vvi-Vitvi07g04000\_t001 |  |  |  |  |  |  |  |  |  |  |  |  |
| 0 | Vvi-Vitvi07g04001\_t001 |  |  |  |  |  |  |  |  |  |  |  |  |
| 0 | Vvi-Vitvi07g04002\_t001 |  |  |  |  |  |  |  |  |  |  |  |  |
| 0 | Vvi-Vitvi07g00011\_t001 |  |  |  |  |  |  |  |  |  |  |  |  |
| 0 | Vvi-Vitvi07g00012\_t001 |  |  |  |  |  |  |  |  |  |  |  |  |
| 1 | Vvi-Vitvi07g00013\_t001 |  | Atr-ERN01383 |  |  |  |  |  |  |  |  |  |  |
| 1 | Vvi-Vitvi07g00015\_t001 |  | Atr-ERN01385 |  |  |  |  |  |  |  |  |  |  |
| 1 | Vvi-Vitvi07g00016\_t001 |  | Atr-ERN01387 |  |  |  |  |  |  |  |  |  |  |
| 1 | Vvi-Vitvi07g04003\_t001 |  | \| \| |  |  |  |  |  |  |  |  |  |  |
| 1 | Vvi-Vitvi07g00017\_t001 |  | \| \| |  |  |  |  |  |  |  |  |  |  |
| 1 | Vvi-Vitvi07g00018\_t001 |  | Atr-ERN01403 |  |  |  |  |  |  |  |  |  |  |
| 1 | Vvi-Vitvi07g00020\_t001 |  | Atr-ERN01404 |  |  |  |  |  |  |  |  |  |  |
| 1 | Vvi-Vitvi07g00021\_t001 |  | \| \| |  |  |  |  |  |  |  |  |  |  |
| 1 | Vvi-Vitvi07g04004\_t001 |  | \| \| |  |  |  |  |  |  |  |  |  |  |
| 1 | Vvi-Vitvi07g00024\_t001 |  | Atr-ERN01411 |  |  |  |  |  |  |  |  |  |  |
| 1 | Vvi-Vitvi07g00025\_t001 |  | \| \| |  |  |  |  |  |  |  |  |  |  |
| 1 | Vvi-Vitvi07g02066\_t001 |  | Atr-ERN01413 |  |  |  |  |  |  |  |  |  |  |
| 1 | Vvi-Vitvi07g00026\_t001 |  | Atr-ERN01414 |  |  |  |  |  |  |  |  |  |  |
| 1 | Vvi-Vitvi07g00027\_t001 |  | Atr-ERN01415 |  |  |  |  |  |  |  |  |  |  |
| 1 | Vvi-Vitvi07g00029\_t001 |  | Atr-ERN01417 |  |  |  |  |  |  |  |  |  |  |
| 1 | Vvi-Vitvi07g00030\_t001 |  | Atr-ERN01421 |  |  |  |  |  |  |  |  |  |  |
| 1 | Vvi-Vitvi07g00031\_t001 |  | Atr-ERN01426 |  |  |  |  |  |  |  |  |  |  |
| 1 | Vvi-Vitvi07g00032\_t001 |  | Atr-ERN01427 |  |  |  |  |  |  |  |  |  |  |
| 1 | Vvi-Vitvi07g00034\_t001 |  | Atr-ERN01428 |  |  |  |  |  |  |  |  |  |  |
| 1 | Vvi-Vitvi07g00035\_t001 |  | Atr-ERN01429 |  |  |  |  |  |  |  |  |  |  |
| 1 | Vvi-Vitvi07g04005\_t001 |  | \| \| |  |  |  |  |  |  |  |  |  |  |
| 1 | Vvi-Vitvi07g04006\_t001 |  | \| \| |  |  |  |  |  |  |  |  |  |  |
| 1 | Vvi-Vitvi07g04007\_t001 |  | \| \| |  |  |  |  |  |  |  |  |  |  |
| 1 | Vvi-Vitvi07g04008\_t001 |  | \| \| |  |  |  |  |  |  |  |  |  |  |
| 1 | Vvi-Vitvi07g04009\_t001 |  | \| \| |  |  |  |  |  |  |  |  |  |  |
| 1 | Vvi-Vitvi07g04010\_t001 |  | Atr-ERN01432 |  |  |  |  |  |  |  |  |  |  |
| 1 | Vvi-Vitvi07g04011\_t001 |  | \| \| |  |  |  |  |  |  |  |  |  |  |
| 1 | Vvi-Vitvi07g02092\_t001 |  | \| \| |  |  |  |  |  |  |  |  |  |  |
| 1 | Vvi-Vitvi07g04012\_t001 |  | \| \| |  |  |  |  |  |  |  |  |  |  |
| 1 | Vvi-Vitvi07g02094\_t001 |  | \| \| |  |  |  |  |  |  |  |  |  |  |
| 1 | Vvi-Vitvi07g04013\_t001 |  | \| \| |  |  |  |  |  |  |  |  |  |  |
| 1 | Vvi-Vitvi07g00036\_t003 |  | \| \| |  |  |  |  |  |  |  |  |  |  |
| 1 | Vvi-Vitvi07g00037\_t002 |  | \| \| |  |  |  |  |  |  |  |  |  |  |
| 1 | Vvi-Vitvi07g04014\_t001 |  | \| \| |  |  |  |  |  |  |  |  |  |  |
| 1 | Vvi-Vitvi07g00038\_t001 |  | \| \| |  |  |  |  |  |  |  |  |  |  |
| 1 | Vvi-Vitvi07g00039\_t001 |  | \| \| |  |  |  |  |  |  |  |  |  |  |
| 1 | Vvi-Vitvi07g04015\_t001 |  | \| \| |  |  |  |  |  |  |  |  |  |  |
| 1 | Vvi-Vitvi07g00040\_t001 |  | Atr-ERN01433 |  |  |  |  |  |  |  |  |  |  |
| 1 | Vvi-Vitvi07g00041\_t001 |  | Atr-ERN01434 |  |  |  |  |  |  |  |  |  |  |
| 1 | Vvi-Vitvi07g00042\_t001 |  | Atr-ERN01436 |  |  |  |  |  |  |  |  |  |  |
| 1 | Vvi-Vitvi07g00044\_t001 |  | Atr-ERN01443 |  |  |  |  |  |  |  |  |  |  |
| 1 | Vvi-Vitvi07g00045\_t001 |  | Atr-ERN01447 |  |  |  |  |  |  |  |  |  |  |
| 1 | Vvi-Vitvi07g00046\_t001 |  | Atr-ERN01450 |  |  |  |  |  |  |  |  |  |  |
| 1 | Vvi-Vitvi07g00047\_t001 |  | Atr-ERN01455 |  |  |  |  |  |  |  |  |  |  |
| 1 | Vvi-Vitvi07g00048\_t001 |  | \| \| |  |  |  |  |  |  |  |  |  |  |
| 1 | Vvi-Vitvi07g00049\_t001 |  | Atr-ERN01458 |  |  |  |  |  |  |  |  |  |  |
| 1 | Vvi-Vitvi07g00050\_t001 |  | Atr-ERN01460 |  |  |  |  |  |  |  |  |  |  |
| 1 | Vvi-Vitvi07g00052\_t001 |  | \| \| |  |  |  |  |  |  |  |  |  |  |
| 1 | Vvi-Vitvi07g00053\_t001 |  | \| \| |  |  |  |  |  |  |  |  |  |  |
| 1 | Vvi-Vitvi07g00054\_t001 |  | Atr-ERN01464 |  |  |  |  |  |  |  |  |  |  |
| 1 | Vvi-Vitvi07g00055\_t001 |  | \| \| |  |  |  |  |  |  |  |  |  |  |
| 1 | Vvi-Vitvi07g04016\_t001 |  | \| \| |  |  |  |  |  |  |  |  |  |  |
| 1 | Vvi-Vitvi07g04017\_t001 |  | \| \| |  |  |  |  |  |  |  |  |  |  |
| 1 | Vvi-Vitvi07g04018\_t001 |  | \| \| |  |  |  |  |  |  |  |  |  |  |
| 1 | Vvi-Vitvi07g00057\_t001 |  | \| \| |  |  |  |  |  |  |  |  |  |  |
| 1 | Vvi-Vitvi07g02095\_t001 |  | \| \| |  |  |  |  |  |  |  |  |  |  |
| 1 | Vvi-Vitvi07g02096\_t001 |  | \| \| |  |  |  |  |  |  |  |  |  |  |
| 1 | Vvi-Vitvi07g00059\_t001 |  | \| \| |  |  |  |  |  |  |  |  |  |  |
| 1 | Vvi-Vitvi07g00060\_t001 |  | Atr-ERN01470 |  |  |  |  |  |  |  |  |  |  |
| 1 | Vvi-Vitvi07g00061\_t002 |  | Atr-ERN01471 |  |  |  |  |  |  |  |  |  |  |
| 1 | Vvi-Vitvi07g00062\_t001 |  | Atr-ERN01472 |  |  |  |  |  |  |  |  |  |  |
| 1 | Vvi-Vitvi07g02097\_t001 |  | \| \| |  |  |  |  |  |  |  |  |  |  |
| 1 | Vvi-Vitvi07g00063\_t001 |  | \| \| |  |  |  |  |  |  |  |  |  |  |
| 1 | Vvi-Vitvi07g00064\_t001 |  | Atr-ERN01473 |  |  |  |  |  |  |  |  |  |  |
| 1 | Vvi-Vitvi07g00065\_t001 |  | Atr-ERN01479 |  |  |  |  |  |  |  |  |  |  |
| 1 | Vvi-Vitvi07g00066\_t001 |  | Atr-ERN01483 |  |  |  |  |  |  |  |  |  |  |
| 1 | Vvi-Vitvi07g00067\_t001 |  | \| \| |  |  |  |  |  |  |  |  |  |  |
| 1 | Vvi-Vitvi07g00068\_t001 |  | \| \| |  |  |  |  |  |  |  |  |  |  |
| 1 | Vvi-Vitvi07g00069\_t001 |  | \| \| |  |  |  |  |  |  |  |  |  |  |
| 1 | Vvi-Vitvi07g00071\_t001 |  | \| \| |  |  |  |  |  |  |  |  |  |  |
| 1 | Vvi-Vitvi07g00072\_t001 |  | \| \| |  |  |  |  |  |  |  |  |  |  |
| 1 | Vvi-Vitvi07g00073\_t002 |  | Atr-ERN01495 |  |  |  |  |  |  |  |  |  |  |
| 1 | Vvi-Vitvi07g00074\_t001 |  | Atr-ERN01504 |  |  |  |  |  |  |  |  |  |  |
| 1 | Vvi-Vitvi07g00075\_t001 |  | Atr-ERN01510 |  |  |  |  |  |  |  |  |  |  |
| 1 | Vvi-Vitvi07g00077\_t001 |  | Atr-ERN01511 |  |  |  |  |  |  |  |  |  |  |
| 1 | Vvi-Vitvi07g04019\_t001 |  | \| \| |  |  |  |  |  |  |  |  |  |  |
| 1 | Vvi-Vitvi07g00078\_t001 |  | Atr-ERN01513 |  |  |  |  |  |  |  |  |  |  |
| 1 | Vvi-Vitvi07g00079\_t001 |  | Atr-ERN01515 |  |  |  |  |  |  |  |  |  |  |
| 1 | Vvi-Vitvi07g00080\_t001 |  | \| \| |  |  |  |  |  |  |  |  |  |  |
| 1 | Vvi-Vitvi07g00081\_t001 |  | Atr-ERN01519 |  |  |  |  |  |  |  |  |  |  |
| 1 | Vvi-Vitvi07g00082\_t001 |  | Atr-ERN01521 |  |  |  |  |  |  |  |  |  |  |
| 1 | Vvi-Vitvi07g02102\_t001 |  | Atr-ERN01525 |  |  |  |  |  |  |  |  |  |  |
| 1 | Vvi-Vitvi07g02103\_t001 |  | \| \| |  |  |  |  |  |  |  |  |  |  |
| 1 | Vvi-Vitvi07g04020\_t001 |  | \| \| |  |  |  |  |  |  |  |  |  |  |
| 1 | Vvi-Vitvi07g04021\_t001 |  | \| \| |  |  |  |  |  |  |  |  |  |  |
| 1 | Vvi-Vitvi07g04022\_t001 |  | \| \| |  |  |  |  |  |  |  |  |  |  |
| 1 | Vvi-Vitvi07g02106\_t001 |  | \| \| |  |  |  |  |  |  |  |  |  |  |
| 1 | Vvi-Vitvi07g02107\_t001 |  | \| \| |  |  |  |  |  |  |  |  |  |  |
| 1 | Vvi-Vitvi07g02108\_t001 |  | \| \| |  |  |  |  |  |  |  |  |  |  |
| 1 | Vvi-Vitvi07g00085\_t001 |  | \| \| |  |  |  |  |  |  |  |  |  |  |
| 1 | Vvi-Vitvi07g04023\_t001 |  | \| \| |  |  |  |  |  |  |  |  |  |  |
| 1 | Vvi-Vitvi07g04024\_t001 |  | \| \| |  |  |  |  |  |  |  |  |  |  |
| 1 | Vvi-Vitvi07g02110\_t001 |  | \| \| |  |  |  |  |  |  |  |  |  |  |
| 1 | Vvi-Vitvi07g04025\_t001 |  | \| \| |  |  |  |  |  |  |  |  |  |  |
| 1 | Vvi-Vitvi07g02111\_t001 |  | \| \| |  |  |  |  |  |  |  |  |  |  |
| 1 | Vvi-Vitvi07g04026\_t001 |  | \| \| |  |  |  |  |  |  |  |  |  |  |
| 1 | Vvi-Vitvi07g02114\_t001 |  | Atr-ERN01527 |  |  |  |  |  |  |  |  |  |  |
| 1 | Vvi-Vitvi07g02115\_t001 |  | \| \| |  |  |  |  |  |  |  |  |  |  |
| 1 | Vvi-Vitvi07g04027\_t001 |  | \| \| |  |  |  |  |  |  |  |  |  |  |
| 1 | Vvi-Vitvi07g04028\_t001 |  | \| \| |  |  |  |  |  |  |  |  |  |  |
| 1 | Vvi-Vitvi07g02119\_t001 |  | \| \| |  |  |  |  |  |  |  |  |  |  |
| 1 | Vvi-Vitvi07g02120\_t001 |  | \| \| |  |  |  |  |  |  |  |  |  |  |
| 1 | Vvi-Vitvi07g02121\_t001 |  | \| \| |  |  |  |  |  |  |  |  |  |  |
| 1 | Vvi-Vitvi07g02122\_t001 |  | \| \| |  |  |  |  |  |  |  |  |  |  |
| 1 | Vvi-Vitvi07g02123\_t001 |  | \| \| |  |  |  |  |  |  |  |  |  |  |
| 1 | Vvi-Vitvi07g00087\_t001 |  | Atr-ERN01529 |  |  |  |  |  |  |  |  |  |  |
| 1 | Vvi-Vitvi07g00088\_t001 |  | Atr-ERN01530 |  |  |  |  |  |  |  |  |  |  |
| 1 | Vvi-Vitvi07g04029\_t001 |  | Atr-ERN01545 |  |  |  |  |  |  |  |  |  |  |
| 1 | Vvi-Vitvi07g00090\_t001 |  | Atr-ERN01548 |  |  |  |  |  |  |  |  |  |  |
| 1 | Vvi-Vitvi07g04030\_t001 |  | \| \| |  |  |  |  |  |  |  |  |  |  |
| 1 | Vvi-Vitvi07g04031\_t001 |  | \| \| |  |  |  |  |  |  |  |  |  |  |
| 1 | Vvi-Vitvi07g00091\_t001 |  | Atr-ERN01554 |  |  |  |  |  |  |  |  |  |  |
| 1 | Vvi-Vitvi07g00094\_t001 |  | \| \| |  |  |  |  |  |  |  |  |  |  |
| 1 | Vvi-Vitvi07g00095\_t001 |  | \| \| |  |  |  |  |  |  |  |  |  |  |
| 1 | Vvi-Vitvi07g00096\_t001 |  | \| \| |  |  |  |  |  |  |  |  |  |  |
| 1 | Vvi-Vitvi07g00097\_t002 |  | Atr-ERN01560 |  |  |  |  |  |  |  |  |  |  |
| 1 | Vvi-Vitvi07g00099\_t001 |  | Atr-ERN01566 |  |  |  |  |  |  |  |  |  |  |
| 1 | Vvi-Vitvi07g04032\_t001 |  | Atr-ERN01567 |  |  |  |  |  |  |  |  |  |  |
| 1 | Vvi-Vitvi07g00102\_t001 |  | Atr-ERN01569 |  |  |  |  |  |  |  |  |  |  |
| 1 | Vvi-Vitvi07g00103\_t001 |  | Atr-ERN01572 |  |  |  |  |  |  |  |  |  |  |
| 1 | Vvi-Vitvi07g00104\_t001 |  | \| \| |  |  |  |  |  |  |  |  |  |  |
| 1 | Vvi-Vitvi07g00105\_t001 |  | Atr-ERN01575 |  |  |  |  |  |  |  |  |  |  |
| 1 | Vvi-Vitvi07g04033\_t001 |  | \| \| |  |  |  |  |  |  |  |  |  |  |
| 1 | Vvi-Vitvi07g04034\_t001 |  | \| \| |  |  |  |  |  |  |  |  |  |  |
| 1 | Vvi-Vitvi07g02126\_t001 |  | \| \| |  |  |  |  |  |  |  |  |  |  |
| 1 | Vvi-Vitvi07g04035\_t001 |  | \| \| |  |  |  |  |  |  |  |  |  |  |
| 1 | Vvi-Vitvi07g04036\_t001 |  | \| \| |  |  |  |  |  |  |  |  |  |  |
| 1 | Vvi-Vitvi07g00106\_t001 |  | Atr-ERN01578 |  |  |  |  |  |  |  |  |  |  |
| 1 | Vvi-Vitvi07g00107\_t001 |  | Atr-ERN01579 |  |  |  |  |  |  |  |  |  |  |
| 1 | Vvi-Vitvi07g02127\_t002 |  | \| \| |  |  |  |  |  |  |  |  |  |  |
| 1 | Vvi-Vitvi07g04037\_t001 |  | \| \| |  |  |  |  |  |  |  |  |  |  |
| 1 | Vvi-Vitvi07g00109\_t001 |  | Atr-ERN01582 |  |  |  |  |  |  |  |  |  |  |
| 1 | Vvi-Vitvi07g00110\_t001 |  | Atr-ERN01587 |  |  |  |  |  |  |  |  |  |  |
| 1 | Vvi-Vitvi07g02129\_t001 |  | \| \| |  |  |  |  |  |  |  |  |  |  |
| 1 | Vvi-Vitvi07g02130\_t001 |  | \| \| |  |  |  |  |  |  |  |  |  |  |
| 1 | Vvi-Vitvi07g02131\_t001 |  | Atr-ERN01588 |  |  |  |  |  |  |  |  |  |  |
| 1 | Vvi-Vitvi07g02132\_t001 |  | \| \| |  |  |  |  |  |  |  |  |  |  |
| 1 | Vvi-Vitvi07g04038\_t001 |  | \| \| |  |  |  |  |  |  |  |  |  |  |
| 1 | Vvi-Vitvi07g00112\_t001 |  | Atr-ERN01590 |  |  |  |  |  |  |  |  |  |  |
| 1 | Vvi-Vitvi07g00113\_t001 |  | Atr-ERN01596 |  |  |  |  |  |  |  |  |  |  |
| 0 | Vvi-Vitvi07g00114\_t001 |  |  |  |  |  |  |  |  |  |  |  |  |
| 0 | Vvi-Vitvi07g00115\_t001 |  |  |  |  |  |  |  |  |  |  |  |  |
| 0 | Vvi-Vitvi07g04039\_t001 |  |  |  |  |  |  |  |  |  |  |  |  |
| 0 | Vvi-Vitvi07g00116\_t001 |  |  |  |  |  |  |  |  |  |  |  |  |
| 0 | Vvi-Vitvi07g00117\_t001 |  |  |  |  |  |  |  |  |  |  |  |  |
| 0 | Vvi-Vitvi07g04040\_t001 |  |  |  |  |  |  |  |  |  |  |  |  |
| 0 | Vvi-Vitvi07g04041\_t001 |  |  |  |  |  |  |  |  |  |  |  |  |
| 0 | Vvi-Vitvi07g00120\_t001 |  |  |  |  |  |  |  |  |  |  |  |  |
| 0 | Vvi-Vitvi07g00121\_t001 |  |  |  |  |  |  |  |  |  |  |  |  |
| 1 | Vvi-Vitvi07g00122\_t001 |  | Atr-ERN00801 |  |  |  |  |  |  |  |  |  |  |
| 1 | Vvi-Vitvi07g00123\_t001 |  | Atr-ERN00802 |  |  |  |  |  |  |  |  |  |  |
| 1 | Vvi-Vitvi07g00124\_t001 |  | Atr-ERN00805 |  |  |  |  |  |  |  |  |  |  |
| 1 | Vvi-Vitvi07g02133\_t001 |  | Atr-ERN00806 |  |  |  |  |  |  |  |  |  |  |
| 1 | Vvi-Vitvi07g02134\_t001 |  | Atr-ERN00813 |  |  |  |  |  |  |  |  |  |  |
| 1 | Vvi-Vitvi07g00125\_t001 |  | Atr-ERN00816 |  |  |  |  |  |  |  |  |  |  |
| 1 | Vvi-Vitvi07g02135\_t001 |  | \| \| |  |  |  |  |  |  |  |  |  |  |
| 1 | Vvi-Vitvi07g00126\_t001 |  | Atr-ERN00817 |  |  |  |  |  |  |  |  |  |  |
| 1 | Vvi-Vitvi07g00127\_t001 |  | Atr-ERN00818 |  |  |  |  |  |  |  |  |  |  |
| 1 | Vvi-Vitvi07g00128\_t001 |  | Atr-ERN00819 |  |  |  |  |  |  |  |  |  |  |
| 1 | Vvi-Vitvi07g00129\_t001 |  | \| \| |  |  |  |  |  |  |  |  |  |  |
| 1 | Vvi-Vitvi07g00130\_t001 |  | \| \| |  |  |  |  |  |  |  |  |  |  |
| 1 | Vvi-Vitvi07g04042\_t001 |  | \| \| |  |  |  |  |  |  |  |  |  |  |
| 1 | Vvi-Vitvi07g00131\_t001 |  | Atr-ERN00832 |  |  |  |  |  |  |  |  |  |  |
| 1 | Vvi-Vitvi07g04043\_t001 |  | \| \| |  |  |  |  |  |  |  |  |  |  |
| 1 | Vvi-Vitvi07g00132\_t001 |  | Atr-ERN00834 |  |  |  |  |  |  |  |  |  |  |
| 1 | Vvi-Vitvi07g02136\_t001 |  | Atr-ERN00840 |  |  |  |  |  |  |  |  |  |  |
| 0 | Vvi-Vitvi07g02137\_t001 |  |  |  |  |  |  |  |  |  |  |  |  |
| 0 | Vvi-Vitvi07g00134\_t001 |  |  |  |  |  |  |  |  |  |  |  |  |
| 0 | Vvi-Vitvi07g00135\_t001 |  |  |  |  |  |  |  |  |  |  |  |  |
| 1 | Vvi-Vitvi07g00136\_t001 |  | Atr-ERN12300 |  |  |  |  |  |  |  |  |  |  |
| 1 | Vvi-Vitvi07g00137\_t001 |  | Atr-ERN12302 |  |  |  |  |  |  |  |  |  |  |
| 1 | Vvi-Vitvi07g00138\_t001.1.6037826f |  | \| \| |  |  |  |  |  |  |  |  |  |  |
| 1 | Vvi-Vitvi07g00139\_t001 |  | Atr-ERN12312 |  |  |  |  |  |  |  |  |  |  |
| 1 | Vvi-Vitvi07g00140\_t001 |  | \| \| |  |  |  |  |  |  |  |  |  |  |
| 1 | Vvi-Vitvi07g00141\_t001 |  | Atr-ERN12326 |  |  |  |  |  |  |  |  |  |  |
| 1 | Vvi-Vitvi07g00142\_t001 |  | \| \| |  |  |  |  |  |  |  |  |  |  |
| 1 | Vvi-Vitvi07g04044\_t001 |  | \| \| |  |  |  |  |  |  |  |  |  |  |
| 1 | Vvi-Vitvi07g00144\_t001 |  | \| \| |  |  |  |  |  |  |  |  |  |  |
| 1 | Vvi-Vitvi07g02138\_t001 |  | \| \| |  |  |  |  |  |  |  |  |  |  |
| 1 | Vvi-Vitvi07g00145\_t002 |  | \| \| |  |  |  |  |  |  |  |  |  |  |
| 1 | Vvi-Vitvi07g00146\_t001 |  | Atr-ERN12344 |  |  |  |  |  |  |  |  |  |  |
| 1 | Vvi-Vitvi07g00147\_t001 |  | \| \| |  |  |  |  |  |  |  |  |  |  |
| 1 | Vvi-Vitvi07g02140\_t002 |  | Atr-ERN12353 |  |  |  |  |  |  |  |  |  |  |
| 1 | Vvi-Vitvi07g02141\_t001 |  | \| \| |  |  |  |  |  |  |  |  |  |  |
| 1 | Vvi-Vitvi07g00149\_t001 |  | \| \| |  |  |  |  |  |  |  |  |  |  |
| 1 | Vvi-Vitvi07g00150\_t002 |  | Atr-ERN12354 |  |  |  |  |  |  |  |  |  |  |
| 1 | Vvi-Vitvi07g00151\_t001 |  | Atr-ERN12355 |  |  |  |  |  |  |  |  |  |  |
| 1 | Vvi-Vitvi07g00152\_t001 |  | Atr-ERN12357 |  |  |  |  |  |  |  |  |  |  |
| 1 | Vvi-Vitvi07g00154\_t001 |  | Atr-ERN12361 |  |  |  |  |  |  |  |  |  |  |
| 1 | Vvi-Vitvi07g00155\_t001 |  | \| \| |  |  |  |  |  |  |  |  |  |  |
| 1 | Vvi-Vitvi07g00157\_t001 |  | \| \| |  |  |  |  |  |  |  |  |  |  |
| 1 | Vvi-Vitvi07g00158\_t001 |  | Atr-ERN12365 |  |  |  |  |  |  |  |  |  |  |
| 1 | Vvi-Vitvi07g00159\_t005 |  | Atr-ERN12367 |  |  |  |  |  |  |  |  |  |  |
| 1 | Vvi-Vitvi07g00160\_t001 |  | \| \| |  |  |  |  |  |  |  |  |  |  |
| 1 | Vvi-Vitvi07g00162\_t001 |  | Atr-ERN12369 |  |  |  |  |  |  |  |  |  |  |
| 1 | Vvi-Vitvi07g04045\_t001 |  | \| \| |  |  |  |  |  |  |  |  |  |  |
| 1 | Vvi-Vitvi07g00164\_t001 |  | \| \| |  |  |  |  |  |  |  |  |  |  |
| 1 | Vvi-Vitvi07g00166\_t001 |  | Atr-ERN12371 |  |  |  |  |  |  |  |  |  |  |
| 1 | Vvi-Vitvi07g00167\_t001 |  | \| \| |  |  |  |  |  |  |  |  |  |  |
| 1 | Vvi-Vitvi07g00168\_t001 |  | Atr-ERN12373 |  |  |  |  |  |  |  |  |  |  |
| 1 | Vvi-Vitvi07g00169\_t001 |  | Atr-ERN12378 |  |  |  |  |  |  |  |  |  |  |
| 1 | Vvi-Vitvi07g00171\_t003 |  | Atr-ERN12380 |  |  |  |  |  |  |  |  |  |  |
| 1 | Vvi-Vitvi07g02143\_t001 |  | Atr-ERN12385 |  |  |  |  |  |  |  |  |  |  |
| 1 | Vvi-Vitvi07g02144\_t002 |  | \| \| |  |  |  |  |  |  |  |  |  |  |
| 1 | Vvi-Vitvi07g00172\_t001 |  | \| \| |  |  |  |  |  |  |  |  |  |  |
| 1 | Vvi-Vitvi07g02146\_t001 |  | \| \| |  |  |  |  |  |  |  |  |  |  |
| 1 | Vvi-Vitvi07g00173\_t003 |  | \| \| |  |  |  |  |  |  |  |  |  |  |
| 2 | Vvi-Vitvi07g00174\_t001 |  | \| \| |  | Atr-ERN12697 |  |  |  |  |  |  |  |  |
| 2 | Vvi-Vitvi07g00175\_t001 |  | \| \| |  | Atr-ERN12688 |  |  |  |  |  |  |  |  |
| 2 | Vvi-Vitvi07g00176\_t001 |  | \| \| |  | Atr-ERN12684 |  |  |  |  |  |  |  |  |
| 2 | Vvi-Vitvi07g00177\_t001 |  | \| \| |  | \| \| |  |  |  |  |  |  |  |  |
| 2 | Vvi-Vitvi07g00178\_t001 |  | \| \| |  | Atr-ERN12680 |  |  |  |  |  |  |  |  |
| 2 | Vvi-Vitvi07g00179\_t001.1.6037826e |  | \| \| |  | \| \| |  |  |  |  |  |  |  |  |
| 2 | Vvi-Vitvi07g00181\_t001 |  | \| \| |  | Atr-ERN12673 |  |  |  |  |  |  |  |  |
| 2 | Vvi-Vitvi07g00182\_t001 |  | \| \| |  | \| \| |  |  |  |  |  |  |  |  |
| 2 | Vvi-Vitvi07g04046\_t001 |  | Atr-ERN12387 |  | \| \| |  |  |  |  |  |  |  |  |
| 2 | Vvi-Vitvi07g00184\_t001 |  | Atr-ERN12389 |  | Atr-ERN12670 |  |  |  |  |  |  |  |  |
| 1 | Vvi-Vitvi07g02147\_t001 |  | \| \| |  |  |  |  |  |  |  |  |  |  |
| 1 | Vvi-Vitvi07g00185\_t001 |  | \| \| |  |  |  |  |  |  |  |  |  |  |
| 1 | Vvi-Vitvi07g00186\_t001 |  | Atr-ERN12391 |  |  |  |  |  |  |  |  |  |  |
| 1 | Vvi-Vitvi07g00187\_t001 |  | Atr-ERN12396 |  |  |  |  |  |  |  |  |  |  |
| 1 | Vvi-Vitvi07g00188\_t001 |  | Atr-ERN12403 |  |  |  |  |  |  |  |  |  |  |
| 1 | Vvi-Vitvi07g00189\_t001 |  | Atr-ERN12406 |  |  |  |  |  |  |  |  |  |  |
| 1 | Vvi-Vitvi07g02148\_t001 |  | \| \| |  |  |  |  |  |  |  |  |  |  |
| 1 | Vvi-Vitvi07g02149\_t001 |  | \| \| |  |  |  |  |  |  |  |  |  |  |
| 1 | Vvi-Vitvi07g00190\_t001 |  | Atr-ERN12419 |  |  |  |  |  |  |  |  |  |  |
| 1 | Vvi-Vitvi07g00191\_t001 |  | \| \| |  |  |  |  |  |  |  |  |  |  |
| 1 | Vvi-Vitvi07g04047\_t001 |  | \| \| |  |  |  |  |  |  |  |  |  |  |
| 1 | Vvi-Vitvi07g00193\_t001 |  | Atr-ERN12427 |  |  |  |  |  |  |  |  |  |  |
| 1 | Vvi-Vitvi07g00194\_t001 |  | \| \| |  |  |  |  |  |  |  |  |  |  |
| 1 | Vvi-Vitvi07g00195\_t001 |  | Atr-ERN12428 |  |  |  |  |  |  |  |  |  |  |
| 1 | Vvi-Vitvi07g00196\_t001 |  | Atr-ERN12430 |  |  |  |  |  |  |  |  |  |  |
| 1 | Vvi-Vitvi07g00197\_t001 |  | \| \| |  |  |  |  |  |  |  |  |  |  |
| 1 | Vvi-Vitvi07g00198\_t001 |  | Atr-ERN12444 |  |  |  |  |  |  |  |  |  |  |
| 1 | Vvi-Vitvi07g00199\_t001 |  | Atr-ERN12445 |  |  |  |  |  |  |  |  |  |  |
| 1 | Vvi-Vitvi07g04048\_t001 |  | \| \| |  |  |  |  |  |  |  |  |  |  |
| 1 | Vvi-Vitvi07g00200\_t001 |  | Atr-ERN12447 |  |  |  |  |  |  |  |  |  |  |
| 0 | Vvi-Vitvi07g04049\_t001 |  |  |  |  |  |  |  |  |  |  |  |  |
| 1 | Vvi-Vitvi07g00201\_t001 |  | Atr-ERN07469 |  |  |  |  |  |  |  |  |  |  |
| 1 | Vvi-Vitvi07g00202\_t001 |  | Atr-ERN07471 |  |  |  |  |  |  |  |  |  |  |
| 1 | Vvi-Vitvi07g00203\_t001 |  | \| \| |  |  |  |  |  |  |  |  |  |  |
| 1 | Vvi-Vitvi07g00204\_t002 |  | \| \| |  |  |  |  |  |  |  |  |  |  |
| 1 | Vvi-Vitvi07g00205\_t001 |  | \| \| |  |  |  |  |  |  |  |  |  |  |
| 1 | Vvi-Vitvi07g00207\_t001 |  | Atr-ERN07481 |  |  |  |  |  |  |  |  |  |  |
| 1 | Vvi-Vitvi07g00208\_t001 |  | Atr-ERN07487 |  |  |  |  |  |  |  |  |  |  |
| 1 | Vvi-Vitvi07g00209\_t001 |  | Atr-ERN07492 |  |  |  |  |  |  |  |  |  |  |
| 1 | Vvi-Vitvi07g00210\_t001 |  | Atr-ERN07493 |  |  |  |  |  |  |  |  |  |  |
| 0 | Vvi-Vitvi07g00211\_t001 |  |  |  |  |  |  |  |  |  |  |  |  |
| 0 | Vvi-Vitvi07g00213\_t001 |  |  |  |  |  |  |  |  |  |  |  |  |
| 0 | Vvi-Vitvi07g00214\_t002 |  |  |  |  |  |  |  |  |  |  |  |  |
| 0 | Vvi-Vitvi07g00216\_t001 |  |  |  |  |  |  |  |  |  |  |  |  |
| 0 | Vvi-Vitvi07g00217\_t001 |  |  |  |  |  |  |  |  |  |  |  |  |
| 0 | Vvi-Vitvi07g00218\_t001 |  |  |  |  |  |  |  |  |  |  |  |  |
| 0 | Vvi-Vitvi07g00219\_t001 |  |  |  |  |  |  |  |  |  |  |  |  |
| 0 | Vvi-Vitvi07g00220\_t001 |  |  |  |  |  |  |  |  |  |  |  |  |
| 0 | Vvi-Vitvi07g02153\_t001 |  |  |  |  |  |  |  |  |  |  |  |  |
| 0 | Vvi-Vitvi07g00221\_t001 |  |  |  |  |  |  |  |  |  |  |  |  |
| 0 | Vvi-Vitvi07g04050\_t001 |  |  |  |  |  |  |  |  |  |  |  |  |
| 0 | Vvi-Vitvi07g00223\_t001 |  |  |  |  |  |  |  |  |  |  |  |  |
| 0 | Vvi-Vitvi07g00224\_t001 |  |  |  |  |  |  |  |  |  |  |  |  |
| 0 | Vvi-Vitvi07g04051\_t001 |  |  |  |  |  |  |  |  |  |  |  |  |
| 0 | Vvi-Vitvi07g00225\_t001 |  |  |  |  |  |  |  |  |  |  |  |  |
| 0 | Vvi-Vitvi07g04052\_t001 |  |  |  |  |  |  |  |  |  |  |  |  |
| 0 | Vvi-Vitvi07g00226\_t001 |  |  |  |  |  |  |  |  |  |  |  |  |
| 1 | Vvi-Vitvi07g00227\_t001 |  | Atr-ERN16836 |  |  |  |  |  |  |  |  |  |  |
| 1 | Vvi-Vitvi07g00228\_t001 |  | Atr-ERN16838 |  |  |  |  |  |  |  |  |  |  |
| 1 | Vvi-Vitvi07g00230\_t001 |  | Atr-ERN16844 |  |  |  |  |  |  |  |  |  |  |
| 1 | Vvi-Vitvi07g00231\_t001 |  | Atr-ERN16845 |  |  |  |  |  |  |  |  |  |  |
| 1 | Vvi-Vitvi07g02156\_t001 |  | \| \| |  |  |  |  |  |  |  |  |  |  |
| 1 | Vvi-Vitvi07g04053\_t001 |  | \| \| |  |  |  |  |  |  |  |  |  |  |
| 1 | Vvi-Vitvi07g00232\_t001 |  | Atr-ERN16847 |  |  |  |  |  |  |  |  |  |  |
| 1 | Vvi-Vitvi07g00233\_t001 |  | Atr-ERN16848 |  |  |  |  |  |  |  |  |  |  |
| 1 | Vvi-Vitvi07g00234\_t001 |  | Atr-ERN16850 |  |  |  |  |  |  |  |  |  |  |
| 1 | Vvi-Vitvi07g04054\_t001 |  | \| \| |  |  |  |  |  |  |  |  |  |  |
| 1 | Vvi-Vitvi07g00236\_t001 |  | \| \| |  |  |  |  |  |  |  |  |  |  |
| 1 | Vvi-Vitvi07g00237\_t001 |  | Atr-ERN16859 |  |  |  |  |  |  |  |  |  |  |
| 1 | Vvi-Vitvi07g00238\_t001 |  | Atr-ERN16861 |  |  |  |  |  |  |  |  |  |  |
| 1 | Vvi-Vitvi07g04055\_t001 |  | \| \| |  |  |  |  |  |  |  |  |  |  |
| 1 | Vvi-Vitvi07g00240\_t001 |  | Atr-ERN16863 |  |  |  |  |  |  |  |  |  |  |
| 1 | Vvi-Vitvi07g00241\_t001 |  | \| \| |  |  |  |  |  |  |  |  |  |  |
| 1 | Vvi-Vitvi07g00242\_t001 |  | \| \| |  |  |  |  |  |  |  |  |  |  |
| 1 | Vvi-Vitvi07g02158\_t001 |  | \| \| |  |  |  |  |  |  |  |  |  |  |
| 1 | Vvi-Vitvi07g00243\_t001 |  | Atr-ERN16868 |  |  |  |  |  |  |  |  |  |  |
| 1 | Vvi-Vitvi07g00244\_t001 |  | Atr-ERN16872 |  |  |  |  |  |  |  |  |  |  |
| 1 | Vvi-Vitvi07g04056\_t001 |  | \| \| |  |  |  |  |  |  |  |  |  |  |
| 1 | Vvi-Vitvi07g02159\_t002 |  | Atr-ERN16876 |  |  |  |  |  |  |  |  |  |  |
| 1 | Vvi-Vitvi07g02160\_t001 |  | \| \| |  |  |  |  |  |  |  |  |  |  |
| 1 | Vvi-Vitvi07g00248\_t002 |  | \| \| |  |  |  |  |  |  |  |  |  |  |
| 1 | Vvi-Vitvi07g02161\_t001 |  | \| \| |  |  |  |  |  |  |  |  |  |  |
| 1 | Vvi-Vitvi07g00250\_t001 |  | \| \| |  |  |  |  |  |  |  |  |  |  |
| 1 | Vvi-Vitvi07g00251\_t001 |  | Atr-ERN16883 |  |  |  |  |  |  |  |  |  |  |
| 1 | Vvi-Vitvi07g00252\_t001 |  | \| \| |  |  |  |  |  |  |  |  |  |  |
| 1 | Vvi-Vitvi07g00253\_t001 |  | Atr-ERN16907 |  |  |  |  |  |  |  |  |  |  |
| 1 | Vvi-Vitvi07g02162\_t001 |  | \| \| |  |  |  |  |  |  |  |  |  |  |
| 1 | Vvi-Vitvi07g02163\_t001 |  | \| \| |  |  |  |  |  |  |  |  |  |  |
| 1 | Vvi-Vitvi07g00254\_t001 |  | \| \| |  |  |  |  |  |  |  |  |  |  |
| 1 | Vvi-Vitvi07g04057\_t001 |  | \| \| |  |  |  |  |  |  |  |  |  |  |
| 1 | Vvi-Vitvi07g00255\_t001 |  | Atr-ERN16921 |  |  |  |  |  |  |  |  |  |  |
| 1 | Vvi-Vitvi07g00256\_t001 |  | \| \| |  |  |  |  |  |  |  |  |  |  |
| 1 | Vvi-Vitvi07g00257\_t002 |  | \| \| |  |  |  |  |  |  |  |  |  |  |
| 1 | Vvi-Vitvi07g00259\_t001 |  | \| \| |  |  |  |  |  |  |  |  |  |  |
| 1 | Vvi-Vitvi07g02164\_t001 |  | \| \| |  |  |  |  |  |  |  |  |  |  |
| 1 | Vvi-Vitvi07g00260\_t001 |  | Atr-ERN16941 |  |  |  |  |  |  |  |  |  |  |
| 1 | Vvi-Vitvi07g04058\_t001 |  | \| \| |  |  |  |  |  |  |  |  |  |  |
| 1 | Vvi-Vitvi07g00262\_t002 |  | \| \| |  |  |  |  |  |  |  |  |  |  |
| 1 | Vvi-Vitvi07g04059\_t001 |  | \| \| |  |  |  |  |  |  |  |  |  |  |
| 1 | Vvi-Vitvi07g00264\_t001 |  | \| \| |  |  |  |  |  |  |  |  |  |  |
| 1 | Vvi-Vitvi07g00265\_t001 |  | Atr-ERN16951 |  |  |  |  |  |  |  |  |  |  |
| 1 | Vvi-Vitvi07g02165\_t001 |  | \| \| |  |  |  |  |  |  |  |  |  |  |
| 1 | Vvi-Vitvi07g02166\_t001 |  | \| \| |  |  |  |  |  |  |  |  |  |  |
| 1 | Vvi-Vitvi07g02167\_t001 |  | \| \| |  |  |  |  |  |  |  |  |  |  |
| 1 | Vvi-Vitvi07g04060\_t001 |  | \| \| |  |  |  |  |  |  |  |  |  |  |
| 1 | Vvi-Vitvi07g04061\_t001 |  | \| \| |  |  |  |  |  |  |  |  |  |  |
| 1 | Vvi-Vitvi07g04062\_t001 |  | \| \| |  |  |  |  |  |  |  |  |  |  |
| 1 | Vvi-Vitvi07g04063\_t001 |  | \| \| |  |  |  |  |  |  |  |  |  |  |
| 1 | Vvi-Vitvi07g04064\_t001 |  | \| \| |  |  |  |  |  |  |  |  |  |  |
| 1 | Vvi-Vitvi07g02173\_t001 |  | \| \| |  |  |  |  |  |  |  |  |  |  |
| 1 | Vvi-Vitvi07g02174\_t001 |  | \| \| |  |  |  |  |  |  |  |  |  |  |
| 1 | Vvi-Vitvi07g00266\_t001 |  | \| \| |  |  |  |  |  |  |  |  |  |  |
| 1 | Vvi-Vitvi07g00267\_t001 |  | \| \| |  |  |  |  |  |  |  |  |  |  |
| 1 | Vvi-Vitvi07g00268\_t001 |  | \| \| |  |  |  |  |  |  |  |  |  |  |
| 1 | Vvi-Vitvi07g00269\_t001 |  | Atr-ERN16955 |  |  |  |  |  |  |  |  |  |  |
| 1 | Vvi-Vitvi07g00270\_t001 |  | \| \| |  |  |  |  |  |  |  |  |  |  |
| 1 | Vvi-Vitvi07g00271\_t001 |  | \| \| |  |  |  |  |  |  |  |  |  |  |
| 1 | Vvi-Vitvi07g00272\_t001 |  | \| \| |  |  |  |  |  |  |  |  |  |  |
| 1 | Vvi-Vitvi07g04065\_t001 |  | \| \| |  |  |  |  |  |  |  |  |  |  |
| 1 | Vvi-Vitvi07g00273\_t001 |  | Atr-ERN16970 |  |  |  |  |  |  |  |  |  |  |
| 1 | Vvi-Vitvi07g04066\_t001 |  | \| \| |  |  |  |  |  |  |  |  |  |  |
| 1 | Vvi-Vitvi07g00274\_t001 |  | \| \| |  |  |  |  |  |  |  |  |  |  |
| 1 | Vvi-Vitvi07g04067\_t001 |  | \| \| |  |  |  |  |  |  |  |  |  |  |
| 1 | Vvi-Vitvi07g02178\_t001 |  | \| \| |  |  |  |  |  |  |  |  |  |  |
| 1 | Vvi-Vitvi07g00275\_t001 |  | \| \| |  |  |  |  |  |  |  |  |  |  |
| 1 | Vvi-Vitvi07g04068\_t001 |  | \| \| |  |  |  |  |  |  |  |  |  |  |
| 1 | Vvi-Vitvi07g04069\_t001 |  | \| \| |  |  |  |  |  |  |  |  |  |  |
| 1 | Vvi-Vitvi07g04070\_t001 |  | \| \| |  |  |  |  |  |  |  |  |  |  |
| 1 | Vvi-Vitvi07g04071\_t001 |  | \| \| |  |  |  |  |  |  |  |  |  |  |
| 1 | Vvi-Vitvi07g02181\_t001 |  | \| \| |  |  |  |  |  |  |  |  |  |  |
| 1 | Vvi-Vitvi07g02182\_t001 |  | \| \| |  |  |  |  |  |  |  |  |  |  |
| 1 | Vvi-Vitvi07g02183\_t001 |  | \| \| |  |  |  |  |  |  |  |  |  |  |
| 1 | Vvi-Vitvi07g00276\_t001 |  | \| \| |  |  |  |  |  |  |  |  |  |  |
| 1 | Vvi-Vitvi07g00277\_t001 |  | \| \| |  |  |  |  |  |  |  |  |  |  |
| 1 | Vvi-Vitvi07g00278\_t001 |  | Atr-ERN16990 |  |  |  |  |  |  |  |  |  |  |
| 1 | Vvi-Vitvi07g02184\_t001 |  | \| \| |  |  |  |  |  |  |  |  |  |  |
| 1 | Vvi-Vitvi07g02185\_t001 |  | \| \| |  |  |  |  |  |  |  |  |  |  |
| 1 | Vvi-Vitvi07g00279\_t001 |  | \| \| |  |  |  |  |  |  |  |  |  |  |
| 1 | Vvi-Vitvi07g00280\_t001 |  | Atr-ERN16994 |  |  |  |  |  |  |  |  |  |  |
| 1 | Vvi-Vitvi07g04072\_t001 |  | \| \| |  |  |  |  |  |  |  |  |  |  |
| 1 | Vvi-Vitvi07g00282\_t001 |  | \| \| |  |  |  |  |  |  |  |  |  |  |
| 1 | Vvi-Vitvi07g02188\_t003 |  | Atr-ERN16995 |  |  |  |  |  |  |  |  |  |  |
| 1 | Vvi-Vitvi07g02190\_t001 |  | Atr-ERN17000 |  |  |  |  |  |  |  |  |  |  |
| 1 | Vvi-Vitvi07g00283\_t001 |  | \| \| |  |  |  |  |  |  |  |  |  |  |
| 1 | Vvi-Vitvi07g00284\_t001 |  | \| \| |  |  |  |  |  |  |  |  |  |  |
| 1 | Vvi-Vitvi07g00285\_t001 |  | \| \| |  |  |  |  |  |  |  |  |  |  |
| 1 | Vvi-Vitvi07g02191\_t001 |  | \| \| |  |  |  |  |  |  |  |  |  |  |
| 1 | Vvi-Vitvi07g00286\_t001 |  | \| \| |  |  |  |  |  |  |  |  |  |  |
| 1 | Vvi-Vitvi07g00287\_t001 |  | \| \| |  |  |  |  |  |  |  |  |  |  |
| 1 | Vvi-Vitvi07g00289\_t001 |  | Atr-ERN17010 |  |  |  |  |  |  |  |  |  |  |
| 1 | Vvi-Vitvi07g00290\_t001 |  | Atr-ERN02173 |  |  |  |  |  |  |  |  |  |  |
| 1 | Vvi-Vitvi07g00291\_t001 |  | Atr-ERN02176 |  |  |  |  |  |  |  |  |  |  |
| 1 | Vvi-Vitvi07g02193\_t001 |  | \| \| |  |  |  |  |  |  |  |  |  |  |
| 1 | Vvi-Vitvi07g00292\_t001 |  | \| \| |  |  |  |  |  |  |  |  |  |  |
| 1 | Vvi-Vitvi07g00293\_t001 |  | Atr-ERN02179 |  |  |  |  |  |  |  |  |  |  |
| 1 | Vvi-Vitvi07g00294\_t001 |  | Atr-ERN02181 |  |  |  |  |  |  |  |  |  |  |
| 1 | Vvi-Vitvi07g00295\_t001 |  | Atr-ERN02184 |  |  |  |  |  |  |  |  |  |  |
| 1 | Vvi-Vitvi07g02194\_t001 |  | \| \| |  |  |  |  |  |  |  |  |  |  |
| 1 | Vvi-Vitvi07g00296\_t001 |  | Atr-ERN02185 |  |  |  |  |  |  |  |  |  |  |
| 1 | Vvi-Vitvi07g00298\_t001 |  | Atr-ERN02187 |  |  |  |  |  |  |  |  |  |  |
| 1 | Vvi-Vitvi07g00299\_t001 |  | \| \| |  |  |  |  |  |  |  |  |  |  |
| 1 | Vvi-Vitvi07g00300\_t001 |  | Atr-ERN02189 |  |  |  |  |  |  |  |  |  |  |
| 1 | Vvi-Vitvi07g00301\_t001 |  | Atr-ERN02195 |  |  |  |  |  |  |  |  |  |  |
| 1 | Vvi-Vitvi07g00302\_t001 |  | \| \| |  |  |  |  |  |  |  |  |  |  |
| 1 | Vvi-Vitvi07g04073\_t001 |  | \| \| |  |  |  |  |  |  |  |  |  |  |
| 1 | Vvi-Vitvi07g00303\_t001 |  | \| \| |  |  |  |  |  |  |  |  |  |  |
| 1 | Vvi-Vitvi07g00306\_t002 |  | \| \| |  |  |  |  |  |  |  |  |  |  |
| 1 | Vvi-Vitvi07g00307\_t001 |  | Atr-ERN02217 |  |  |  |  |  |  |  |  |  |  |
| 1 | Vvi-Vitvi07g00308\_t002 |  | Atr-ERN02219 |  |  |  |  |  |  |  |  |  |  |
| 1 | Vvi-Vitvi07g00309\_t001 |  | Atr-ERN02221 |  |  |  |  |  |  |  |  |  |  |
| 1 | Vvi-Vitvi07g00310\_t003 |  | Atr-ERN02222 |  |  |  |  |  |  |  |  |  |  |
| 1 | Vvi-Vitvi07g04074\_t001 |  | \| \| |  |  |  |  |  |  |  |  |  |  |
| 1 | Vvi-Vitvi07g00311\_t001 |  | Atr-ERN02230 |  |  |  |  |  |  |  |  |  |  |
| 1 | Vvi-Vitvi07g00312\_t001 |  | Atr-ERN02235 |  |  |  |  |  |  |  |  |  |  |
| 1 | Vvi-Vitvi07g00313\_t001 |  | Atr-ERN02238 |  |  |  |  |  |  |  |  |  |  |
| 1 | Vvi-Vitvi07g00314\_t001 |  | Atr-ERN02239 |  |  |  |  |  |  |  |  |  |  |
| 1 | Vvi-Vitvi07g04075\_t001 |  | \| \| |  |  |  |  |  |  |  |  |  |  |
| 1 | Vvi-Vitvi07g00315\_t001 |  | \| \| |  |  |  |  |  |  |  |  |  |  |
| 1 | Vvi-Vitvi07g00316\_t001 |  | \| \| |  |  |  |  |  |  |  |  |  |  |
| 1 | Vvi-Vitvi07g00317\_t001 |  | \| \| |  |  |  |  |  |  |  |  |  |  |
| 1 | Vvi-Vitvi07g02198\_t001 |  | Atr-ERN02246 |  |  |  |  |  |  |  |  |  |  |
| 1 | Vvi-Vitvi07g02201\_t002 |  | \| \| |  |  |  |  |  |  |  |  |  |  |
| 1 | Vvi-Vitvi07g04076\_t001 |  | \| \| |  |  |  |  |  |  |  |  |  |  |
| 1 | Vvi-Vitvi07g04077\_t001 |  | \| \| |  |  |  |  |  |  |  |  |  |  |
| 1 | Vvi-Vitvi07g04078\_t001 |  | \| \| |  |  |  |  |  |  |  |  |  |  |
| 1 | Vvi-Vitvi07g00320\_t003 |  | \| \| |  |  |  |  |  |  |  |  |  |  |
| 1 | Vvi-Vitvi07g02202\_t001 |  | \| \| |  |  |  |  |  |  |  |  |  |  |
| 1 | Vvi-Vitvi07g04079\_t001 |  | \| \| |  |  |  |  |  |  |  |  |  |  |
| 1 | Vvi-Vitvi07g04080\_t001 |  | \| \| |  |  |  |  |  |  |  |  |  |  |
| 1 | Vvi-Vitvi07g04081\_t001 |  | \| \| |  |  |  |  |  |  |  |  |  |  |
| 1 | Vvi-Vitvi07g00321\_t001 |  | \| \| |  |  |  |  |  |  |  |  |  |  |
| 1 | Vvi-Vitvi07g02203\_t001 |  | Atr-ERN02256 |  |  |  |  |  |  |  |  |  |  |
| 1 | Vvi-Vitvi07g00322\_t001 |  | Atr-ERN02257 |  |  |  |  |  |  |  |  |  |  |
| 0 | Vvi-Vitvi07g00323\_t001 |  |  |  |  |  |  |  |  |  |  |  |  |
| 0 | Vvi-Vitvi07g00324\_t001 |  |  |  |  |  |  |  |  |  |  |  |  |
| 0 | Vvi-Vitvi07g00325\_t001 |  |  |  |  |  |  |  |  |  |  |  |  |
| 0 | Vvi-Vitvi07g04082\_t001 |  |  |  |  |  |  |  |  |  |  |  |  |
| 0 | Vvi-Vitvi07g00327\_t001 |  |  |  |  |  |  |  |  |  |  |  |  |
| 0 | Vvi-Vitvi07g04083\_t001 |  |  |  |  |  |  |  |  |  |  |  |  |
| 0 | Vvi-Vitvi07g00328\_t001 |  |  |  |  |  |  |  |  |  |  |  |  |
| 0 | Vvi-Vitvi07g02204\_t001 |  |  |  |  |  |  |  |  |  |  |  |  |
| 0 | Vvi-Vitvi07g00329\_t001 |  |  |  |  |  |  |  |  |  |  |  |  |
| 0 | Vvi-Vitvi07g04084\_t001 |  |  |  |  |  |  |  |  |  |  |  |  |
| 0 | Vvi-Vitvi07g00330\_t001 |  |  |  |  |  |  |  |  |  |  |  |  |
| 0 | Vvi-Vitvi07g00331\_t001 |  |  |  |  |  |  |  |  |  |  |  |  |
| 0 | Vvi-Vitvi07g00332\_t001 |  |  |  |  |  |  |  |  |  |  |  |  |
| 0 | Vvi-Vitvi07g00333\_t001 |  |  |  |  |  |  |  |  |  |  |  |  |
| 0 | Vvi-Vitvi07g00334\_t001 |  |  |  |  |  |  |  |  |  |  |  |  |
| 0 | Vvi-Vitvi07g00335\_t001.1.6037826e |  |  |  |  |  |  |  |  |  |  |  |  |
| 0 | Vvi-Vitvi07g00336\_t001 |  |  |  |  |  |  |  |  |  |  |  |  |
| 0 | Vvi-Vitvi07g00337\_t001 |  |  |  |  |  |  |  |  |  |  |  |  |
| 0 | Vvi-Vitvi07g00338\_t001 |  |  |  |  |  |  |  |  |  |  |  |  |
| 0 | Vvi-Vitvi07g00339\_t001 |  |  |  |  |  |  |  |  |  |  |  |  |
| 0 | Vvi-Vitvi07g00340\_t001 |  |  |  |  |  |  |  |  |  |  |  |  |
| 0 | Vvi-Vitvi07g00341\_t001 |  |  |  |  |  |  |  |  |  |  |  |  |
| 0 | Vvi-Vitvi07g00342\_t001 |  |  |  |  |  |  |  |  |  |  |  |  |
| 0 | Vvi-Vitvi07g00343\_t001 |  |  |  |  |  |  |  |  |  |  |  |  |
| 0 | Vvi-Vitvi07g00346\_t001 |  |  |  |  |  |  |  |  |  |  |  |  |
| 0 | Vvi-Vitvi07g00347\_t001 |  |  |  |  |  |  |  |  |  |  |  |  |
| 0 | Vvi-Vitvi07g00348\_t001 |  |  |  |  |  |  |  |  |  |  |  |  |
| 0 | Vvi-Vitvi07g00349\_t001 |  |  |  |  |  |  |  |  |  |  |  |  |
| 1 | Vvi-Vitvi07g00350\_t001 |  | Atr-ERN03161 |  |  |  |  |  |  |  |  |  |  |
| 1 | Vvi-Vitvi07g00351\_t001 |  | \| \| |  |  |  |  |  |  |  |  |  |  |
| 1 | Vvi-Vitvi07g04085\_t001 |  | \| \| |  |  |  |  |  |  |  |  |  |  |
| 1 | Vvi-Vitvi07g00352\_t001 |  | \| \| |  |  |  |  |  |  |  |  |  |  |
| 1 | Vvi-Vitvi07g00353\_t001 |  | \| \| |  |  |  |  |  |  |  |  |  |  |
| 1 | Vvi-Vitvi07g00354\_t001 |  | \| \| |  |  |  |  |  |  |  |  |  |  |
| 1 | Vvi-Vitvi07g02206\_t001 |  | \| \| |  |  |  |  |  |  |  |  |  |  |
| 1 | Vvi-Vitvi07g00355\_t001 |  | Atr-ERN03174 |  |  |  |  |  |  |  |  |  |  |
| 1 | Vvi-Vitvi07g02207\_t001 |  | \| \| |  |  |  |  |  |  |  |  |  |  |
| 1 | Vvi-Vitvi07g02208\_t001 |  | Atr-ERN03178 |  |  |  |  |  |  |  |  |  |  |
| 1 | Vvi-Vitvi07g00356\_t001 |  | \| \| |  |  |  |  |  |  |  |  |  |  |
| 1 | Vvi-Vitvi07g02209\_t001 |  | \| \| |  |  |  |  |  |  |  |  |  |  |
| 1 | Vvi-Vitvi07g04086\_t001 |  | \| \| |  |  |  |  |  |  |  |  |  |  |
| 1 | Vvi-Vitvi07g00357\_t001 |  | Atr-ERN03179 |  |  |  |  |  |  |  |  |  |  |
| 1 | Vvi-Vitvi07g00358\_t001 |  | Atr-ERN03185 |  |  |  |  |  |  |  |  |  |  |
| 1 | Vvi-Vitvi07g02210\_t001 |  | \| \| |  |  |  |  |  |  |  |  |  |  |
| 1 | Vvi-Vitvi07g00359\_t001 |  | \| \| |  |  |  |  |  |  |  |  |  |  |
| 1 | Vvi-Vitvi07g04087\_t001 |  | \| \| |  |  |  |  |  |  |  |  |  |  |
| 1 | Vvi-Vitvi07g00360\_t001 |  | \| \| |  |  |  |  |  |  |  |  |  |  |
| 1 | Vvi-Vitvi07g00361\_t001 |  | \| \| |  |  |  |  |  |  |  |  |  |  |
| 1 | Vvi-Vitvi07g00362\_t001 |  | \| \| |  |  |  |  |  |  |  |  |  |  |
| 1 | Vvi-Vitvi07g00363\_t001 |  | Atr-ERN03187 |  |  |  |  |  |  |  |  |  |  |
| 1 | Vvi-Vitvi07g00364\_t001 |  | \| \| |  |  |  |  |  |  |  |  |  |  |
| 1 | Vvi-Vitvi07g00365\_t001 |  | Atr-ERN03196 |  |  |  |  |  |  |  |  |  |  |
| 0 | Vvi-Vitvi07g00366\_t001 |  |  |  |  |  |  |  |  |  |  |  |  |
| 0 | Vvi-Vitvi07g00367\_t001 |  |  |  |  |  |  |  |  |  |  |  |  |
| 1 | Vvi-Vitvi07g00368\_t001 |  | Atr-ERN16808 |  |  |  |  |  |  |  |  |  |  |
| 1 | Vvi-Vitvi07g04088\_t001 |  | \| \| |  |  |  |  |  |  |  |  |  |  |
| 1 | Vvi-Vitvi07g00369\_t001 |  | Atr-ERN16812 |  |  |  |  |  |  |  |  |  |  |
| 1 | Vvi-Vitvi07g00370\_t001 |  | Atr-ERN16816 |  |  |  |  |  |  |  |  |  |  |
| 1 | Vvi-Vitvi07g00371\_t001 |  | \| \| |  |  |  |  |  |  |  |  |  |  |
| 2 | Vvi-Vitvi07g04089\_t001 |  | \| \| |  | Atr-ERN16823 |  |  |  |  |  |  |  |  |
| 2 | Vvi-Vitvi07g00373\_t001 |  | Atr-ERN16822 |  | \| \| |  |  |  |  |  |  |  |  |
| 2 | Vvi-Vitvi07g00374\_t001 |  | \| \| |  | Atr-ERN16820 |  |  |  |  |  |  |  |  |
| 2 | Vvi-Vitvi07g00375\_t001 |  | Atr-ERN16824 |  | \| \| |  |  |  |  |  |  |  |  |
| 2 | Vvi-Vitvi07g00376\_t001 |  | Atr-ERN16829 |  | \| \| |  |  |  |  |  |  |  |  |
| 2 | Vvi-Vitvi07g00378\_t001 |  | Atr-ERN16830 |  | \| \| |  |  |  |  |  |  |  |  |
| 2 | Vvi-Vitvi07g04090\_t001 |  | \| \| |  | \| \| |  |  |  |  |  |  |  |  |
| 2 | Vvi-Vitvi07g02211\_t001 |  | \| \| |  | \| \| |  |  |  |  |  |  |  |  |
| 2 | Vvi-Vitvi07g02212\_t001 |  | \| \| |  | \| \| |  |  |  |  |  |  |  |  |
| 2 | Vvi-Vitvi07g02213\_t001 |  | \| \| |  | \| \| |  |  |  |  |  |  |  |  |
| 2 | Vvi-Vitvi07g00380\_t001 |  | \| \| |  | \| \| |  |  |  |  |  |  |  |  |
| 2 | Vvi-Vitvi07g02214\_t001 |  | Atr-ERN16834 |  | \| \| |  |  |  |  |  |  |  |  |
| 1 | Vvi-Vitvi07g00381\_t001 |  |  |  | \| \| |  |  |  |  |  |  |  |  |
| 1 | Vvi-Vitvi07g00382\_t001 |  |  |  | \| \| |  |  |  |  |  |  |  |  |
| 1 | Vvi-Vitvi07g04091\_t001 |  |  |  | \| \| |  |  |  |  |  |  |  |  |
| 1 | Vvi-Vitvi07g00383\_t001 |  |  |  | Atr-ERN16803 |  |  |  |  |  |  |  |  |
| 1 | Vvi-Vitvi07g00384\_t001 |  |  |  | \| \| |  |  |  |  |  |  |  |  |
| 1 | Vvi-Vitvi07g02216\_t001 |  |  |  | Atr-ERN16791 |  |  |  |  |  |  |  |  |
| 1 | Vvi-Vitvi07g02217\_t001 |  |  |  | Atr-ERN16788 |  |  |  |  |  |  |  |  |
| 1 | Vvi-Vitvi07g00386\_t001 |  |  |  | Atr-ERN16787 |  |  |  |  |  |  |  |  |
| 1 | Vvi-Vitvi07g00387\_t001 |  |  |  | Atr-ERN16786 |  |  |  |  |  |  |  |  |
| 1 | Vvi-Vitvi07g00388\_t001 |  |  |  | \| \| |  |  |  |  |  |  |  |  |
| 1 | Vvi-Vitvi07g00389\_t001 |  |  |  | \| \| |  |  |  |  |  |  |  |  |
| 1 | Vvi-Vitvi07g00390\_t001 |  |  |  | Atr-ERN16782 |  |  |  |  |  |  |  |  |
| 1 | Vvi-Vitvi07g00391\_t001 |  |  |  | Atr-ERN16781 |  |  |  |  |  |  |  |  |
| 1 | Vvi-Vitvi07g00392\_t001 |  |  |  | Atr-ERN16778 |  |  |  |  |  |  |  |  |
| 1 | Vvi-Vitvi07g00393\_t001 |  |  |  | \| \| |  |  |  |  |  |  |  |  |
| 1 | Vvi-Vitvi07g04092\_t001 |  |  |  | \| \| |  |  |  |  |  |  |  |  |
| 1 | Vvi-Vitvi07g00394\_t001 |  |  |  | Atr-ERN16775 |  |  |  |  |  |  |  |  |
| 1 | Vvi-Vitvi07g02218\_t003 |  |  |  | \| \| |  |  |  |  |  |  |  |  |
| 1 | Vvi-Vitvi07g04093\_t001 |  |  |  | \| \| |  |  |  |  |  |  |  |  |
| 1 | Vvi-Vitvi07g00395\_t001 |  |  |  | Atr-ERN16773 |  |  |  |  |  |  |  |  |
| 1 | Vvi-Vitvi07g04094\_t001 |  |  |  | \| \| |  |  |  |  |  |  |  |  |
| 1 | Vvi-Vitvi07g00396\_t001 |  |  |  | Atr-ERN16769 |  |  |  |  |  |  |  |  |
| 1 | Vvi-Vitvi07g00399\_t001 |  |  |  | Atr-ERN16768 |  |  |  |  |  |  |  |  |
| 1 | Vvi-Vitvi07g04095\_t001 |  |  |  | \| \| |  |  |  |  |  |  |  |  |
| 1 | Vvi-Vitvi07g04096\_t001 |  |  |  | \| \| |  |  |  |  |  |  |  |  |
| 1 | Vvi-Vitvi07g00400\_t001 |  |  |  | Atr-ERN16764 |  |  |  |  |  |  |  |  |
| 1 | Vvi-Vitvi07g00401\_t001 |  |  |  | Atr-ERN16761 |  |  |  |  |  |  |  |  |
| 1 | Vvi-Vitvi07g02220\_t001 |  |  |  | Atr-ERN16757 |  |  |  |  |  |  |  |  |
| 1 | Vvi-Vitvi07g02221\_t001 |  |  |  | Atr-ERN16755 |  |  |  |  |  |  |  |  |
| 1 | Vvi-Vitvi07g00402\_t001 |  |  |  | \| \| |  |  |  |  |  |  |  |  |
| 1 | Vvi-Vitvi07g00403\_t001 |  |  |  | \| \| |  |  |  |  |  |  |  |  |
| 1 | Vvi-Vitvi07g00404\_t001 |  |  |  | \| \| |  |  |  |  |  |  |  |  |
| 1 | Vvi-Vitvi07g00405\_t001 |  |  |  | \| \| |  |  |  |  |  |  |  |  |
| 1 | Vvi-Vitvi07g00406\_t001 |  |  |  | \| \| |  |  |  |  |  |  |  |  |
| 1 | Vvi-Vitvi07g04097\_t001 |  |  |  | \| \| |  |  |  |  |  |  |  |  |
| 1 | Vvi-Vitvi07g00407\_t001 |  |  |  | \| \| |  |  |  |  |  |  |  |  |
| 1 | Vvi-Vitvi07g00408\_t001 |  |  |  | \| \| |  |  |  |  |  |  |  |  |
| 1 | Vvi-Vitvi07g02222\_t001 |  |  |  | \| \| |  |  |  |  |  |  |  |  |
| 1 | Vvi-Vitvi07g00409\_t001 |  |  |  | \| \| |  |  |  |  |  |  |  |  |
| 1 | Vvi-Vitvi07g00410\_t002 |  |  |  | Atr-ERN16745 |  |  |  |  |  |  |  |  |
| 0 | Vvi-Vitvi07g00411\_t001 |  |  |  |  |  |  |  |  |  |  |  |  |
| 1 | Vvi-Vitvi07g00412\_t001 |  | Atr-ERN02137 |  |  |  |  |  |  |  |  |  |  |
| 1 | Vvi-Vitvi07g00413\_t001 |  | \| \| |  |  |  |  |  |  |  |  |  |  |
| 1 | Vvi-Vitvi07g04098\_t001 |  | \| \| |  |  |  |  |  |  |  |  |  |  |
| 1 | Vvi-Vitvi07g00414\_t003 |  | Atr-ERN02133 |  |  |  |  |  |  |  |  |  |  |
| 1 | Vvi-Vitvi07g02224\_t001 |  | \| \| |  |  |  |  |  |  |  |  |  |  |
| 1 | Vvi-Vitvi07g00415\_t001 |  | Atr-ERN02127 |  |  |  |  |  |  |  |  |  |  |
| 1 | Vvi-Vitvi07g00416\_t001 |  | Atr-ERN02122 |  |  |  |  |  |  |  |  |  |  |
| 1 | Vvi-Vitvi07g00417\_t001 |  | Atr-ERN02121 |  |  |  |  |  |  |  |  |  |  |
| 1 | Vvi-Vitvi07g00418\_t001 |  | Atr-ERN02114 |  |  |  |  |  |  |  |  |  |  |
| 1 | Vvi-Vitvi07g04099\_t001 |  | Atr-ERN02113 |  |  |  |  |  |  |  |  |  |  |
| 1 | Vvi-Vitvi07g04100\_t001 |  | \| \| |  |  |  |  |  |  |  |  |  |  |
| 1 | Vvi-Vitvi07g00420\_t001 |  | \| \| |  |  |  |  |  |  |  |  |  |  |
| 1 | Vvi-Vitvi07g00421\_t003 |  | Atr-ERN02112 |  |  |  |  |  |  |  |  |  |  |
| 1 | Vvi-Vitvi07g00422\_t001 |  | Atr-ERN02111 |  |  |  |  |  |  |  |  |  |  |
| 1 | Vvi-Vitvi07g02226\_t001 |  | \| \| |  |  |  |  |  |  |  |  |  |  |
| 1 | Vvi-Vitvi07g04101\_t001 |  | \| \| |  |  |  |  |  |  |  |  |  |  |
| 1 | Vvi-Vitvi07g00423\_t001.3.6037826e |  | Atr-ERN02107 |  |  |  |  |  |  |  |  |  |  |
| 1 | Vvi-Vitvi07g02228\_t001 |  | Atr-ERN02102 |  |  |  |  |  |  |  |  |  |  |
| 1 | Vvi-Vitvi07g00424\_t001 |  | Atr-ERN02100 |  |  |  |  |  |  |  |  |  |  |
| 0 | Vvi-Vitvi07g00425\_t001 |  |  |  |  |  |  |  |  |  |  |  |  |
| 0 | Vvi-Vitvi07g00426\_t001 |  |  |  |  |  |  |  |  |  |  |  |  |
| 0 | Vvi-Vitvi07g00427\_t001 |  |  |  |  |  |  |  |  |  |  |  |  |
| 0 | Vvi-Vitvi07g00428\_t001 |  |  |  |  |  |  |  |  |  |  |  |  |
| 0 | Vvi-Vitvi07g00429\_t001 |  |  |  |  |  |  |  |  |  |  |  |  |
| 0 | Vvi-Vitvi07g00430\_t001 |  |  |  |  |  |  |  |  |  |  |  |  |
| 0 | Vvi-Vitvi07g02229\_t001 |  |  |  |  |  |  |  |  |  |  |  |  |
| 0 | Vvi-Vitvi07g00431\_t001 |  |  |  |  |  |  |  |  |  |  |  |  |
| 0 | Vvi-Vitvi07g04102\_t001 |  |  |  |  |  |  |  |  |  |  |  |  |
| 0 | Vvi-Vitvi07g04103\_t001 |  |  |  |  |  |  |  |  |  |  |  |  |
| 1 | Vvi-Vitvi07g02230\_t001 |  | Atr-ERN03302 |  |  |  |  |  |  |  |  |  |  |
| 1 | Vvi-Vitvi07g00434\_t001 |  | Atr-ERN03304 |  |  |  |  |  |  |  |  |  |  |
| 1 | Vvi-Vitvi07g00435\_t001 |  | \| \| |  |  |  |  |  |  |  |  |  |  |
| 1 | Vvi-Vitvi07g04104\_t001 |  | \| \| |  |  |  |  |  |  |  |  |  |  |
| 2 | Vvi-Vitvi07g00436\_t001 |  | \| \| |  | Atr-ERN03316 |  |  |  |  |  |  |  |  |
| 2 | Vvi-Vitvi07g04105\_t001 |  | \| \| |  | \| \| |  |  |  |  |  |  |  |  |
| 2 | Vvi-Vitvi07g00437\_t001 |  | Atr-ERN03306 |  | \| \| |  |  |  |  |  |  |  |  |
| 2 | Vvi-Vitvi07g00438\_t001 |  | \| \| |  | \| \| |  |  |  |  |  |  |  |  |
| 2 | Vvi-Vitvi07g00439\_t001 |  | Atr-ERN03309 |  | Atr-ERN03309 |  |  |  |  |  |  |  |  |
| 2 | Vvi-Vitvi07g00441\_t001 |  | Atr-ERN03317 |  | \| \| |  |  |  |  |  |  |  |  |
| 2 | Vvi-Vitvi07g04106\_t001 |  | \| \| |  | \| \| |  |  |  |  |  |  |  |  |
| 2 | Vvi-Vitvi07g00442\_t001 |  | \| \| |  | \| \| |  |  |  |  |  |  |  |  |
| 2 | Vvi-Vitvi07g00444\_t001 |  | \| \| |  | \| \| |  |  |  |  |  |  |  |  |
| 2 | Vvi-Vitvi07g04107\_t001 |  | \| \| |  | \| \| |  |  |  |  |  |  |  |  |
| 2 | Vvi-Vitvi07g00445\_t001 |  | \| \| |  | \| \| |  |  |  |  |  |  |  |  |
| 2 | Vvi-Vitvi07g00446\_t001 |  | \| \| |  | \| \| |  |  |  |  |  |  |  |  |
| 2 | Vvi-Vitvi07g00448\_t001 |  | \| \| |  | \| \| |  |  |  |  |  |  |  |  |
| 2 | Vvi-Vitvi07g00449\_t001 |  | \| \| |  | \| \| |  |  |  |  |  |  |  |  |
| 2 | Vvi-Vitvi07g00450\_t001 |  | \| \| |  | Atr-ERN03296 |  |  |  |  |  |  |  |  |
| 2 | Vvi-Vitvi07g02231\_t001 |  | \| \| |  | \| \| |  |  |  |  |  |  |  |  |
| 2 | Vvi-Vitvi07g04108\_t001 |  | \| \| |  | \| \| |  |  |  |  |  |  |  |  |
| 2 | Vvi-Vitvi07g04109\_t001 |  | \| \| |  | \| \| |  |  |  |  |  |  |  |  |
| 2 | Vvi-Vitvi07g04110\_t001 |  | \| \| |  | \| \| |  |  |  |  |  |  |  |  |
| 2 | Vvi-Vitvi07g02233\_t001 |  | \| \| |  | \| \| |  |  |  |  |  |  |  |  |
| 2 | Vvi-Vitvi07g02234\_t001 |  | \| \| |  | \| \| |  |  |  |  |  |  |  |  |
| 2 | Vvi-Vitvi07g00452\_t003 |  | \| \| |  | \| \| |  |  |  |  |  |  |  |  |
| 2 | Vvi-Vitvi07g00453\_t001 |  | \| \| |  | Atr-ERN03292 |  |  |  |  |  |  |  |  |
| 2 | Vvi-Vitvi07g00454\_t001 |  | \| \| |  | \| \| |  |  |  |  |  |  |  |  |
| 2 | Vvi-Vitvi07g00455\_t001 |  | \| \| |  | Atr-ERN03288 |  |  |  |  |  |  |  |  |
| 2 | Vvi-Vitvi07g00456\_t001 |  | \| \| |  | Atr-ERN03283 |  |  |  |  |  |  |  |  |
| 2 | Vvi-Vitvi07g00457\_t001 |  | \| \| |  | Atr-ERN03279 |  |  |  |  |  |  |  |  |
| 2 | Vvi-Vitvi07g00458\_t001 |  | Atr-ERN03325 |  | \| \| |  |  |  |  |  |  |  |  |
| 1 | Vvi-Vitvi07g00459\_t001 |  |  |  | \| \| |  |  |  |  |  |  |  |  |
| 1 | Vvi-Vitvi07g04111\_t001 |  |  |  | \| \| |  |  |  |  |  |  |  |  |
| 1 | Vvi-Vitvi07g00460\_t001 |  |  |  | \| \| |  |  |  |  |  |  |  |  |
| 1 | Vvi-Vitvi07g00463\_t001 |  |  |  | Atr-ERN03276 |  |  |  |  |  |  |  |  |
| 1 | Vvi-Vitvi07g00464\_t001 |  |  |  | Atr-ERN03275 |  |  |  |  |  |  |  |  |
| 1 | Vvi-Vitvi07g00465\_t001 |  |  |  | Atr-ERN03272 |  |  |  |  |  |  |  |  |
| 1 | Vvi-Vitvi07g04112\_t001 |  |  |  | \| \| |  |  |  |  |  |  |  |  |
| 1 | Vvi-Vitvi07g04113\_t001 |  |  |  | Atr-ERN03271 |  |  |  |  |  |  |  |  |
| 1 | Vvi-Vitvi07g00467\_t001 |  |  |  | \| \| |  |  |  |  |  |  |  |  |
| 1 | Vvi-Vitvi07g00468\_t001 |  |  |  | \| \| |  |  |  |  |  |  |  |  |
| 1 | Vvi-Vitvi07g00469\_t001 |  |  |  | \| \| |  |  |  |  |  |  |  |  |
| 1 | Vvi-Vitvi07g00470\_t001 |  |  |  | Atr-ERN03269 |  |  |  |  |  |  |  |  |
| 1 | Vvi-Vitvi07g02243\_t001 |  |  |  | \| \| |  |  |  |  |  |  |  |  |
| 1 | Vvi-Vitvi07g02244\_t001 |  |  |  | \| \| |  |  |  |  |  |  |  |  |
| 2 | Vvi-Vitvi07g00471\_t001 |  | Atr-ERN02105 |  | Atr-ERN03258 |  |  |  |  |  |  |  |  |
| 2 | Vvi-Vitvi07g00472\_t002 |  | \| \| |  | Atr-ERN03257 |  |  |  |  |  |  |  |  |
| 2 | Vvi-Vitvi07g04114\_t001 |  | \| \| |  | \| \| |  |  |  |  |  |  |  |  |
| 2 | Vvi-Vitvi07g00474\_t001 |  | \| \| |  | Atr-ERN03254 |  |  |  |  |  |  |  |  |
| 2 | Vvi-Vitvi07g00475\_t001 |  | \| \| |  | Atr-ERN03250 |  |  |  |  |  |  |  |  |
| 3 | Vvi-Vitvi07g00476\_t001 |  | \| \| |  | \| \| |  | Atr-ERN01962 |  |  |  |  |  |  |
| 3 | Vvi-Vitvi07g00477\_t001 |  | \| \| |  | \| \| |  | Atr-ERN01966 |  |  |  |  |  |  |
| 3 | Vvi-Vitvi07g00478\_t001 |  | \| \| |  | \| \| |  | Atr-ERN01967 |  |  |  |  |  |  |
| 3 | Vvi-Vitvi07g00479\_t001 |  | \| \| |  | \| \| |  | \| \| |  |  |  |  |  |  |
| 3 | Vvi-Vitvi07g04115\_t001 |  | \| \| |  | \| \| |  | \| \| |  |  |  |  |  |  |
| 3 | Vvi-Vitvi07g00481\_t001 |  | \| \| |  | \| \| |  | \| \| |  |  |  |  |  |  |
| 3 | Vvi-Vitvi07g02245\_t001 |  | \| \| |  | Atr-ERN03243 |  | \| \| |  |  |  |  |  |  |
| 3 | Vvi-Vitvi07g00482\_t001 |  | \| \| |  | \| \| |  | \| \| |  |  |  |  |  |  |
| 3 | Vvi-Vitvi07g00483\_t001 |  | \| \| |  | \| \| |  | \| \| |  |  |  |  |  |  |
| 3 | Vvi-Vitvi07g00484\_t002 |  | \| \| |  | \| \| |  | \| \| |  |  |  |  |  |  |
| 3 | Vvi-Vitvi07g00485\_t001 |  | \| \| |  | Atr-ERN03240 |  | \| \| |  |  |  |  |  |  |
| 2 | Vvi-Vitvi07g00486\_t001 |  | \| \| |  |  |  | Atr-ERN01976 |  |  |  |  |  |  |
| 2 | Vvi-Vitvi07g02246\_t001 |  | \| \| |  |  |  | Atr-ERN01979 |  |  |  |  |  |  |
| 2 | Vvi-Vitvi07g02247\_t001 |  | \| \| |  |  |  | Atr-ERN01983 |  |  |  |  |  |  |
| 2 | Vvi-Vitvi07g00487\_t001 |  | \| \| |  |  |  | \| \| |  |  |  |  |  |  |
| 2 | Vvi-Vitvi07g00488\_t001 |  | \| \| |  |  |  | \| \| |  |  |  |  |  |  |
| 2 | Vvi-Vitvi07g00490\_t001 |  | \| \| |  |  |  | \| \| |  |  |  |  |  |  |
| 2 | Vvi-Vitvi07g00491\_t001 |  | \| \| |  |  |  | Atr-ERN01987 |  |  |  |  |  |  |
| 2 | Vvi-Vitvi07g00492\_t001 |  | Atr-ERN02100 |  |  |  | Atr-ERN01990 |  |  |  |  |  |  |
| 2 | Vvi-Vitvi07g04116\_t001 |  | \| \| |  |  |  | \| \| |  |  |  |  |  |  |
| 2 | Vvi-Vitvi07g00493\_t001 |  | \| \| |  |  |  | \| \| |  |  |  |  |  |  |
| 2 | Vvi-Vitvi07g00494\_t001 |  | \| \| |  |  |  | Atr-ERN01994 |  |  |  |  |  |  |
| 2 | Vvi-Vitvi07g00495\_t001 |  | \| \| |  |  |  | Atr-ERN01999 |  |  |  |  |  |  |
| 1 | Vvi-Vitvi07g04117\_t001 |  | \| \| |  |  |  |  |  |  |  |  |  |  |
| 1 | Vvi-Vitvi07g00496\_t001 |  | \| \| |  |  |  |  |  |  |  |  |  |  |
| 1 | Vvi-Vitvi07g00497\_t001 |  | \| \| |  |  |  |  |  |  |  |  |  |  |
| 1 | Vvi-Vitvi07g04118\_t001 |  | \| \| |  |  |  |  |  |  |  |  |  |  |
| 1 | Vvi-Vitvi07g02248\_t001 |  | \| \| |  |  |  |  |  |  |  |  |  |  |
| 1 | Vvi-Vitvi07g00502\_t001 |  | Atr-ERN02090 |  |  |  |  |  |  |  |  |  |  |
| 1 | Vvi-Vitvi07g04119\_t001 |  | \| \| |  |  |  |  |  |  |  |  |  |  |
| 1 | Vvi-Vitvi07g04120\_t001 |  | \| \| |  |  |  |  |  |  |  |  |  |  |
| 1 | Vvi-Vitvi07g04121\_t001 |  | \| \| |  |  |  |  |  |  |  |  |  |  |
| 1 | Vvi-Vitvi07g00505\_t001 |  | \| \| |  |  |  |  |  |  |  |  |  |  |
| 1 | Vvi-Vitvi07g00506\_t002 |  | \| \| |  |  |  |  |  |  |  |  |  |  |
| 1 | Vvi-Vitvi07g00507\_t001 |  | \| \| |  |  |  |  |  |  |  |  |  |  |
| 1 | Vvi-Vitvi07g00508\_t001 |  | \| \| |  |  |  |  |  |  |  |  |  |  |
| 1 | Vvi-Vitvi07g00509\_t001 |  | \| \| |  |  |  |  |  |  |  |  |  |  |
| 1 | Vvi-Vitvi07g04122\_t001 |  | \| \| |  |  |  |  |  |  |  |  |  |  |
| 1 | Vvi-Vitvi07g00510\_t001 |  | \| \| |  |  |  |  |  |  |  |  |  |  |
| 1 | Vvi-Vitvi07g02249\_t003 |  | Atr-ERN02080 |  |  |  |  |  |  |  |  |  |  |
| 1 | Vvi-Vitvi07g02074\_t001 |  | Atr-ERN02079 |  |  |  |  |  |  |  |  |  |  |
| 1 | Vvi-Vitvi07g04123\_t001 |  | \| \| |  |  |  |  |  |  |  |  |  |  |
| 1 | Vvi-Vitvi07g00513\_t001 |  | Atr-ERN02077 |  |  |  |  |  |  |  |  |  |  |
| 1 | Vvi-Vitvi07g00515\_t001 |  | Atr-ERN02076 |  |  |  |  |  |  |  |  |  |  |
| 1 | Vvi-Vitvi07g00516\_t001 |  | \| \| |  |  |  |  |  |  |  |  |  |  |
| 1 | Vvi-Vitvi07g00517\_t001 |  | Atr-ERN02067 |  |  |  |  |  |  |  |  |  |  |
| 1 | Vvi-Vitvi07g04124\_t001 |  | \| \| |  |  |  |  |  |  |  |  |  |  |
| 1 | Vvi-Vitvi07g02251\_t002 |  | Atr-ERN02066 |  |  |  |  |  |  |  |  |  |  |
| 1 | Vvi-Vitvi07g00518\_t001 |  | \| \| |  |  |  |  |  |  |  |  |  |  |
| 1 | Vvi-Vitvi07g00520\_t001 |  | \| \| |  |  |  |  |  |  |  |  |  |  |
| 1 | Vvi-Vitvi07g00521\_t001 |  | Atr-ERN02062 |  |  |  |  |  |  |  |  |  |  |
| 1 | Vvi-Vitvi07g00522\_t001 |  | Atr-ERN02056 |  |  |  |  |  |  |  |  |  |  |
| 1 | Vvi-Vitvi07g00523\_t001 |  | Atr-ERN02047 |  |  |  |  |  |  |  |  |  |  |
| 1 | Vvi-Vitvi07g00524\_t001 |  | Atr-ERN02042 |  |  |  |  |  |  |  |  |  |  |
| 1 | Vvi-Vitvi07g00525\_t001 |  | Atr-ERN02036 |  |  |  |  |  |  |  |  |  |  |
| 1 | Vvi-Vitvi07g00526\_t001 |  | Atr-ERN02035 |  |  |  |  |  |  |  |  |  |  |
| 1 | Vvi-Vitvi07g00527\_t001 |  | Atr-ERN02034 |  |  |  |  |  |  |  |  |  |  |
| 1 | Vvi-Vitvi07g00529\_t002 |  | \| \| |  |  |  |  |  |  |  |  |  |  |
| 1 | Vvi-Vitvi07g00530\_t001 |  | \| \| |  |  |  |  |  |  |  |  |  |  |
| 1 | Vvi-Vitvi07g04125\_t001 |  | \| \| |  |  |  |  |  |  |  |  |  |  |
| 1 | Vvi-Vitvi07g00531\_t001 |  | Atr-ERN02033 |  |  |  |  |  |  |  |  |  |  |
| 1 | Vvi-Vitvi07g04126\_t001 |  | \| \| |  |  |  |  |  |  |  |  |  |  |
| 1 | Vvi-Vitvi07g00532\_t001 |  | Atr-ERN02032 |  |  |  |  |  |  |  |  |  |  |
| 1 | Vvi-Vitvi07g00533\_t001 |  | Atr-ERN02029 |  |  |  |  |  |  |  |  |  |  |
| 1 | Vvi-Vitvi07g04127\_t001 |  | \| \| |  |  |  |  |  |  |  |  |  |  |
| 1 | Vvi-Vitvi07g04128\_t001 |  | \| \| |  |  |  |  |  |  |  |  |  |  |
| 1 | Vvi-Vitvi07g00534\_t001 |  | Atr-ERN02024 |  |  |  |  |  |  |  |  |  |  |
| 1 | Vvi-Vitvi07g04129\_t001 |  | \| \| |  |  |  |  |  |  |  |  |  |  |
| 1 | Vvi-Vitvi07g00535\_t001 |  | Atr-ERN02020 |  |  |  |  |  |  |  |  |  |  |
| 1 | Vvi-Vitvi07g00537\_t001 |  | Atr-ERN02017 |  |  |  |  |  |  |  |  |  |  |
| 1 | Vvi-Vitvi07g00538\_t001 |  | Atr-ERN02016 |  |  |  |  |  |  |  |  |  |  |
| 1 | Vvi-Vitvi07g02253\_t002 |  | Atr-ERN02013 |  |  |  |  |  |  |  |  |  |  |
| 1 | Vvi-Vitvi07g00539\_t001 |  | Atr-ERN02009 |  |  |  |  |  |  |  |  |  |  |
| 1 | Vvi-Vitvi07g04130\_t001 |  | \| \| |  |  |  |  |  |  |  |  |  |  |
| 1 | Vvi-Vitvi07g04131\_t001 |  | \| \| |  |  |  |  |  |  |  |  |  |  |
| 1 | Vvi-Vitvi07g02254\_t001 |  | \| \| |  |  |  |  |  |  |  |  |  |  |
| 1 | Vvi-Vitvi07g00540\_t001 |  | Atr-ERN02000 |  |  |  |  |  |  |  |  |  |  |
| 0 | Vvi-Vitvi07g00542\_t001 |  |  |  |  |  |  |  |  |  |  |  |  |
| 0 | Vvi-Vitvi07g04132\_t001 |  |  |  |  |  |  |  |  |  |  |  |  |
| 0 | Vvi-Vitvi07g00543\_t001 |  |  |  |  |  |  |  |  |  |  |  |  |
| 0 | Vvi-Vitvi07g00544\_t001 |  |  |  |  |  |  |  |  |  |  |  |  |
| 0 | Vvi-Vitvi07g00545\_t001 |  |  |  |  |  |  |  |  |  |  |  |  |
| 0 | Vvi-Vitvi07g00549\_t001 |  |  |  |  |  |  |  |  |  |  |  |  |
| 0 | Vvi-Vitvi07g04133\_t001 |  |  |  |  |  |  |  |  |  |  |  |  |
| 0 | Vvi-Vitvi07g00550\_t001 |  |  |  |  |  |  |  |  |  |  |  |  |
| 0 | Vvi-Vitvi07g04134\_t001 |  |  |  |  |  |  |  |  |  |  |  |  |
| 0 | Vvi-Vitvi07g04135\_t001 |  |  |  |  |  |  |  |  |  |  |  |  |
| 0 | Vvi-Vitvi07g00551\_t001 |  |  |  |  |  |  |  |  |  |  |  |  |
| 0 | Vvi-Vitvi07g04136\_t001 |  |  |  |  |  |  |  |  |  |  |  |  |
| 0 | Vvi-Vitvi07g04137\_t001 |  |  |  |  |  |  |  |  |  |  |  |  |
| 0 | Vvi-Vitvi07g00554\_t001 |  |  |  |  |  |  |  |  |  |  |  |  |
| 0 | Vvi-Vitvi07g04138\_t001 |  |  |  |  |  |  |  |  |  |  |  |  |
| 0 | Vvi-Vitvi07g04139\_t001 |  |  |  |  |  |  |  |  |  |  |  |  |
| 0 | Vvi-Vitvi07g00559\_t001 |  |  |  |  |  |  |  |  |  |  |  |  |
| 0 | Vvi-Vitvi07g04140\_t001 |  |  |  |  |  |  |  |  |  |  |  |  |
| 0 | Vvi-Vitvi07g04141\_t001 |  |  |  |  |  |  |  |  |  |  |  |  |
| 0 | Vvi-Vitvi07g04142\_t001 |  |  |  |  |  |  |  |  |  |  |  |  |
| 0 | Vvi-Vitvi07g02259\_t001 |  |  |  |  |  |  |  |  |  |  |  |  |
| 0 | Vvi-Vitvi07g04143\_t001 |  |  |  |  |  |  |  |  |  |  |  |  |
| 0 | Vvi-Vitvi07g04144\_t001 |  |  |  |  |  |  |  |  |  |  |  |  |
| 0 | Vvi-Vitvi07g04145\_t001 |  |  |  |  |  |  |  |  |  |  |  |  |
| 0 | Vvi-Vitvi07g02260\_t001 |  |  |  |  |  |  |  |  |  |  |  |  |
| 0 | Vvi-Vitvi07g00561\_t001 |  |  |  |  |  |  |  |  |  |  |  |  |
| 0 | Vvi-Vitvi07g00563\_t001 |  |  |  |  |  |  |  |  |  |  |  |  |
| 0 | Vvi-Vitvi07g00564\_t001 |  |  |  |  |  |  |  |  |  |  |  |  |
| 0 | Vvi-Vitvi07g04146\_t001 |  |  |  |  |  |  |  |  |  |  |  |  |
| 0 | Vvi-Vitvi07g00565\_t001 |  |  |  |  |  |  |  |  |  |  |  |  |
| 0 | Vvi-Vitvi07g04147\_t001 |  |  |  |  |  |  |  |  |  |  |  |  |
| 1 | Vvi-Vitvi07g02261\_t001 |  | Atr-ERM94938 |  |  |  |  |  |  |  |  |  |  |
| 1 | Vvi-Vitvi07g02262\_t001 |  | Atr-ERM94937 |  |  |  |  |  |  |  |  |  |  |
| 1 | Vvi-Vitvi07g04148\_t001 |  | \| \| |  |  |  |  |  |  |  |  |  |  |
| 1 | Vvi-Vitvi07g00567\_t001 |  | \| \| |  |  |  |  |  |  |  |  |  |  |
| 1 | Vvi-Vitvi07g00569\_t001 |  | \| \| |  |  |  |  |  |  |  |  |  |  |
| 1 | Vvi-Vitvi07g00571\_t001 |  | \| \| |  |  |  |  |  |  |  |  |  |  |
| 1 | Vvi-Vitvi07g00572\_t001 |  | \| \| |  |  |  |  |  |  |  |  |  |  |
| 1 | Vvi-Vitvi07g00573\_t001 |  | \| \| |  |  |  |  |  |  |  |  |  |  |
| 1 | Vvi-Vitvi07g00574\_t001 |  | Atr-ERM94927 |  |  |  |  |  |  |  |  |  |  |
| 1 | Vvi-Vitvi07g00575\_t001 |  | \| \| |  |  |  |  |  |  |  |  |  |  |
| 1 | Vvi-Vitvi07g00576\_t001 |  | \| \| |  |  |  |  |  |  |  |  |  |  |
| 1 | Vvi-Vitvi07g00577\_t001 |  | \| \| |  |  |  |  |  |  |  |  |  |  |
| 1 | Vvi-Vitvi07g04149\_t001 |  | \| \| |  |  |  |  |  |  |  |  |  |  |
| 1 | Vvi-Vitvi07g00578\_t001 |  | Atr-ERM94917 |  |  |  |  |  |  |  |  |  |  |
| 1 | Vvi-Vitvi07g00579\_t001 |  | \| \| |  |  |  |  |  |  |  |  |  |  |
| 1 | Vvi-Vitvi07g00580\_t002 |  | \| \| |  |  |  |  |  |  |  |  |  |  |
| 1 | Vvi-Vitvi07g00581\_t001 |  | Atr-ERM94914 |  |  |  |  |  |  |  |  |  |  |
| 1 | Vvi-Vitvi07g00582\_t001 |  | \| \| |  |  |  |  |  |  |  |  |  |  |
| 1 | Vvi-Vitvi07g04150\_t001 |  | \| \| |  |  |  |  |  |  |  |  |  |  |
| 1 | Vvi-Vitvi07g00583\_t001 |  | \| \| |  |  |  |  |  |  |  |  |  |  |
| 1 | Vvi-Vitvi07g00586\_t001 |  | \| \| |  |  |  |  |  |  |  |  |  |  |
| 1 | Vvi-Vitvi07g00587\_t001 |  | \| \| |  |  |  |  |  |  |  |  |  |  |
| 1 | Vvi-Vitvi07g00588\_t001 |  | \| \| |  |  |  |  |  |  |  |  |  |  |
| 1 | Vvi-Vitvi07g04151\_t001 |  | \| \| |  |  |  |  |  |  |  |  |  |  |
| 1 | Vvi-Vitvi07g02062\_t001 |  | Atr-ERM94898 |  |  |  |  |  |  |  |  |  |  |
| 1 | Vvi-Vitvi07g02063\_t001 |  | \| \| |  |  |  |  |  |  |  |  |  |  |
| 1 | Vvi-Vitvi07g04152\_t001 |  | \| \| |  |  |  |  |  |  |  |  |  |  |
| 1 | Vvi-Vitvi07g02064\_t001 |  | \| \| |  |  |  |  |  |  |  |  |  |  |
| 1 | Vvi-Vitvi07g00590\_t001 |  | \| \| |  |  |  |  |  |  |  |  |  |  |
| 1 | Vvi-Vitvi07g02070\_t001 |  | \| \| |  |  |  |  |  |  |  |  |  |  |
| 1 | Vvi-Vitvi07g04153\_t001 |  | \| \| |  |  |  |  |  |  |  |  |  |  |
| 1 | Vvi-Vitvi07g02067\_t001 |  | Atr-ERM94896 |  |  |  |  |  |  |  |  |  |  |
| 1 | Vvi-Vitvi07g02065\_t001 |  | \| \| |  |  |  |  |  |  |  |  |  |  |
| 1 | Vvi-Vitvi07g00591\_t001 |  | \| \| |  |  |  |  |  |  |  |  |  |  |
| 1 | Vvi-Vitvi07g00592\_t001 |  | \| \| |  |  |  |  |  |  |  |  |  |  |
| 1 | Vvi-Vitvi07g02068\_t001 |  | \| \| |  |  |  |  |  |  |  |  |  |  |
| 1 | Vvi-Vitvi07g00594\_t001 |  | Atr-ERM94886 |  |  |  |  |  |  |  |  |  |  |
| 1 | Vvi-Vitvi07g04154\_t001 |  | \| \| |  |  |  |  |  |  |  |  |  |  |
| 1 | Vvi-Vitvi07g02266\_t001 |  | Atr-ERM94884 |  |  |  |  |  |  |  |  |  |  |
| 0 | Vvi-Vitvi07g04155\_t001 |  |  |  |  |  |  |  |  |  |  |  |  |
| 0 | Vvi-Vitvi07g00595\_t002 |  |  |  |  |  |  |  |  |  |  |  |  |
| 0 | Vvi-Vitvi07g04156\_t001 |  |  |  |  |  |  |  |  |  |  |  |  |
| 1 | Vvi-Vitvi07g00597\_t001 |  | Atr-ERN18740 |  |  |  |  |  |  |  |  |  |  |
| 1 | Vvi-Vitvi07g00598\_t001 |  | \| \| |  |  |  |  |  |  |  |  |  |  |
| 1 | Vvi-Vitvi07g04157\_t005 |  | \| \| |  |  |  |  |  |  |  |  |  |  |
| 1 | Vvi-Vitvi07g00601\_t001 |  | \| \| |  |  |  |  |  |  |  |  |  |  |
| 1 | Vvi-Vitvi07g02267\_t001 |  | Atr-ERN18741 |  |  |  |  |  |  |  |  |  |  |
| 1 | Vvi-Vitvi07g00603\_t001 |  | Atr-ERN18748 |  |  |  |  |  |  |  |  |  |  |
| 1 | Vvi-Vitvi07g00604\_t001 |  | Atr-ERN18749 |  |  |  |  |  |  |  |  |  |  |
| 1 | Vvi-Vitvi07g00605\_t001 |  | \| \| |  |  |  |  |  |  |  |  |  |  |
| 1 | Vvi-Vitvi07g02268\_t001 |  | \| \| |  |  |  |  |  |  |  |  |  |  |
| 1 | Vvi-Vitvi07g02269\_t001 |  | \| \| |  |  |  |  |  |  |  |  |  |  |
| 1 | Vvi-Vitvi07g00606\_t001 |  | Atr-ERN18752 |  |  |  |  |  |  |  |  |  |  |
| 1 | Vvi-Vitvi07g00607\_t001 |  | \| \| |  |  |  |  |  |  |  |  |  |  |
| 1 | Vvi-Vitvi07g02270\_t001 |  | \| \| |  |  |  |  |  |  |  |  |  |  |
| 1 | Vvi-Vitvi07g00610\_t001 |  | \| \| |  |  |  |  |  |  |  |  |  |  |
| 1 | Vvi-Vitvi07g00612\_t001 |  | Atr-ERN18758 |  |  |  |  |  |  |  |  |  |  |
| 1 | Vvi-Vitvi07g00613\_t001 |  | Atr-ERN18762 |  |  |  |  |  |  |  |  |  |  |
| 1 | Vvi-Vitvi07g00614\_t001 |  | Atr-ERN18764 |  |  |  |  |  |  |  |  |  |  |
| 1 | Vvi-Vitvi07g00615\_t001 |  | Atr-ERN18766 |  |  |  |  |  |  |  |  |  |  |
| 1 | Vvi-Vitvi07g04158\_t001 |  | \| \| |  |  |  |  |  |  |  |  |  |  |
| 1 | Vvi-Vitvi07g02271\_t001 |  | \| \| |  |  |  |  |  |  |  |  |  |  |
| 1 | Vvi-Vitvi07g04159\_t001 |  | \| \| |  |  |  |  |  |  |  |  |  |  |
| 1 | Vvi-Vitvi07g00618\_t002 |  | Atr-ERN18773 |  |  |  |  |  |  |  |  |  |  |
| 1 | Vvi-Vitvi07g00619\_t001 |  | Atr-ERN18775 |  |  |  |  |  |  |  |  |  |  |
| 1 | Vvi-Vitvi07g00620\_t001 |  | Atr-ERN18779 |  |  |  |  |  |  |  |  |  |  |
| 1 | Vvi-Vitvi07g00621\_t002 |  | Atr-ERN18783 |  |  |  |  |  |  |  |  |  |  |
| 1 | Vvi-Vitvi07g00622\_t002 |  | Atr-ERN18788 |  |  |  |  |  |  |  |  |  |  |
| 1 | Vvi-Vitvi07g00623\_t001 |  | Atr-ERN18791 |  |  |  |  |  |  |  |  |  |  |
| 1 | Vvi-Vitvi07g00624\_t001 |  | Atr-ERN18793 |  |  |  |  |  |  |  |  |  |  |
| 1 | Vvi-Vitvi07g00625\_t001 |  | Atr-ERN18794 |  |  |  |  |  |  |  |  |  |  |
| 1 | Vvi-Vitvi07g04160\_t001 |  | \| \| |  |  |  |  |  |  |  |  |  |  |
| 1 | Vvi-Vitvi07g00626\_t001 |  | Atr-ERN18795 |  |  |  |  |  |  |  |  |  |  |
| 1 | Vvi-Vitvi07g00627\_t001 |  | \| \| |  |  |  |  |  |  |  |  |  |  |
| 1 | Vvi-Vitvi07g00628\_t001 |  | Atr-ERN18798 |  |  |  |  |  |  |  |  |  |  |
| 1 | Vvi-Vitvi07g00629\_t001 |  | \| \| |  |  |  |  |  |  |  |  |  |  |
| 1 | Vvi-Vitvi07g00630\_t002 |  | Atr-ERN18799 |  |  |  |  |  |  |  |  |  |  |
| 1 | Vvi-Vitvi07g00633\_t002 |  | Atr-ERN18812 |  |  |  |  |  |  |  |  |  |  |
| 1 | Vvi-Vitvi07g02274\_t001 |  | \| \| |  |  |  |  |  |  |  |  |  |  |
| 1 | Vvi-Vitvi07g00634\_t001 |  | Atr-ERN18816 |  |  |  |  |  |  |  |  |  |  |
| 1 | Vvi-Vitvi07g04161\_t001 |  | \| \| |  |  |  |  |  |  |  |  |  |  |
| 1 | Vvi-Vitvi07g00635\_t001 |  | Atr-ERN18817 |  |  |  |  |  |  |  |  |  |  |
| 1 | Vvi-Vitvi07g04162\_t001 |  | \| \| |  |  |  |  |  |  |  |  |  |  |
| 1 | Vvi-Vitvi07g00636\_t001 |  | Atr-ERN18825 |  |  |  |  |  |  |  |  |  |  |
| 1 | Vvi-Vitvi07g00637\_t001 |  | Atr-ERN18833 |  |  |  |  |  |  |  |  |  |  |
| 1 | Vvi-Vitvi07g02276\_t001 |  | \| \| |  |  |  |  |  |  |  |  |  |  |
| 2 | Vvi-Vitvi07g00639\_t001 |  | \| \| |  | Atr-ERN18898 |  |  |  |  |  |  |  |  |
| 2 | Vvi-Vitvi07g00640\_t001 |  | \| \| |  | \| \| |  |  |  |  |  |  |  |  |
| 2 | Vvi-Vitvi07g02277\_t001 |  | \| \| |  | \| \| |  |  |  |  |  |  |  |  |
| 2 | Vvi-Vitvi07g00643\_t001 |  | \| \| |  | Atr-ERN18911 |  |  |  |  |  |  |  |  |
| 2 | Vvi-Vitvi07g04163\_t001 |  | \| \| |  | \| \| |  |  |  |  |  |  |  |  |
| 2 | Vvi-Vitvi07g02278\_t001 |  | \| \| |  | \| \| |  |  |  |  |  |  |  |  |
| 2 | Vvi-Vitvi07g02279\_t001 |  | \| \| |  | \| \| |  |  |  |  |  |  |  |  |
| 2 | Vvi-Vitvi07g02280\_t001 |  | \| \| |  | \| \| |  |  |  |  |  |  |  |  |
| 2 | Vvi-Vitvi07g02281\_t001 |  | \| \| |  | \| \| |  |  |  |  |  |  |  |  |
| 2 | Vvi-Vitvi07g02282\_t001 |  | \| \| |  | \| \| |  |  |  |  |  |  |  |  |
| 2 | Vvi-Vitvi07g00644\_t001 |  | \| \| |  | \| \| |  |  |  |  |  |  |  |  |
| 2 | Vvi-Vitvi07g02283\_t001 |  | \| \| |  | \| \| |  |  |  |  |  |  |  |  |
| 2 | Vvi-Vitvi07g02284\_t001 |  | \| \| |  | \| \| |  |  |  |  |  |  |  |  |
| 2 | Vvi-Vitvi07g02285\_t001 |  | \| \| |  | \| \| |  |  |  |  |  |  |  |  |
| 2 | Vvi-Vitvi07g02286\_t001 |  | \| \| |  | \| \| |  |  |  |  |  |  |  |  |
| 2 | Vvi-Vitvi07g00647\_t001 |  | \| \| |  | Atr-ERN18912 |  |  |  |  |  |  |  |  |
| 2 | Vvi-Vitvi07g04164\_t001 |  | \| \| |  | \| \| |  |  |  |  |  |  |  |  |
| 2 | Vvi-Vitvi07g00649\_t001 |  | \| \| |  | Atr-ERN18914 |  |  |  |  |  |  |  |  |
| 2 | Vvi-Vitvi07g00650\_t001 |  | \| \| |  | Atr-ERN18919 |  |  |  |  |  |  |  |  |
| 2 | Vvi-Vitvi07g00652\_t001 |  | \| \| |  | Atr-ERN18921 |  |  |  |  |  |  |  |  |
| 2 | Vvi-Vitvi07g00653\_t001 |  | \| \| |  | Atr-ERN18923 |  |  |  |  |  |  |  |  |
| 2 | Vvi-Vitvi07g00654\_t001 |  | Atr-ERN18838 |  | \| \| |  |  |  |  |  |  |  |  |
| 1 | Vvi-Vitvi07g00656\_t001 |  |  |  | Atr-ERN18925 |  |  |  |  |  |  |  |  |
| 1 | Vvi-Vitvi07g00657\_t001 |  |  |  | \| \| |  |  |  |  |  |  |  |  |
| 1 | Vvi-Vitvi07g00658\_t002 |  |  |  | Atr-ERN18932 |  |  |  |  |  |  |  |  |
| 1 | Vvi-Vitvi07g00659\_t001 |  |  |  | Atr-ERN18938 |  |  |  |  |  |  |  |  |
| 1 | Vvi-Vitvi07g02289\_t001 |  |  |  | Atr-ERN18949 |  |  |  |  |  |  |  |  |
| 1 | Vvi-Vitvi07g02290\_t001 |  |  |  | \| \| |  |  |  |  |  |  |  |  |
| 1 | Vvi-Vitvi07g02291\_t001 |  |  |  | \| \| |  |  |  |  |  |  |  |  |
| 1 | Vvi-Vitvi07g04165\_t001 |  |  |  | \| \| |  |  |  |  |  |  |  |  |
| 1 | Vvi-Vitvi07g00661\_t001 |  |  |  | \| \| |  |  |  |  |  |  |  |  |
| 1 | Vvi-Vitvi07g00664\_t001 |  |  |  | \| \| |  |  |  |  |  |  |  |  |
| 1 | Vvi-Vitvi07g02294\_t001 |  |  |  | \| \| |  |  |  |  |  |  |  |  |
| 1 | Vvi-Vitvi07g00665\_t001 |  |  |  | \| \| |  |  |  |  |  |  |  |  |
| 1 | Vvi-Vitvi07g00666\_t001 |  |  |  | \| \| |  |  |  |  |  |  |  |  |
| 1 | Vvi-Vitvi07g00667\_t001 |  |  |  | Atr-ERN18961 |  |  |  |  |  |  |  |  |
| 1 | Vvi-Vitvi07g00668\_t001 |  |  |  | Atr-ERN18963 |  |  |  |  |  |  |  |  |
| 1 | Vvi-Vitvi07g04166\_t001 |  |  |  | Atr-ERN18964 |  |  |  |  |  |  |  |  |
| 1 | Vvi-Vitvi07g00672\_t001 |  |  |  | \| \| |  |  |  |  |  |  |  |  |
| 1 | Vvi-Vitvi07g00674\_t001 |  |  |  | Atr-ERN18965 |  |  |  |  |  |  |  |  |
| 1 | Vvi-Vitvi07g00675\_t001 |  |  |  | Atr-ERN18966 |  |  |  |  |  |  |  |  |
| 0 | Vvi-Vitvi07g02295\_t001 |  |  |  |  |  |  |  |  |  |  |  |  |
| 0 | Vvi-Vitvi07g00676\_t001 |  |  |  |  |  |  |  |  |  |  |  |  |
| 0 | Vvi-Vitvi07g00677\_t001 |  |  |  |  |  |  |  |  |  |  |  |  |
| 0 | Vvi-Vitvi07g00678\_t001 |  |  |  |  |  |  |  |  |  |  |  |  |
| 0 | Vvi-Vitvi07g00679\_t001 |  |  |  |  |  |  |  |  |  |  |  |  |
| 0 | Vvi-Vitvi07g00680\_t001 |  |  |  |  |  |  |  |  |  |  |  |  |
| 0 | Vvi-Vitvi07g00682\_t001 |  |  |  |  |  |  |  |  |  |  |  |  |
| 0 | Vvi-Vitvi07g00683\_t001 |  |  |  |  |  |  |  |  |  |  |  |  |
| 0 | Vvi-Vitvi07g02298\_t001 |  |  |  |  |  |  |  |  |  |  |  |  |
| 0 | Vvi-Vitvi07g04167\_t001 |  |  |  |  |  |  |  |  |  |  |  |  |
| 0 | Vvi-Vitvi07g00684\_t001 |  |  |  |  |  |  |  |  |  |  |  |  |
| 0 | Vvi-Vitvi07g00685\_t001 |  |  |  |  |  |  |  |  |  |  |  |  |
| 1 | Vvi-Vitvi07g00686\_t001 |  | Atr-ERN15880 |  |  |  |  |  |  |  |  |  |  |
| 1 | Vvi-Vitvi07g04168\_t001 |  | \| \| |  |  |  |  |  |  |  |  |  |  |
| 1 | Vvi-Vitvi07g00687\_t001 |  | Atr-ERN15874 |  |  |  |  |  |  |  |  |  |  |
| 1 | Vvi-Vitvi07g00688\_t001 |  | \| \| |  |  |  |  |  |  |  |  |  |  |
| 1 | Vvi-Vitvi07g00690\_t001 |  | \| \| |  |  |  |  |  |  |  |  |  |  |
| 1 | Vvi-Vitvi07g02300\_t001 |  | \| \| |  |  |  |  |  |  |  |  |  |  |
| 1 | Vvi-Vitvi07g00691\_t001 |  | \| \| |  |  |  |  |  |  |  |  |  |  |
| 1 | Vvi-Vitvi07g00693\_t001 |  | \| \| |  |  |  |  |  |  |  |  |  |  |
| 1 | Vvi-Vitvi07g04169\_t001 |  | \| \| |  |  |  |  |  |  |  |  |  |  |
| 1 | Vvi-Vitvi07g04170\_t001 |  | \| \| |  |  |  |  |  |  |  |  |  |  |
| 1 | Vvi-Vitvi07g00695\_t001 |  | \| \| |  |  |  |  |  |  |  |  |  |  |
| 1 | Vvi-Vitvi07g02301\_t001 |  | \| \| |  |  |  |  |  |  |  |  |  |  |
| 1 | Vvi-Vitvi07g00696\_t001 |  | Atr-ERN15854 |  |  |  |  |  |  |  |  |  |  |
| 1 | Vvi-Vitvi07g00697\_t001 |  | \| \| |  |  |  |  |  |  |  |  |  |  |
| 1 | Vvi-Vitvi07g00698\_t001 |  | \| \| |  |  |  |  |  |  |  |  |  |  |
| 1 | Vvi-Vitvi07g00701\_t001 |  | \| \| |  |  |  |  |  |  |  |  |  |  |
| 1 | Vvi-Vitvi07g04171\_t001 |  | \| \| |  |  |  |  |  |  |  |  |  |  |
| 1 | Vvi-Vitvi07g00703\_t001 |  | \| \| |  |  |  |  |  |  |  |  |  |  |
| 1 | Vvi-Vitvi07g00704\_t001 |  | \| \| |  |  |  |  |  |  |  |  |  |  |
| 1 | Vvi-Vitvi07g00705\_t001 |  | \| \| |  |  |  |  |  |  |  |  |  |  |
| 1 | Vvi-Vitvi07g04172\_t001 |  | \| \| |  |  |  |  |  |  |  |  |  |  |
| 1 | Vvi-Vitvi07g00706\_t001 |  | \| \| |  |  |  |  |  |  |  |  |  |  |
| 1 | Vvi-Vitvi07g00707\_t001 |  | \| \| |  |  |  |  |  |  |  |  |  |  |
| 1 | Vvi-Vitvi07g04173\_t001 |  | \| \| |  |  |  |  |  |  |  |  |  |  |
| 1 | Vvi-Vitvi07g00709\_t001 |  | \| \| |  |  |  |  |  |  |  |  |  |  |
| 1 | Vvi-Vitvi07g00710\_t001 |  | \| \| |  |  |  |  |  |  |  |  |  |  |
| 1 | Vvi-Vitvi07g00711\_t001 |  | \| \| |  |  |  |  |  |  |  |  |  |  |
| 1 | Vvi-Vitvi07g02304\_t001 |  | Atr-ERN15836 |  |  |  |  |  |  |  |  |  |  |
| 1 | Vvi-Vitvi07g02305\_t001 |  | \| \| |  |  |  |  |  |  |  |  |  |  |
| 1 | Vvi-Vitvi07g00716\_t001 |  | \| \| |  |  |  |  |  |  |  |  |  |  |
| 1 | Vvi-Vitvi07g02306\_t001 |  | \| \| |  |  |  |  |  |  |  |  |  |  |
| 1 | Vvi-Vitvi07g04174\_t001 |  | \| \| |  |  |  |  |  |  |  |  |  |  |
| 1 | Vvi-Vitvi07g00719\_t001 |  | \| \| |  |  |  |  |  |  |  |  |  |  |
| 1 | Vvi-Vitvi07g00720\_t001 |  | \| \| |  |  |  |  |  |  |  |  |  |  |
| 1 | Vvi-Vitvi07g00721\_t001 |  | \| \| |  |  |  |  |  |  |  |  |  |  |
| 1 | Vvi-Vitvi07g00722\_t001 |  | \| \| |  |  |  |  |  |  |  |  |  |  |
| 1 | Vvi-Vitvi07g00724\_t001 |  | \| \| |  |  |  |  |  |  |  |  |  |  |
| 1 | Vvi-Vitvi07g00726\_t001 |  | \| \| |  |  |  |  |  |  |  |  |  |  |
| 1 | Vvi-Vitvi07g04175\_t001 |  | \| \| |  |  |  |  |  |  |  |  |  |  |
| 1 | Vvi-Vitvi07g04176\_t001 |  | \| \| |  |  |  |  |  |  |  |  |  |  |
| 1 | Vvi-Vitvi07g00727\_t001 |  | \| \| |  |  |  |  |  |  |  |  |  |  |
| 1 | Vvi-Vitvi07g00730\_t001 |  | Atr-ERN15813 |  |  |  |  |  |  |  |  |  |  |
| 1 | Vvi-Vitvi07g00731\_t001 |  | \| \| |  |  |  |  |  |  |  |  |  |  |
| 1 | Vvi-Vitvi07g00732\_t001 |  | \| \| |  |  |  |  |  |  |  |  |  |  |
| 2 | Vvi-Vitvi07g00733\_t001 |  | \| \| |  | Atr-ERN15790 |  |  |  |  |  |  |  |  |
| 2 | Vvi-Vitvi07g00734\_t001 |  | \| \| |  | \| \| |  |  |  |  |  |  |  |  |
| 2 | Vvi-Vitvi07g00735\_t001 |  | \| \| |  | \| \| |  |  |  |  |  |  |  |  |
| 2 | Vvi-Vitvi07g00736\_t001 |  | \| \| |  | \| \| |  |  |  |  |  |  |  |  |
| 2 | Vvi-Vitvi07g00737\_t001 |  | \| \| |  | Atr-ERN15796 |  |  |  |  |  |  |  |  |
| 2 | Vvi-Vitvi07g04177\_t001 |  | \| \| |  | \| \| |  |  |  |  |  |  |  |  |
| 2 | Vvi-Vitvi07g02309\_t001 |  | Atr-ERN15800 |  | Atr-ERN15800 |  |  |  |  |  |  |  |  |
| 2 | Vvi-Vitvi07g00738\_t001 |  | \| \| |  | \| \| |  |  |  |  |  |  |  |  |
| 2 | Vvi-Vitvi07g00739\_t001 |  | Atr-ERN15788 |  | \| \| |  |  |  |  |  |  |  |  |
| 2 | Vvi-Vitvi07g00740\_t001 |  | Atr-ERN15787 |  | \| \| |  |  |  |  |  |  |  |  |
| 2 | Vvi-Vitvi07g00741\_t001 |  | \| \| |  | Atr-ERN15810 |  |  |  |  |  |  |  |  |
| 2 | Vvi-Vitvi07g00742\_t001 |  | \| \| |  | \| \| |  |  |  |  |  |  |  |  |
| 2 | Vvi-Vitvi07g04178\_t001 |  | \| \| |  | \| \| |  |  |  |  |  |  |  |  |
| 2 | Vvi-Vitvi07g04179\_t001 |  | \| \| |  | \| \| |  |  |  |  |  |  |  |  |
| 2 | Vvi-Vitvi07g04180\_t001 |  | \| \| |  | \| \| |  |  |  |  |  |  |  |  |
| 2 | Vvi-Vitvi07g00743\_t001 |  | \| \| |  | Atr-ERN15812 |  |  |  |  |  |  |  |  |
| 2 | Vvi-Vitvi07g00744\_t001 |  | \| \| |  | Atr-ERN15813 |  |  |  |  |  |  |  |  |
| 1 | Vvi-Vitvi07g00745\_t001 |  | \| \| |  |  |  |  |  |  |  |  |  |  |
| 1 | Vvi-Vitvi07g04181\_t001 |  | \| \| |  |  |  |  |  |  |  |  |  |  |
| 1 | Vvi-Vitvi07g04182\_t001 |  | \| \| |  |  |  |  |  |  |  |  |  |  |
| 1 | Vvi-Vitvi07g04183\_t001 |  | \| \| |  |  |  |  |  |  |  |  |  |  |
| 1 | Vvi-Vitvi07g04184\_t001 |  | \| \| |  |  |  |  |  |  |  |  |  |  |
| 1 | Vvi-Vitvi07g04185\_t001 |  | \| \| |  |  |  |  |  |  |  |  |  |  |
| 1 | Vvi-Vitvi07g04186\_t001 |  | \| \| |  |  |  |  |  |  |  |  |  |  |
| 1 | Vvi-Vitvi07g04187\_t001 |  | \| \| |  |  |  |  |  |  |  |  |  |  |
| 1 | Vvi-Vitvi07g04188\_t001 |  | \| \| |  |  |  |  |  |  |  |  |  |  |
| 1 | Vvi-Vitvi07g04189\_t001 |  | \| \| |  |  |  |  |  |  |  |  |  |  |
| 1 | Vvi-Vitvi07g04190\_t001 |  | \| \| |  |  |  |  |  |  |  |  |  |  |
| 1 | Vvi-Vitvi07g04191\_t001 |  | \| \| |  |  |  |  |  |  |  |  |  |  |
| 1 | Vvi-Vitvi07g00762\_t001 |  | \| \| |  |  |  |  |  |  |  |  |  |  |
| 1 | Vvi-Vitvi07g02313\_t001 |  | Atr-ERN15786 |  |  |  |  |  |  |  |  |  |  |
| 0 | Vvi-Vitvi07g00765\_t001 |  |  |  |  |  |  |  |  |  |  |  |  |
| 0 | Vvi-Vitvi07g02315\_t001 |  |  |  |  |  |  |  |  |  |  |  |  |
| 0 | Vvi-Vitvi07g00767\_t001 |  |  |  |  |  |  |  |  |  |  |  |  |
| 0 | Vvi-Vitvi07g02317\_t001 |  |  |  |  |  |  |  |  |  |  |  |  |
| 0 | Vvi-Vitvi07g02318\_t001 |  |  |  |  |  |  |  |  |  |  |  |  |
| 0 | Vvi-Vitvi07g04192\_t001 |  |  |  |  |  |  |  |  |  |  |  |  |
| 0 | Vvi-Vitvi07g04193\_t001 |  |  |  |  |  |  |  |  |  |  |  |  |
| 0 | Vvi-Vitvi07g04194\_t001 |  |  |  |  |  |  |  |  |  |  |  |  |
| 0 | Vvi-Vitvi07g04195\_t001 |  |  |  |  |  |  |  |  |  |  |  |  |
| 0 | Vvi-Vitvi07g04196\_t001 |  |  |  |  |  |  |  |  |  |  |  |  |
| 0 | Vvi-Vitvi07g04197\_t001 |  |  |  |  |  |  |  |  |  |  |  |  |
| 0 | Vvi-Vitvi07g02320\_t001 |  |  |  |  |  |  |  |  |  |  |  |  |
| 0 | Vvi-Vitvi07g00773\_t001 |  |  |  |  |  |  |  |  |  |  |  |  |
| 0 | Vvi-Vitvi07g02321\_t001 |  |  |  |  |  |  |  |  |  |  |  |  |
| 0 | Vvi-Vitvi07g04198\_t001 |  |  |  |  |  |  |  |  |  |  |  |  |
| 0 | Vvi-Vitvi07g02323\_t001 |  |  |  |  |  |  |  |  |  |  |  |  |
| 1 | Vvi-Vitvi07g02326\_t001 |  | Atr-ERN18904 |  |  |  |  |  |  |  |  |  |  |
| 1 | Vvi-Vitvi07g00780\_t001 |  | \| \| |  |  |  |  |  |  |  |  |  |  |
| 1 | Vvi-Vitvi07g04199\_t001 |  | \| \| |  |  |  |  |  |  |  |  |  |  |
| 1 | Vvi-Vitvi07g00781\_t001 |  | \| \| |  |  |  |  |  |  |  |  |  |  |
| 1 | Vvi-Vitvi07g02330\_t001 |  | \| \| |  |  |  |  |  |  |  |  |  |  |
| 1 | Vvi-Vitvi07g04200\_t001 |  | \| \| |  |  |  |  |  |  |  |  |  |  |
| 1 | Vvi-Vitvi07g04201\_t001 |  | \| \| |  |  |  |  |  |  |  |  |  |  |
| 1 | Vvi-Vitvi07g04202\_t001 |  | \| \| |  |  |  |  |  |  |  |  |  |  |
| 1 | Vvi-Vitvi07g02334\_t001 |  | \| \| |  |  |  |  |  |  |  |  |  |  |
| 1 | Vvi-Vitvi07g00786\_t001 |  | \| \| |  |  |  |  |  |  |  |  |  |  |
| 1 | Vvi-Vitvi07g00787\_t001 |  | \| \| |  |  |  |  |  |  |  |  |  |  |
| 1 | Vvi-Vitvi07g00790\_t001 |  | \| \| |  |  |  |  |  |  |  |  |  |  |
| 1 | Vvi-Vitvi07g02337\_t001 |  | \| \| |  |  |  |  |  |  |  |  |  |  |
| 1 | Vvi-Vitvi07g04203\_t001 |  | \| \| |  |  |  |  |  |  |  |  |  |  |
| 1 | Vvi-Vitvi07g04204\_t001 |  | \| \| |  |  |  |  |  |  |  |  |  |  |
| 1 | Vvi-Vitvi07g02342\_t001 |  | \| \| |  |  |  |  |  |  |  |  |  |  |
| 1 | Vvi-Vitvi07g00792\_t001 |  | \| \| |  |  |  |  |  |  |  |  |  |  |
| 1 | Vvi-Vitvi07g00795\_t001 |  | \| \| |  |  |  |  |  |  |  |  |  |  |
| 1 | Vvi-Vitvi07g00796\_t001 |  | \| \| |  |  |  |  |  |  |  |  |  |  |
| 1 | Vvi-Vitvi07g00801\_t003 |  | \| \| |  |  |  |  |  |  |  |  |  |  |
| 1 | Vvi-Vitvi07g02345\_t001 |  | \| \| |  |  |  |  |  |  |  |  |  |  |
| 1 | Vvi-Vitvi07g04205\_t001 |  | \| \| |  |  |  |  |  |  |  |  |  |  |
| 1 | Vvi-Vitvi07g00803\_t001 |  | \| \| |  |  |  |  |  |  |  |  |  |  |
| 1 | Vvi-Vitvi07g00806\_t001 |  | Atr-ERN18878 |  |  |  |  |  |  |  |  |  |  |
| 1 | Vvi-Vitvi07g00807\_t001 |  | Atr-ERN18877 |  |  |  |  |  |  |  |  |  |  |
| 1 | Vvi-Vitvi07g04206\_t001 |  | \| \| |  |  |  |  |  |  |  |  |  |  |
| 1 | Vvi-Vitvi07g00809\_t001 |  | \| \| |  |  |  |  |  |  |  |  |  |  |
| 1 | Vvi-Vitvi07g04207\_t001 |  | \| \| |  |  |  |  |  |  |  |  |  |  |
| 1 | Vvi-Vitvi07g04208\_t001 |  | \| \| |  |  |  |  |  |  |  |  |  |  |
| 1 | Vvi-Vitvi07g00810\_t001 |  | \| \| |  |  |  |  |  |  |  |  |  |  |
| 1 | Vvi-Vitvi07g00812\_t001 |  | \| \| |  |  |  |  |  |  |  |  |  |  |
| 1 | Vvi-Vitvi07g04209\_t001 |  | \| \| |  |  |  |  |  |  |  |  |  |  |
| 1 | Vvi-Vitvi07g04210\_t001 |  | \| \| |  |  |  |  |  |  |  |  |  |  |
| 1 | Vvi-Vitvi07g00814\_t001 |  | \| \| |  |  |  |  |  |  |  |  |  |  |
| 1 | Vvi-Vitvi07g02346\_t001 |  | \| \| |  |  |  |  |  |  |  |  |  |  |
| 1 | Vvi-Vitvi07g02347\_t001 |  | \| \| |  |  |  |  |  |  |  |  |  |  |
| 1 | Vvi-Vitvi07g00822\_t001 |  | \| \| |  |  |  |  |  |  |  |  |  |  |
| 1 | Vvi-Vitvi07g00824\_t001 |  | Atr-ERN18876 |  |  |  |  |  |  |  |  |  |  |
| 1 | Vvi-Vitvi07g00825\_t001 |  | Atr-ERN18875 |  |  |  |  |  |  |  |  |  |  |
| 1 | Vvi-Vitvi07g04211\_t001 |  | \| \| |  |  |  |  |  |  |  |  |  |  |
| 1 | Vvi-Vitvi07g00827\_t001 |  | \| \| |  |  |  |  |  |  |  |  |  |  |
| 1 | Vvi-Vitvi07g04212\_t001 |  | \| \| |  |  |  |  |  |  |  |  |  |  |
| 1 | Vvi-Vitvi07g00830\_t001 |  | Atr-ERN18864 |  |  |  |  |  |  |  |  |  |  |
| 0 | Vvi-Vitvi07g00831\_t001 |  |  |  |  |  |  |  |  |  |  |  |  |
| 0 | Vvi-Vitvi07g04213\_t001 |  |  |  |  |  |  |  |  |  |  |  |  |
| 0 | Vvi-Vitvi07g04214\_t001 |  |  |  |  |  |  |  |  |  |  |  |  |
| 0 | Vvi-Vitvi07g00837\_t001 |  |  |  |  |  |  |  |  |  |  |  |  |
| 0 | Vvi-Vitvi07g04215\_t001 |  |  |  |  |  |  |  |  |  |  |  |  |
| 0 | Vvi-Vitvi07g04216\_t001 |  |  |  |  |  |  |  |  |  |  |  |  |
| 0 | Vvi-Vitvi07g04217\_t001 |  |  |  |  |  |  |  |  |  |  |  |  |
| 0 | Vvi-Vitvi07g04218\_t001 |  |  |  |  |  |  |  |  |  |  |  |  |
| 0 | Vvi-Vitvi07g00846\_t001 |  |  |  |  |  |  |  |  |  |  |  |  |
| 0 | Vvi-Vitvi07g04219\_t001 |  |  |  |  |  |  |  |  |  |  |  |  |
| 0 | Vvi-Vitvi07g04220\_t001 |  |  |  |  |  |  |  |  |  |  |  |  |
| 0 | Vvi-Vitvi07g04221\_t001 |  |  |  |  |  |  |  |  |  |  |  |  |
| 0 | Vvi-Vitvi07g02355\_t001 |  |  |  |  |  |  |  |  |  |  |  |  |
| 0 | Vvi-Vitvi07g00850\_t001 |  |  |  |  |  |  |  |  |  |  |  |  |
| 0 | Vvi-Vitvi07g00853\_t001 |  |  |  |  |  |  |  |  |  |  |  |  |
| 0 | Vvi-Vitvi07g04222\_t001 |  |  |  |  |  |  |  |  |  |  |  |  |
| 0 | Vvi-Vitvi07g04223\_t001 |  |  |  |  |  |  |  |  |  |  |  |  |
| 0 | Vvi-Vitvi07g00857\_t001 |  |  |  |  |  |  |  |  |  |  |  |  |
| 0 | Vvi-Vitvi07g02357\_t001 |  |  |  |  |  |  |  |  |  |  |  |  |
| 0 | Vvi-Vitvi07g04224\_t001 |  |  |  |  |  |  |  |  |  |  |  |  |
| 0 | Vvi-Vitvi07g04225\_t001 |  |  |  |  |  |  |  |  |  |  |  |  |
| 0 | Vvi-Vitvi07g00867\_t001 |  |  |  |  |  |  |  |  |  |  |  |  |
| 0 | Vvi-Vitvi07g00869\_t001 |  |  |  |  |  |  |  |  |  |  |  |  |
| 0 | Vvi-Vitvi07g04226\_t001 |  |  |  |  |  |  |  |  |  |  |  |  |
| 0 | Vvi-Vitvi07g04227\_t001 |  |  |  |  |  |  |  |  |  |  |  |  |
| 0 | Vvi-Vitvi07g04228\_t001 |  |  |  |  |  |  |  |  |  |  |  |  |
| 0 | Vvi-Vitvi07g00871\_t001 |  |  |  |  |  |  |  |  |  |  |  |  |
| 0 | Vvi-Vitvi07g00874\_t001 |  |  |  |  |  |  |  |  |  |  |  |  |
| 0 | Vvi-Vitvi07g04229\_t001 |  |  |  |  |  |  |  |  |  |  |  |  |
| 0 | Vvi-Vitvi07g04230\_t001 |  |  |  |  |  |  |  |  |  |  |  |  |
| 0 | Vvi-Vitvi07g04231\_t001 |  |  |  |  |  |  |  |  |  |  |  |  |
| 0 | Vvi-Vitvi07g02360\_t001 |  |  |  |  |  |  |  |  |  |  |  |  |
| 0 | Vvi-Vitvi07g04232\_t001 |  |  |  |  |  |  |  |  |  |  |  |  |
| 0 | Vvi-Vitvi07g04233\_t001 |  |  |  |  |  |  |  |  |  |  |  |  |
| 0 | Vvi-Vitvi07g02362\_t001 |  |  |  |  |  |  |  |  |  |  |  |  |
| 0 | Vvi-Vitvi07g00879\_t001 |  |  |  |  |  |  |  |  |  |  |  |  |
| 0 | Vvi-Vitvi07g02363\_t001 |  |  |  |  |  |  |  |  |  |  |  |  |
| 0 | Vvi-Vitvi07g04234\_t001 |  |  |  |  |  |  |  |  |  |  |  |  |
| 0 | Vvi-Vitvi07g00883\_t001 |  |  |  |  |  |  |  |  |  |  |  |  |
| 0 | Vvi-Vitvi07g00884\_t001 |  |  |  |  |  |  |  |  |  |  |  |  |
| 0 | Vvi-Vitvi07g02364\_t001 |  |  |  |  |  |  |  |  |  |  |  |  |
| 0 | Vvi-Vitvi07g00887\_t001 |  |  |  |  |  |  |  |  |  |  |  |  |
| 0 | Vvi-Vitvi07g04235\_t001 |  |  |  |  |  |  |  |  |  |  |  |  |
| 0 | Vvi-Vitvi07g00889\_t001 |  |  |  |  |  |  |  |  |  |  |  |  |
| 0 | Vvi-Vitvi07g00890\_t001 |  |  |  |  |  |  |  |  |  |  |  |  |
| 0 | Vvi-Vitvi07g00892\_t001 |  |  |  |  |  |  |  |  |  |  |  |  |
| 0 | Vvi-Vitvi07g00894\_t001 |  |  |  |  |  |  |  |  |  |  |  |  |
| 0 | Vvi-Vitvi07g04236\_t001 |  |  |  |  |  |  |  |  |  |  |  |  |
| 0 | Vvi-Vitvi07g04237\_t001 |  |  |  |  |  |  |  |  |  |  |  |  |
| 0 | Vvi-Vitvi07g00899\_t001 |  |  |  |  |  |  |  |  |  |  |  |  |
| 0 | Vvi-Vitvi07g04238\_t001 |  |  |  |  |  |  |  |  |  |  |  |  |
| 0 | Vvi-Vitvi07g00903\_t001 |  |  |  |  |  |  |  |  |  |  |  |  |
| 0 | Vvi-Vitvi07g04239\_t001 |  |  |  |  |  |  |  |  |  |  |  |  |
| 0 | Vvi-Vitvi07g00905\_t001 |  |  |  |  |  |  |  |  |  |  |  |  |
| 0 | Vvi-Vitvi07g00906\_t001 |  |  |  |  |  |  |  |  |  |  |  |  |
| 0 | Vvi-Vitvi07g00907\_t001 |  |  |  |  |  |  |  |  |  |  |  |  |
| 0 | Vvi-Vitvi07g02371\_t001 |  |  |  |  |  |  |  |  |  |  |  |  |
| 0 | Vvi-Vitvi07g00908\_t001 |  |  |  |  |  |  |  |  |  |  |  |  |
| 0 | Vvi-Vitvi07g00910\_t001 |  |  |  |  |  |  |  |  |  |  |  |  |
| 0 | Vvi-Vitvi07g04240\_t001 |  |  |  |  |  |  |  |  |  |  |  |  |
| 0 | Vvi-Vitvi07g00912\_t001 |  |  |  |  |  |  |  |  |  |  |  |  |
| 0 | Vvi-Vitvi07g02374\_t001 |  |  |  |  |  |  |  |  |  |  |  |  |
| 0 | Vvi-Vitvi07g04241\_t001 |  |  |  |  |  |  |  |  |  |  |  |  |
| 0 | Vvi-Vitvi07g04242\_t001 |  |  |  |  |  |  |  |  |  |  |  |  |
| 0 | Vvi-Vitvi07g00914\_t002 |  |  |  |  |  |  |  |  |  |  |  |  |
| 0 | Vvi-Vitvi07g04243\_t001 |  |  |  |  |  |  |  |  |  |  |  |  |
| 0 | Vvi-Vitvi07g00916\_t001 |  |  |  |  |  |  |  |  |  |  |  |  |
| 0 | Vvi-Vitvi07g00918\_t001 |  |  |  |  |  |  |  |  |  |  |  |  |
| 0 | Vvi-Vitvi07g00919\_t001 |  |  |  |  |  |  |  |  |  |  |  |  |
| 0 | Vvi-Vitvi07g04244\_t001 |  |  |  |  |  |  |  |  |  |  |  |  |
| 0 | Vvi-Vitvi07g00921\_t001 |  |  |  |  |  |  |  |  |  |  |  |  |
| 0 | Vvi-Vitvi07g00923\_t001 |  |  |  |  |  |  |  |  |  |  |  |  |
| 0 | Vvi-Vitvi07g00924\_t001 |  |  |  |  |  |  |  |  |  |  |  |  |
| 0 | Vvi-Vitvi07g04245\_t001 |  |  |  |  |  |  |  |  |  |  |  |  |
| 0 | Vvi-Vitvi07g00930\_t002 |  |  |  |  |  |  |  |  |  |  |  |  |
| 0 | Vvi-Vitvi07g04246\_t001 |  |  |  |  |  |  |  |  |  |  |  |  |
| 0 | Vvi-Vitvi07g04247\_t001 |  |  |  |  |  |  |  |  |  |  |  |  |
| 0 | Vvi-Vitvi07g04248\_t001 |  |  |  |  |  |  |  |  |  |  |  |  |
| 0 | Vvi-Vitvi07g04249\_t001 |  |  |  |  |  |  |  |  |  |  |  |  |
| 0 | Vvi-Vitvi07g04250\_t001 |  |  |  |  |  |  |  |  |  |  |  |  |
| 0 | Vvi-Vitvi07g02071\_t001 |  |  |  |  |  |  |  |  |  |  |  |  |
| 0 | Vvi-Vitvi07g00933\_t001 |  |  |  |  |  |  |  |  |  |  |  |  |
| 0 | Vvi-Vitvi07g00934\_t001 |  |  |  |  |  |  |  |  |  |  |  |  |
| 0 | Vvi-Vitvi07g00936\_t001 |  |  |  |  |  |  |  |  |  |  |  |  |
| 0 | Vvi-Vitvi07g04251\_t001 |  |  |  |  |  |  |  |  |  |  |  |  |
| 0 | Vvi-Vitvi07g04252\_t001 |  |  |  |  |  |  |  |  |  |  |  |  |
| 0 | Vvi-Vitvi07g00939\_t001 |  |  |  |  |  |  |  |  |  |  |  |  |
| 0 | Vvi-Vitvi07g02382\_t001 |  |  |  |  |  |  |  |  |  |  |  |  |
| 0 | Vvi-Vitvi07g00940\_t001 |  |  |  |  |  |  |  |  |  |  |  |  |
| 0 | Vvi-Vitvi07g00941\_t001 |  |  |  |  |  |  |  |  |  |  |  |  |
| 0 | Vvi-Vitvi07g00943\_t002 |  |  |  |  |  |  |  |  |  |  |  |  |
| 0 | Vvi-Vitvi07g04253\_t001 |  |  |  |  |  |  |  |  |  |  |  |  |
| 0 | Vvi-Vitvi07g00949\_t001 |  |  |  |  |  |  |  |  |  |  |  |  |
| 0 | Vvi-Vitvi07g02734\_t001 |  |  |  |  |  |  |  |  |  |  |  |  |
| 0 | Vvi-Vitvi07g04254\_t001 |  |  |  |  |  |  |  |  |  |  |  |  |
| 0 | Vvi-Vitvi07g04255\_t001 |  |  |  |  |  |  |  |  |  |  |  |  |
| 0 | Vvi-Vitvi07g00955\_t001 |  |  |  |  |  |  |  |  |  |  |  |  |
| 0 | Vvi-Vitvi07g04256\_t001 |  |  |  |  |  |  |  |  |  |  |  |  |
| 0 | Vvi-Vitvi07g00960\_t001 |  |  |  |  |  |  |  |  |  |  |  |  |
| 0 | Vvi-Vitvi07g02730\_t001 |  |  |  |  |  |  |  |  |  |  |  |  |
| 0 | Vvi-Vitvi07g04257\_t001 |  |  |  |  |  |  |  |  |  |  |  |  |
| 0 | Vvi-Vitvi07g04258\_t001 |  |  |  |  |  |  |  |  |  |  |  |  |
| 0 | Vvi-Vitvi07g02386\_t001 |  |  |  |  |  |  |  |  |  |  |  |  |
| 0 | Vvi-Vitvi07g04259\_t001 |  |  |  |  |  |  |  |  |  |  |  |  |
| 0 | Vvi-Vitvi07g02389\_t001 |  |  |  |  |  |  |  |  |  |  |  |  |
| 0 | Vvi-Vitvi07g04260\_t001 |  |  |  |  |  |  |  |  |  |  |  |  |
| 0 | Vvi-Vitvi07g04261\_t001 |  |  |  |  |  |  |  |  |  |  |  |  |
| 0 | Vvi-Vitvi07g04262\_t001 |  |  |  |  |  |  |  |  |  |  |  |  |
| 0 | Vvi-Vitvi07g00974\_t001 |  |  |  |  |  |  |  |  |  |  |  |  |
| 0 | Vvi-Vitvi07g00978\_t001 |  |  |  |  |  |  |  |  |  |  |  |  |
| 0 | Vvi-Vitvi07g04263\_t001 |  |  |  |  |  |  |  |  |  |  |  |  |
| 0 | Vvi-Vitvi07g04264\_t001 |  |  |  |  |  |  |  |  |  |  |  |  |
| 0 | Vvi-Vitvi07g00980\_t001 |  |  |  |  |  |  |  |  |  |  |  |  |
| 0 | Vvi-Vitvi07g04265\_t001 |  |  |  |  |  |  |  |  |  |  |  |  |
| 0 | Vvi-Vitvi07g04266\_t001 |  |  |  |  |  |  |  |  |  |  |  |  |
| 0 | Vvi-Vitvi07g00983\_t001 |  |  |  |  |  |  |  |  |  |  |  |  |
| 0 | Vvi-Vitvi07g04267\_t001 |  |  |  |  |  |  |  |  |  |  |  |  |
| 0 | Vvi-Vitvi07g00984\_t001 |  |  |  |  |  |  |  |  |  |  |  |  |
| 0 | Vvi-Vitvi07g04268\_t001 |  |  |  |  |  |  |  |  |  |  |  |  |
| 0 | Vvi-Vitvi07g04269\_t001 |  |  |  |  |  |  |  |  |  |  |  |  |
| 0 | Vvi-Vitvi07g00988\_t001 |  |  |  |  |  |  |  |  |  |  |  |  |
| 0 | Vvi-Vitvi07g02397\_t001 |  |  |  |  |  |  |  |  |  |  |  |  |
| 0 | Vvi-Vitvi07g04270\_t001 |  |  |  |  |  |  |  |  |  |  |  |  |
| 0 | Vvi-Vitvi07g04271\_t001 |  |  |  |  |  |  |  |  |  |  |  |  |
| 0 | Vvi-Vitvi07g01004\_t001 |  |  |  |  |  |  |  |  |  |  |  |  |
| 0 | Vvi-Vitvi07g02399\_t001 |  |  |  |  |  |  |  |  |  |  |  |  |
| 0 | Vvi-Vitvi07g02402\_t001 |  |  |  |  |  |  |  |  |  |  |  |  |
| 0 | Vvi-Vitvi07g02403\_t001 |  |  |  |  |  |  |  |  |  |  |  |  |
| 0 | Vvi-Vitvi07g02405\_t001 |  |  |  |  |  |  |  |  |  |  |  |  |
| 0 | Vvi-Vitvi07g04272\_t001 |  |  |  |  |  |  |  |  |  |  |  |  |
| 0 | Vvi-Vitvi07g01017\_t001 |  |  |  |  |  |  |  |  |  |  |  |  |
| 0 | Vvi-Vitvi07g02410\_t001 |  |  |  |  |  |  |  |  |  |  |  |  |
| 0 | Vvi-Vitvi07g02412\_t001 |  |  |  |  |  |  |  |  |  |  |  |  |
| 0 | Vvi-Vitvi07g01026\_t001 |  |  |  |  |  |  |  |  |  |  |  |  |
| 0 | Vvi-Vitvi07g04273\_t001 |  |  |  |  |  |  |  |  |  |  |  |  |
| 0 | Vvi-Vitvi07g04274\_t001 |  |  |  |  |  |  |  |  |  |  |  |  |
| 0 | Vvi-Vitvi07g04275\_t001 |  |  |  |  |  |  |  |  |  |  |  |  |
| 0 | Vvi-Vitvi07g02414\_t001 |  |  |  |  |  |  |  |  |  |  |  |  |
| 0 | Vvi-Vitvi07g02415\_t001 |  |  |  |  |  |  |  |  |  |  |  |  |
| 0 | Vvi-Vitvi07g04276\_t001 |  |  |  |  |  |  |  |  |  |  |  |  |
| 0 | Vvi-Vitvi07g04277\_t001 |  |  |  |  |  |  |  |  |  |  |  |  |
| 0 | Vvi-Vitvi07g01032\_t001 |  |  |  |  |  |  |  |  |  |  |  |  |
| 0 | Vvi-Vitvi07g01033\_t001 |  |  |  |  |  |  |  |  |  |  |  |  |
| 0 | Vvi-Vitvi07g01038\_t001 |  |  |  |  |  |  |  |  |  |  |  |  |
| 0 | Vvi-Vitvi07g04278\_t001 |  |  |  |  |  |  |  |  |  |  |  |  |
| 0 | Vvi-Vitvi07g01039\_t001 |  |  |  |  |  |  |  |  |  |  |  |  |
| 0 | Vvi-Vitvi07g01041\_t001 |  |  |  |  |  |  |  |  |  |  |  |  |
| 0 | Vvi-Vitvi07g01042\_t001 |  |  |  |  |  |  |  |  |  |  |  |  |
| 0 | Vvi-Vitvi07g04279\_t001 |  |  |  |  |  |  |  |  |  |  |  |  |
| 0 | Vvi-Vitvi07g01045\_t001 |  |  |  |  |  |  |  |  |  |  |  |  |
| 0 | Vvi-Vitvi07g04280\_t001 |  |  |  |  |  |  |  |  |  |  |  |  |
| 0 | Vvi-Vitvi07g02418\_t001 |  |  |  |  |  |  |  |  |  |  |  |  |
| 0 | Vvi-Vitvi07g01050\_t001 |  |  |  |  |  |  |  |  |  |  |  |  |
| 0 | Vvi-Vitvi07g04281\_t001 |  |  |  |  |  |  |  |  |  |  |  |  |
| 0 | Vvi-Vitvi07g04282\_t001 |  |  |  |  |  |  |  |  |  |  |  |  |
| 0 | Vvi-Vitvi07g04283\_t001 |  |  |  |  |  |  |  |  |  |  |  |  |
| 0 | Vvi-Vitvi07g04284\_t001 |  |  |  |  |  |  |  |  |  |  |  |  |
| 0 | Vvi-Vitvi07g04285\_t001 |  |  |  |  |  |  |  |  |  |  |  |  |
| 0 | Vvi-Vitvi07g02878\_t001 |  |  |  |  |  |  |  |  |  |  |  |  |
| 0 | Vvi-Vitvi07g02881\_t001 |  |  |  |  |  |  |  |  |  |  |  |  |
| 0 | Vvi-Vitvi07g04286\_t001 |  |  |  |  |  |  |  |  |  |  |  |  |
| 0 | Vvi-Vitvi07g02882\_t001 |  |  |  |  |  |  |  |  |  |  |  |  |
| 0 | Vvi-Vitvi07g02883\_t001 |  |  |  |  |  |  |  |  |  |  |  |  |
| 0 | Vvi-Vitvi07g02885\_t001 |  |  |  |  |  |  |  |  |  |  |  |  |
| 0 | Vvi-Vitvi07g02886\_t001 |  |  |  |  |  |  |  |  |  |  |  |  |
| 0 | Vvi-Vitvi07g04287\_t001 |  |  |  |  |  |  |  |  |  |  |  |  |
| 0 | Vvi-Vitvi07g02888\_t001 |  |  |  |  |  |  |  |  |  |  |  |  |
| 0 | Vvi-Vitvi07g04288\_t001 |  |  |  |  |  |  |  |  |  |  |  |  |
| 0 | Vvi-Vitvi07g04289\_t001 |  |  |  |  |  |  |  |  |  |  |  |  |
| 0 | Vvi-Vitvi07g04290\_t001 |  |  |  |  |  |  |  |  |  |  |  |  |
| 0 | Vvi-Vitvi07g04291\_t001 |  |  |  |  |  |  |  |  |  |  |  |  |
| 0 | Vvi-Vitvi07g02745\_t001 |  |  |  |  |  |  |  |  |  |  |  |  |
| 0 | Vvi-Vitvi07g02751\_t001 |  |  |  |  |  |  |  |  |  |  |  |  |
| 0 | Vvi-Vitvi07g02752\_t001 |  |  |  |  |  |  |  |  |  |  |  |  |
| 0 | Vvi-Vitvi07g02753\_t001 |  |  |  |  |  |  |  |  |  |  |  |  |
| 0 | Vvi-Vitvi07g02755\_t001 |  |  |  |  |  |  |  |  |  |  |  |  |
| 0 | Vvi-Vitvi07g04292\_t001 |  |  |  |  |  |  |  |  |  |  |  |  |
| 0 | Vvi-Vitvi07g02756\_t001 |  |  |  |  |  |  |  |  |  |  |  |  |
| 0 | Vvi-Vitvi07g02757\_t001 |  |  |  |  |  |  |  |  |  |  |  |  |
| 0 | Vvi-Vitvi07g02759\_t001 |  |  |  |  |  |  |  |  |  |  |  |  |
| 0 | Vvi-Vitvi07g04293\_t001 |  |  |  |  |  |  |  |  |  |  |  |  |
| 0 | Vvi-Vitvi07g04294\_t001 |  |  |  |  |  |  |  |  |  |  |  |  |
| 0 | Vvi-Vitvi07g02767\_t001 |  |  |  |  |  |  |  |  |  |  |  |  |
| 0 | Vvi-Vitvi07g02768\_t002 |  |  |  |  |  |  |  |  |  |  |  |  |
| 0 | Vvi-Vitvi07g04295\_t001 |  |  |  |  |  |  |  |  |  |  |  |  |
| 0 | Vvi-Vitvi07g02776\_t001 |  |  |  |  |  |  |  |  |  |  |  |  |
| 0 | Vvi-Vitvi07g02787\_t001 |  |  |  |  |  |  |  |  |  |  |  |  |
| 0 | Vvi-Vitvi07g02789\_t001 |  |  |  |  |  |  |  |  |  |  |  |  |
| 0 | Vvi-Vitvi07g02791\_t001 |  |  |  |  |  |  |  |  |  |  |  |  |
| 0 | Vvi-Vitvi07g04298\_t001 |  |  |  |  |  |  |  |  |  |  |  |  |
| 0 | Vvi-Vitvi07g04299\_t001 |  |  |  |  |  |  |  |  |  |  |  |  |
| 0 | Vvi-Vitvi07g04300\_t001 |  |  |  |  |  |  |  |  |  |  |  |  |
| 0 | Vvi-Vitvi07g01054\_t001 |  |  |  |  |  |  |  |  |  |  |  |  |
| 0 | Vvi-Vitvi07g04301\_t001 |  |  |  |  |  |  |  |  |  |  |  |  |
| 0 | Vvi-Vitvi07g01055\_t001 |  |  |  |  |  |  |  |  |  |  |  |  |
| 0 | Vvi-Vitvi07g04302\_t002 |  |  |  |  |  |  |  |  |  |  |  |  |
| 0 | Vvi-Vitvi07g01057\_t001 |  |  |  |  |  |  |  |  |  |  |  |  |
| 0 | Vvi-Vitvi07g04303\_t001 |  |  |  |  |  |  |  |  |  |  |  |  |
| 0 | Vvi-Vitvi07g04304\_t001 |  |  |  |  |  |  |  |  |  |  |  |  |
| 0 | Vvi-Vitvi07g04305\_t001 |  |  |  |  |  |  |  |  |  |  |  |  |
| 0 | Vvi-Vitvi07g04306\_t002 |  |  |  |  |  |  |  |  |  |  |  |  |
| 0 | Vvi-Vitvi07g01088\_t001 |  |  |  |  |  |  |  |  |  |  |  |  |
| 0 | Vvi-Vitvi07g04307\_t002 |  |  |  |  |  |  |  |  |  |  |  |  |
| 0 | Vvi-Vitvi07g01086\_t001 |  |  |  |  |  |  |  |  |  |  |  |  |
| 0 | Vvi-Vitvi07g02433\_t001 |  |  |  |  |  |  |  |  |  |  |  |  |
| 0 | Vvi-Vitvi07g02432\_t001 |  |  |  |  |  |  |  |  |  |  |  |  |
| 0 | Vvi-Vitvi07g02431\_t001 |  |  |  |  |  |  |  |  |  |  |  |  |
| 0 | Vvi-Vitvi07g04308\_t001 |  |  |  |  |  |  |  |  |  |  |  |  |
| 0 | Vvi-Vitvi07g04309\_t001 |  |  |  |  |  |  |  |  |  |  |  |  |
| 1 | Vvi-Vitvi07g01078\_t001 |  | Atr-ERN10058 |  |  |  |  |  |  |  |  |  |  |
| 1 | Vvi-Vitvi07g04310\_t001 |  | \| \| |  |  |  |  |  |  |  |  |  |  |
| 1 | Vvi-Vitvi07g04311\_t001 |  | \| \| |  |  |  |  |  |  |  |  |  |  |
| 1 | Vvi-Vitvi07g04312\_t001 |  | \| \| |  |  |  |  |  |  |  |  |  |  |
| 1 | Vvi-Vitvi07g04313\_t001 |  | \| \| |  |  |  |  |  |  |  |  |  |  |
| 1 | Vvi-Vitvi07g04314\_t001 |  | \| \| |  |  |  |  |  |  |  |  |  |  |
| 1 | Vvi-Vitvi07g01113\_t001 |  | \| \| |  |  |  |  |  |  |  |  |  |  |
| 1 | Vvi-Vitvi07g04315\_t001 |  | \| \| |  |  |  |  |  |  |  |  |  |  |
| 1 | Vvi-Vitvi07g01114\_t001 |  | Atr-ERN10065 |  |  |  |  |  |  |  |  |  |  |
| 1 | Vvi-Vitvi07g04316\_t001 |  | \| \| |  |  |  |  |  |  |  |  |  |  |
| 1 | Vvi-Vitvi07g01119\_t001 |  | \| \| |  |  |  |  |  |  |  |  |  |  |
| 1 | Vvi-Vitvi07g02441\_t001 |  | \| \| |  |  |  |  |  |  |  |  |  |  |
| 1 | Vvi-Vitvi07g01120\_t001 |  | Atr-ERN10066 |  |  |  |  |  |  |  |  |  |  |
| 1 | Vvi-Vitvi07g01124\_t001 |  | \| \| |  |  |  |  |  |  |  |  |  |  |
| 1 | Vvi-Vitvi07g04317\_t001 |  | \| \| |  |  |  |  |  |  |  |  |  |  |
| 1 | Vvi-Vitvi07g02902\_t001 |  | \| \| |  |  |  |  |  |  |  |  |  |  |
| 1 | Vvi-Vitvi07g04318\_t001 |  | \| \| |  |  |  |  |  |  |  |  |  |  |
| 1 | Vvi-Vitvi07g04319\_t001 |  | \| \| |  |  |  |  |  |  |  |  |  |  |
| 1 | Vvi-Vitvi07g02955\_t001 |  | \| \| |  |  |  |  |  |  |  |  |  |  |
| 1 | Vvi-Vitvi07g04320\_t001 |  | \| \| |  |  |  |  |  |  |  |  |  |  |
| 1 | Vvi-Vitvi07g04321\_t001 |  | \| \| |  |  |  |  |  |  |  |  |  |  |
| 1 | Vvi-Vitvi07g04322\_t001 |  | \| \| |  |  |  |  |  |  |  |  |  |  |
| 1 | Vvi-Vitvi07g02951\_t001 |  | \| \| |  |  |  |  |  |  |  |  |  |  |
| 1 | Vvi-Vitvi07g04323\_t001 |  | \| \| |  |  |  |  |  |  |  |  |  |  |
| 1 | Vvi-Vitvi07g02949\_t001 |  | \| \| |  |  |  |  |  |  |  |  |  |  |
| 1 | Vvi-Vitvi07g02948\_t001 |  | \| \| |  |  |  |  |  |  |  |  |  |  |
| 1 | Vvi-Vitvi07g04324\_t001 |  | \| \| |  |  |  |  |  |  |  |  |  |  |
| 1 | Vvi-Vitvi07g04325\_t001 |  | \| \| |  |  |  |  |  |  |  |  |  |  |
| 1 | Vvi-Vitvi07g02940\_t001 |  | \| \| |  |  |  |  |  |  |  |  |  |  |
| 1 | Vvi-Vitvi07g04326\_t001 |  | \| \| |  |  |  |  |  |  |  |  |  |  |
| 1 | Vvi-Vitvi07g04327\_t001 |  | \| \| |  |  |  |  |  |  |  |  |  |  |
| 1 | Vvi-Vitvi07g01110\_t003 |  | Atr-ERN10085 |  |  |  |  |  |  |  |  |  |  |
| 1 | Vvi-Vitvi07g04328\_t001 |  | \| \| |  |  |  |  |  |  |  |  |  |  |
| 1 | Vvi-Vitvi07g01109\_t001 |  | \| \| |  |  |  |  |  |  |  |  |  |  |
| 1 | Vvi-Vitvi07g01108\_t001 |  | Atr-ERN10086 |  |  |  |  |  |  |  |  |  |  |
| 1 | Vvi-Vitvi07g04329\_t001 |  | Atr-ERN10089 |  |  |  |  |  |  |  |  |  |  |
| 0 | Vvi-Vitvi07g01104\_t001 |  |  |  |  |  |  |  |  |  |  |  |  |
| 0 | Vvi-Vitvi07g04330\_t001 |  |  |  |  |  |  |  |  |  |  |  |  |
| 0 | Vvi-Vitvi07g04331\_t001 |  |  |  |  |  |  |  |  |  |  |  |  |
| 0 | Vvi-Vitvi07g04332\_t001 |  |  |  |  |  |  |  |  |  |  |  |  |
| 0 | Vvi-Vitvi07g04333\_t001 |  |  |  |  |  |  |  |  |  |  |  |  |
| 0 | Vvi-Vitvi07g04334\_t001 |  |  |  |  |  |  |  |  |  |  |  |  |
| 0 | Vvi-Vitvi07g04335\_t001 |  |  |  |  |  |  |  |  |  |  |  |  |
| 0 | Vvi-Vitvi07g04336\_t001 |  |  |  |  |  |  |  |  |  |  |  |  |
| 0 | Vvi-Vitvi07g04337\_t001 |  |  |  |  |  |  |  |  |  |  |  |  |
| 0 | Vvi-Vitvi07g01097\_t001 |  |  |  |  |  |  |  |  |  |  |  |  |
| 0 | Vvi-Vitvi07g04338\_t001 |  |  |  |  |  |  |  |  |  |  |  |  |
| 0 | Vvi-Vitvi07g04339\_t001 |  |  |  |  |  |  |  |  |  |  |  |  |
| 0 | Vvi-Vitvi07g04340\_t001 |  |  |  |  |  |  |  |  |  |  |  |  |
| 0 | Vvi-Vitvi07g04341\_t001 |  |  |  |  |  |  |  |  |  |  |  |  |
| 0 | Vvi-Vitvi07g04342\_t001 |  |  |  |  |  |  |  |  |  |  |  |  |
| 0 | Vvi-Vitvi07g01095\_t001 |  |  |  |  |  |  |  |  |  |  |  |  |
| 0 | Vvi-Vitvi07g04343\_t001 |  |  |  |  |  |  |  |  |  |  |  |  |
| 0 | Vvi-Vitvi07g04344\_t001 |  |  |  |  |  |  |  |  |  |  |  |  |
| 0 | Vvi-Vitvi07g02804\_t001 |  |  |  |  |  |  |  |  |  |  |  |  |
| 0 | Vvi-Vitvi07g04345\_t001 |  |  |  |  |  |  |  |  |  |  |  |  |
| 0 | Vvi-Vitvi07g01059\_t002 |  |  |  |  |  |  |  |  |  |  |  |  |
| 0 | Vvi-Vitvi07g01060\_t001 |  |  |  |  |  |  |  |  |  |  |  |  |
| 0 | Vvi-Vitvi07g01061\_t001 |  |  |  |  |  |  |  |  |  |  |  |  |
| 0 | Vvi-Vitvi07g01064\_t001 |  |  |  |  |  |  |  |  |  |  |  |  |
| 0 | Vvi-Vitvi07g01066\_t001 |  |  |  |  |  |  |  |  |  |  |  |  |
| 0 | Vvi-Vitvi07g04346\_t001 |  |  |  |  |  |  |  |  |  |  |  |  |
| 0 | Vvi-Vitvi07g04347\_t001 |  |  |  |  |  |  |  |  |  |  |  |  |
| 0 | Vvi-Vitvi07g04348\_t001 |  |  |  |  |  |  |  |  |  |  |  |  |
| 0 | Vvi-Vitvi07g04349\_t001 |  |  |  |  |  |  |  |  |  |  |  |  |
| 0 | Vvi-Vitvi07g04350\_t001 |  |  |  |  |  |  |  |  |  |  |  |  |
| 0 | Vvi-Vitvi07g04351\_t001 |  |  |  |  |  |  |  |  |  |  |  |  |
| 0 | Vvi-Vitvi07g04352\_t001 |  |  |  |  |  |  |  |  |  |  |  |  |
| 0 | Vvi-Vitvi07g02420\_t001 |  |  |  |  |  |  |  |  |  |  |  |  |
| 0 | Vvi-Vitvi07g02421\_t001 |  |  |  |  |  |  |  |  |  |  |  |  |
| 0 | Vvi-Vitvi07g02422\_t001 |  |  |  |  |  |  |  |  |  |  |  |  |
| 0 | Vvi-Vitvi07g04353\_t001 |  |  |  |  |  |  |  |  |  |  |  |  |
| 0 | Vvi-Vitvi07g04354\_t001 |  |  |  |  |  |  |  |  |  |  |  |  |
| 0 | Vvi-Vitvi07g04355\_t001 |  |  |  |  |  |  |  |  |  |  |  |  |
| 0 | Vvi-Vitvi07g04356\_t001 |  |  |  |  |  |  |  |  |  |  |  |  |
| 0 | Vvi-Vitvi07g04357\_t001 |  |  |  |  |  |  |  |  |  |  |  |  |
| 0 | Vvi-Vitvi07g04358\_t001 |  |  |  |  |  |  |  |  |  |  |  |  |
| 0 | Vvi-Vitvi07g04359\_t001 |  |  |  |  |  |  |  |  |  |  |  |  |
| 0 | Vvi-Vitvi07g04360\_t001 |  |  |  |  |  |  |  |  |  |  |  |  |
| 0 | Vvi-Vitvi07g01071\_t001 |  |  |  |  |  |  |  |  |  |  |  |  |
| 0 | Vvi-Vitvi07g02425\_t003 |  |  |  |  |  |  |  |  |  |  |  |  |
| 0 | Vvi-Vitvi07g01075\_t001 |  |  |  |  |  |  |  |  |  |  |  |  |
| 0 | Vvi-Vitvi07g04363\_t001 |  |  |  |  |  |  |  |  |  |  |  |  |
| 0 | Vvi-Vitvi07g02428\_t001 |  |  |  |  |  |  |  |  |  |  |  |  |
| 0 | Vvi-Vitvi07g04364\_t001 |  |  |  |  |  |  |  |  |  |  |  |  |
| 0 | Vvi-Vitvi07g02430\_t001 |  |  |  |  |  |  |  |  |  |  |  |  |
| 0 | Vvi-Vitvi07g04365\_t001 |  |  |  |  |  |  |  |  |  |  |  |  |
| 0 | Vvi-Vitvi07g04366\_t001 |  |  |  |  |  |  |  |  |  |  |  |  |
| 0 | Vvi-Vitvi07g04367\_t001 |  |  |  |  |  |  |  |  |  |  |  |  |
| 0 | Vvi-Vitvi07g04368\_t001 |  |  |  |  |  |  |  |  |  |  |  |  |
| 0 | Vvi-Vitvi07g04369\_t001 |  |  |  |  |  |  |  |  |  |  |  |  |
| 0 | Vvi-Vitvi07g04370\_t001 |  |  |  |  |  |  |  |  |  |  |  |  |
| 0 | Vvi-Vitvi07g04371\_t001 |  |  |  |  |  |  |  |  |  |  |  |  |
| 0 | Vvi-Vitvi07g04372\_t001 |  |  |  |  |  |  |  |  |  |  |  |  |
| 0 | Vvi-Vitvi07g04373\_t001 |  |  |  |  |  |  |  |  |  |  |  |  |
| 0 | Vvi-Vitvi07g02812\_t001 |  |  |  |  |  |  |  |  |  |  |  |  |
| 0 | Vvi-Vitvi07g04374\_t001 |  |  |  |  |  |  |  |  |  |  |  |  |
| 0 | Vvi-Vitvi07g02831\_t001 |  |  |  |  |  |  |  |  |  |  |  |  |
| 0 | Vvi-Vitvi07g04375\_t001 |  |  |  |  |  |  |  |  |  |  |  |  |
| 0 | Vvi-Vitvi07g02817\_t001 |  |  |  |  |  |  |  |  |  |  |  |  |
| 0 | Vvi-Vitvi07g02820\_t001 |  |  |  |  |  |  |  |  |  |  |  |  |
| 0 | Vvi-Vitvi07g04376\_t001 |  |  |  |  |  |  |  |  |  |  |  |  |
| 0 | Vvi-Vitvi07g04377\_t001 |  |  |  |  |  |  |  |  |  |  |  |  |
| 0 | Vvi-Vitvi07g02826\_t001 |  |  |  |  |  |  |  |  |  |  |  |  |
| 0 | Vvi-Vitvi07g04378\_t001 |  |  |  |  |  |  |  |  |  |  |  |  |
| 0 | Vvi-Vitvi07g04379\_t001 |  |  |  |  |  |  |  |  |  |  |  |  |
| 0 | Vvi-Vitvi07g04380\_t001 |  |  |  |  |  |  |  |  |  |  |  |  |
| 0 | Vvi-Vitvi07g04381\_t001 |  |  |  |  |  |  |  |  |  |  |  |  |
| 0 | Vvi-Vitvi07g02832\_t001 |  |  |  |  |  |  |  |  |  |  |  |  |
| 0 | Vvi-Vitvi07g04382\_t001 |  |  |  |  |  |  |  |  |  |  |  |  |
| 0 | Vvi-Vitvi07g04383\_t001 |  |  |  |  |  |  |  |  |  |  |  |  |
| 0 | Vvi-Vitvi07g04384\_t001 |  |  |  |  |  |  |  |  |  |  |  |  |
| 0 | Vvi-Vitvi07g04385\_t001 |  |  |  |  |  |  |  |  |  |  |  |  |
| 0 | Vvi-Vitvi07g04386\_t001 |  |  |  |  |  |  |  |  |  |  |  |  |
| 0 | Vvi-Vitvi07g04387\_t001 |  |  |  |  |  |  |  |  |  |  |  |  |
| 0 | Vvi-Vitvi07g04388\_t001 |  |  |  |  |  |  |  |  |  |  |  |  |
| 0 | Vvi-Vitvi07g04389\_t001 |  |  |  |  |  |  |  |  |  |  |  |  |
| 0 | Vvi-Vitvi07g04390\_t001 |  |  |  |  |  |  |  |  |  |  |  |  |
| 0 | Vvi-Vitvi07g04391\_t001 |  |  |  |  |  |  |  |  |  |  |  |  |
| 0 | Vvi-Vitvi07g04392\_t001 |  |  |  |  |  |  |  |  |  |  |  |  |
| 0 | Vvi-Vitvi07g01133\_t001 |  |  |  |  |  |  |  |  |  |  |  |  |
| 0 | Vvi-Vitvi07g04393\_t001 |  |  |  |  |  |  |  |  |  |  |  |  |
| 0 | Vvi-Vitvi07g04394\_t001 |  |  |  |  |  |  |  |  |  |  |  |  |
| 0 | Vvi-Vitvi07g01130\_t001 |  |  |  |  |  |  |  |  |  |  |  |  |
| 0 | Vvi-Vitvi07g01129\_t001 |  |  |  |  |  |  |  |  |  |  |  |  |
| 0 | Vvi-Vitvi07g04395\_t001 |  |  |  |  |  |  |  |  |  |  |  |  |
| 0 | Vvi-Vitvi07g01128\_t001 |  |  |  |  |  |  |  |  |  |  |  |  |
| 0 | Vvi-Vitvi07g04396\_t001 |  |  |  |  |  |  |  |  |  |  |  |  |
| 0 | Vvi-Vitvi07g04397\_t002 |  |  |  |  |  |  |  |  |  |  |  |  |
| 0 | Vvi-Vitvi07g01140\_t001 |  |  |  |  |  |  |  |  |  |  |  |  |
| 0 | Vvi-Vitvi07g04398\_t001 |  |  |  |  |  |  |  |  |  |  |  |  |
| 0 | Vvi-Vitvi07g01139\_t001 |  |  |  |  |  |  |  |  |  |  |  |  |
| 0 | Vvi-Vitvi07g04399\_t001 |  |  |  |  |  |  |  |  |  |  |  |  |
| 0 | Vvi-Vitvi07g01137\_t002 |  |  |  |  |  |  |  |  |  |  |  |  |
| 0 | Vvi-Vitvi07g04400\_t001 |  |  |  |  |  |  |  |  |  |  |  |  |
| 0 | Vvi-Vitvi07g04401\_t001 |  |  |  |  |  |  |  |  |  |  |  |  |
| 0 | Vvi-Vitvi07g04402\_t001 |  |  |  |  |  |  |  |  |  |  |  |  |
| 0 | Vvi-Vitvi07g04403\_t001 |  |  |  |  |  |  |  |  |  |  |  |  |
| 0 | Vvi-Vitvi07g01165\_t001 |  |  |  |  |  |  |  |  |  |  |  |  |
| 0 | Vvi-Vitvi07g01164\_t001 |  |  |  |  |  |  |  |  |  |  |  |  |
| 0 | Vvi-Vitvi07g01163\_t001 |  |  |  |  |  |  |  |  |  |  |  |  |
| 0 | Vvi-Vitvi07g01162\_t002 |  |  |  |  |  |  |  |  |  |  |  |  |
| 0 | Vvi-Vitvi07g01160\_t001 |  |  |  |  |  |  |  |  |  |  |  |  |
| 0 | Vvi-Vitvi07g01159\_t001 |  |  |  |  |  |  |  |  |  |  |  |  |
| 0 | Vvi-Vitvi07g04404\_t001 |  |  |  |  |  |  |  |  |  |  |  |  |
| 0 | Vvi-Vitvi07g04405\_t001 |  |  |  |  |  |  |  |  |  |  |  |  |
| 0 | Vvi-Vitvi07g04406\_t001 |  |  |  |  |  |  |  |  |  |  |  |  |
| 0 | Vvi-Vitvi07g04407\_t001 |  |  |  |  |  |  |  |  |  |  |  |  |
| 0 | Vvi-Vitvi07g01151\_t001 |  |  |  |  |  |  |  |  |  |  |  |  |
| 0 | Vvi-Vitvi07g04408\_t001 |  |  |  |  |  |  |  |  |  |  |  |  |
| 0 | Vvi-Vitvi07g04409\_t001 |  |  |  |  |  |  |  |  |  |  |  |  |
| 0 | Vvi-Vitvi07g02447\_t001 |  |  |  |  |  |  |  |  |  |  |  |  |
| 0 | Vvi-Vitvi07g04410\_t001 |  |  |  |  |  |  |  |  |  |  |  |  |
| 0 | Vvi-Vitvi07g01147\_t001 |  |  |  |  |  |  |  |  |  |  |  |  |
| 0 | Vvi-Vitvi07g02463\_t001 |  |  |  |  |  |  |  |  |  |  |  |  |
| 0 | Vvi-Vitvi07g04411\_t001 |  |  |  |  |  |  |  |  |  |  |  |  |
| 0 | Vvi-Vitvi07g02450\_t001 |  |  |  |  |  |  |  |  |  |  |  |  |
| 0 | Vvi-Vitvi07g02465\_t001 |  |  |  |  |  |  |  |  |  |  |  |  |
| 0 | Vvi-Vitvi07g04412\_t001 |  |  |  |  |  |  |  |  |  |  |  |  |
| 0 | Vvi-Vitvi07g04413\_t001 |  |  |  |  |  |  |  |  |  |  |  |  |
| 0 | Vvi-Vitvi07g04414\_t001 |  |  |  |  |  |  |  |  |  |  |  |  |
| 0 | Vvi-Vitvi07g03153\_t001 |  |  |  |  |  |  |  |  |  |  |  |  |
| 0 | Vvi-Vitvi07g01178\_t001 |  |  |  |  |  |  |  |  |  |  |  |  |
| 0 | Vvi-Vitvi07g01179\_t001 |  |  |  |  |  |  |  |  |  |  |  |  |
| 0 | Vvi-Vitvi07g02467\_t002 |  |  |  |  |  |  |  |  |  |  |  |  |
| 0 | Vvi-Vitvi07g01180\_t001 |  |  |  |  |  |  |  |  |  |  |  |  |
| 0 | Vvi-Vitvi07g01181\_t001 |  |  |  |  |  |  |  |  |  |  |  |  |
| 0 | Vvi-Vitvi07g01182\_t001 |  |  |  |  |  |  |  |  |  |  |  |  |
| 0 | Vvi-Vitvi07g01183\_t001 |  |  |  |  |  |  |  |  |  |  |  |  |
| 0 | Vvi-Vitvi07g04415\_t001 |  |  |  |  |  |  |  |  |  |  |  |  |
| 0 | Vvi-Vitvi07g04416\_t001 |  |  |  |  |  |  |  |  |  |  |  |  |
| 0 | Vvi-Vitvi07g04417\_t001 |  |  |  |  |  |  |  |  |  |  |  |  |
| 0 | Vvi-Vitvi07g04418\_t001 |  |  |  |  |  |  |  |  |  |  |  |  |
| 0 | Vvi-Vitvi07g04419\_t001 |  |  |  |  |  |  |  |  |  |  |  |  |
| 0 | Vvi-Vitvi07g04420\_t001 |  |  |  |  |  |  |  |  |  |  |  |  |
| 0 | Vvi-Vitvi07g04421\_t001 |  |  |  |  |  |  |  |  |  |  |  |  |
| 0 | Vvi-Vitvi07g04422\_t001 |  |  |  |  |  |  |  |  |  |  |  |  |
| 0 | Vvi-Vitvi07g04423\_t001 |  |  |  |  |  |  |  |  |  |  |  |  |
| 0 | Vvi-Vitvi07g04424\_t001 |  |  |  |  |  |  |  |  |  |  |  |  |
| 0 | Vvi-Vitvi07g04425\_t001 |  |  |  |  |  |  |  |  |  |  |  |  |
| 0 | Vvi-Vitvi07g04426\_t001 |  |  |  |  |  |  |  |  |  |  |  |  |
| 0 | Vvi-Vitvi07g04427\_t001 |  |  |  |  |  |  |  |  |  |  |  |  |
| 0 | Vvi-Vitvi07g04428\_t001 |  |  |  |  |  |  |  |  |  |  |  |  |
| 0 | Vvi-Vitvi07g04429\_t001 |  |  |  |  |  |  |  |  |  |  |  |  |
| 0 | Vvi-Vitvi07g04430\_t001 |  |  |  |  |  |  |  |  |  |  |  |  |
| 0 | Vvi-Vitvi07g02962\_t001 |  |  |  |  |  |  |  |  |  |  |  |  |
| 0 | Vvi-Vitvi07g02963\_t001 |  |  |  |  |  |  |  |  |  |  |  |  |
| 0 | Vvi-Vitvi07g04431\_t001 |  |  |  |  |  |  |  |  |  |  |  |  |
| 0 | Vvi-Vitvi07g02969\_t001 |  |  |  |  |  |  |  |  |  |  |  |  |
| 0 | Vvi-Vitvi07g02970\_t001 |  |  |  |  |  |  |  |  |  |  |  |  |
| 0 | Vvi-Vitvi07g02971\_t001 |  |  |  |  |  |  |  |  |  |  |  |  |
| 0 | Vvi-Vitvi07g04432\_t001 |  |  |  |  |  |  |  |  |  |  |  |  |
| 0 | Vvi-Vitvi07g04433\_t001 |  |  |  |  |  |  |  |  |  |  |  |  |
| 0 | Vvi-Vitvi07g04434\_t001 |  |  |  |  |  |  |  |  |  |  |  |  |
| 0 | Vvi-Vitvi07g04435\_t001 |  |  |  |  |  |  |  |  |  |  |  |  |
| 0 | Vvi-Vitvi07g04436\_t001 |  |  |  |  |  |  |  |  |  |  |  |  |
| 0 | Vvi-Vitvi07g04437\_t001 |  |  |  |  |  |  |  |  |  |  |  |  |
| 0 | Vvi-Vitvi07g04438\_t001 |  |  |  |  |  |  |  |  |  |  |  |  |
| 0 | Vvi-Vitvi07g04439\_t001 |  |  |  |  |  |  |  |  |  |  |  |  |
| 0 | Vvi-Vitvi07g04440\_t001 |  |  |  |  |  |  |  |  |  |  |  |  |
| 0 | Vvi-Vitvi07g03001\_t001 |  |  |  |  |  |  |  |  |  |  |  |  |
| 0 | Vvi-Vitvi07g04441\_t001 |  |  |  |  |  |  |  |  |  |  |  |  |
| 0 | Vvi-Vitvi07g02461\_t001 |  |  |  |  |  |  |  |  |  |  |  |  |
| 0 | Vvi-Vitvi07g04443\_t001 |  |  |  |  |  |  |  |  |  |  |  |  |
| 0 | Vvi-Vitvi07g04444\_t001 |  |  |  |  |  |  |  |  |  |  |  |  |
| 0 | Vvi-Vitvi07g04445\_t001 |  |  |  |  |  |  |  |  |  |  |  |  |
| 0 | Vvi-Vitvi07g04446\_t002 |  |  |  |  |  |  |  |  |  |  |  |  |
| 0 | Vvi-Vitvi07g04447\_t001 |  |  |  |  |  |  |  |  |  |  |  |  |
| 0 | Vvi-Vitvi07g01198\_t001 |  |  |  |  |  |  |  |  |  |  |  |  |
| 0 | Vvi-Vitvi07g01199\_t001 |  |  |  |  |  |  |  |  |  |  |  |  |
| 0 | Vvi-Vitvi07g02480\_t002 |  |  |  |  |  |  |  |  |  |  |  |  |
| 0 | Vvi-Vitvi07g01200\_t001 |  |  |  |  |  |  |  |  |  |  |  |  |
| 0 | Vvi-Vitvi07g02481\_t001 |  |  |  |  |  |  |  |  |  |  |  |  |
| 0 | Vvi-Vitvi07g04448\_t001 |  |  |  |  |  |  |  |  |  |  |  |  |
| 0 | Vvi-Vitvi07g01202\_t001 |  |  |  |  |  |  |  |  |  |  |  |  |
| 0 | Vvi-Vitvi07g02482\_t002 |  |  |  |  |  |  |  |  |  |  |  |  |
| 0 | Vvi-Vitvi07g04449\_t001 |  |  |  |  |  |  |  |  |  |  |  |  |
| 0 | Vvi-Vitvi07g04450\_t001 |  |  |  |  |  |  |  |  |  |  |  |  |
| 0 | Vvi-Vitvi07g04451\_t001 |  |  |  |  |  |  |  |  |  |  |  |  |
| 0 | Vvi-Vitvi07g01203\_t005 |  |  |  |  |  |  |  |  |  |  |  |  |
| 0 | Vvi-Vitvi07g04452\_t001 |  |  |  |  |  |  |  |  |  |  |  |  |
| 0 | Vvi-Vitvi07g02484\_t001 |  |  |  |  |  |  |  |  |  |  |  |  |
| 0 | Vvi-Vitvi07g02485\_t001 |  |  |  |  |  |  |  |  |  |  |  |  |
| 0 | Vvi-Vitvi07g04453\_t001 |  |  |  |  |  |  |  |  |  |  |  |  |
| 0 | Vvi-Vitvi07g04454\_t001 |  |  |  |  |  |  |  |  |  |  |  |  |
| 0 | Vvi-Vitvi07g04455\_t001 |  |  |  |  |  |  |  |  |  |  |  |  |
| 0 | Vvi-Vitvi07g01205\_t001 |  |  |  |  |  |  |  |  |  |  |  |  |
| 0 | Vvi-Vitvi07g01209\_t001 |  |  |  |  |  |  |  |  |  |  |  |  |
| 0 | Vvi-Vitvi07g02487\_t001 |  |  |  |  |  |  |  |  |  |  |  |  |
| 0 | Vvi-Vitvi07g01211\_t001 |  |  |  |  |  |  |  |  |  |  |  |  |
| 0 | Vvi-Vitvi07g04456\_t001 |  |  |  |  |  |  |  |  |  |  |  |  |
| 0 | Vvi-Vitvi07g02488\_t001 |  |  |  |  |  |  |  |  |  |  |  |  |
| 0 | Vvi-Vitvi07g04457\_t001 |  |  |  |  |  |  |  |  |  |  |  |  |
| 0 | Vvi-Vitvi07g04458\_t001 |  |  |  |  |  |  |  |  |  |  |  |  |
| 0 | Vvi-Vitvi07g04459\_t001 |  |  |  |  |  |  |  |  |  |  |  |  |
| 0 | Vvi-Vitvi07g04460\_t001 |  |  |  |  |  |  |  |  |  |  |  |  |
| 0 | Vvi-Vitvi07g04461\_t001 |  |  |  |  |  |  |  |  |  |  |  |  |
| 0 | Vvi-Vitvi07g04462\_t001 |  |  |  |  |  |  |  |  |  |  |  |  |
| 0 | Vvi-Vitvi07g02491\_t002 |  |  |  |  |  |  |  |  |  |  |  |  |
| 0 | Vvi-Vitvi07g04463\_t002 |  |  |  |  |  |  |  |  |  |  |  |  |
| 1 | Vvi-Vitvi07g01215\_t001 |  | Atr-ERN20165 |  |  |  |  |  |  |  |  |  |  |
| 1 | Vvi-Vitvi07g04464\_t001 |  | \| \| |  |  |  |  |  |  |  |  |  |  |
| 1 | Vvi-Vitvi07g01217\_t001 |  | \| \| |  |  |  |  |  |  |  |  |  |  |
| 1 | Vvi-Vitvi07g01219\_t001 |  | Atr-ERN20170 |  |  |  |  |  |  |  |  |  |  |
| 1 | Vvi-Vitvi07g04465\_t001 |  | \| \| |  |  |  |  |  |  |  |  |  |  |
| 1 | Vvi-Vitvi07g01221\_t001 |  | \| \| |  |  |  |  |  |  |  |  |  |  |
| 1 | Vvi-Vitvi07g04466\_t001 |  | \| \| |  |  |  |  |  |  |  |  |  |  |
| 1 | Vvi-Vitvi07g04467\_t001 |  | \| \| |  |  |  |  |  |  |  |  |  |  |
| 1 | Vvi-Vitvi07g04468\_t001 |  | \| \| |  |  |  |  |  |  |  |  |  |  |
| 1 | Vvi-Vitvi07g04469\_t001 |  | \| \| |  |  |  |  |  |  |  |  |  |  |
| 1 | Vvi-Vitvi07g04470\_t001 |  | \| \| |  |  |  |  |  |  |  |  |  |  |
| 1 | Vvi-Vitvi07g01225\_t001 |  | \| \| |  |  |  |  |  |  |  |  |  |  |
| 1 | Vvi-Vitvi07g04471\_t001 |  | \| \| |  |  |  |  |  |  |  |  |  |  |
| 1 | Vvi-Vitvi07g03118\_t001 |  | \| \| |  |  |  |  |  |  |  |  |  |  |
| 1 | Vvi-Vitvi07g02995\_t001 |  | \| \| |  |  |  |  |  |  |  |  |  |  |
| 1 | Vvi-Vitvi07g04472\_t001 |  | \| \| |  |  |  |  |  |  |  |  |  |  |
| 1 | Vvi-Vitvi07g04473\_t001 |  | \| \| |  |  |  |  |  |  |  |  |  |  |
| 1 | Vvi-Vitvi07g04474\_t001 |  | \| \| |  |  |  |  |  |  |  |  |  |  |
| 1 | Vvi-Vitvi07g04475\_t001 |  | \| \| |  |  |  |  |  |  |  |  |  |  |
| 1 | Vvi-Vitvi07g04476\_t001 |  | \| \| |  |  |  |  |  |  |  |  |  |  |
| 1 | Vvi-Vitvi07g04477\_t001 |  | \| \| |  |  |  |  |  |  |  |  |  |  |
| 1 | Vvi-Vitvi07g04478\_t001 |  | \| \| |  |  |  |  |  |  |  |  |  |  |
| 1 | Vvi-Vitvi07g01231\_t002 |  | \| \| |  |  |  |  |  |  |  |  |  |  |
| 1 | Vvi-Vitvi07g01233\_t001 |  | \| \| |  |  |  |  |  |  |  |  |  |  |
| 1 | Vvi-Vitvi07g01234\_t001 |  | \| \| |  |  |  |  |  |  |  |  |  |  |
| 1 | Vvi-Vitvi07g04479\_t001 |  | \| \| |  |  |  |  |  |  |  |  |  |  |
| 1 | Vvi-Vitvi07g01236\_t001 |  | \| \| |  |  |  |  |  |  |  |  |  |  |
| 1 | Vvi-Vitvi07g01237\_t001 |  | \| \| |  |  |  |  |  |  |  |  |  |  |
| 1 | Vvi-Vitvi07g02992\_t001 |  | Atr-ERN20196 |  |  |  |  |  |  |  |  |  |  |
| 1 | Vvi-Vitvi07g02991\_t001 |  | \| \| |  |  |  |  |  |  |  |  |  |  |
| 1 | Vvi-Vitvi07g02990\_t001 |  | Atr-ERN20205 |  |  |  |  |  |  |  |  |  |  |
| 1 | Vvi-Vitvi07g04480\_t001 |  | Atr-ERN20212 |  |  |  |  |  |  |  |  |  |  |
| 1 | Vvi-Vitvi07g04481\_t001 |  | \| \| |  |  |  |  |  |  |  |  |  |  |
| 1 | Vvi-Vitvi07g04482\_t001 |  | \| \| |  |  |  |  |  |  |  |  |  |  |
| 1 | Vvi-Vitvi07g04483\_t001 |  | \| \| |  |  |  |  |  |  |  |  |  |  |
| 1 | Vvi-Vitvi07g01242\_t001 |  | \| \| |  |  |  |  |  |  |  |  |  |  |
| 1 | Vvi-Vitvi07g02498\_t001 |  | Atr-ERN20214 |  |  |  |  |  |  |  |  |  |  |
| 1 | Vvi-Vitvi07g01243\_t001 |  | \| \| |  |  |  |  |  |  |  |  |  |  |
| 1 | Vvi-Vitvi07g01244\_t001 |  | Atr-ERN20216 |  |  |  |  |  |  |  |  |  |  |
| 1 | Vvi-Vitvi07g04484\_t001 |  | \| \| |  |  |  |  |  |  |  |  |  |  |
| 1 | Vvi-Vitvi07g01246\_t001 |  | \| \| |  |  |  |  |  |  |  |  |  |  |
| 1 | Vvi-Vitvi07g01247\_t001 |  | Atr-ERN20225 |  |  |  |  |  |  |  |  |  |  |
| 1 | Vvi-Vitvi07g04485\_t002 |  | Atr-ERN20231 |  |  |  |  |  |  |  |  |  |  |
| 1 | Vvi-Vitvi07g01249\_t001 |  | \| \| |  |  |  |  |  |  |  |  |  |  |
| 1 | Vvi-Vitvi07g01250\_t001 |  | \| \| |  |  |  |  |  |  |  |  |  |  |
| 1 | Vvi-Vitvi07g01251\_t001 |  | \| \| |  |  |  |  |  |  |  |  |  |  |
| 1 | Vvi-Vitvi07g01252\_t001 |  | \| \| |  |  |  |  |  |  |  |  |  |  |
| 1 | Vvi-Vitvi07g01253\_t001 |  | \| \| |  |  |  |  |  |  |  |  |  |  |
| 1 | Vvi-Vitvi07g02984\_t001 |  | \| \| |  |  |  |  |  |  |  |  |  |  |
| 1 | Vvi-Vitvi07g02983\_t001 |  | \| \| |  |  |  |  |  |  |  |  |  |  |
| 1 | Vvi-Vitvi07g02982\_t001 |  | \| \| |  |  |  |  |  |  |  |  |  |  |
| 1 | Vvi-Vitvi07g04486\_t001 |  | Atr-ERN20241 |  |  |  |  |  |  |  |  |  |  |
| 0 | Vvi-Vitvi07g04487\_t001 |  |  |  |  |  |  |  |  |  |  |  |  |
| 0 | Vvi-Vitvi07g01255\_t001 |  |  |  |  |  |  |  |  |  |  |  |  |
| 0 | Vvi-Vitvi07g02500\_t002 |  |  |  |  |  |  |  |  |  |  |  |  |
| 0 | Vvi-Vitvi07g04488\_t001 |  |  |  |  |  |  |  |  |  |  |  |  |
| 0 | Vvi-Vitvi07g04489\_t001 |  |  |  |  |  |  |  |  |  |  |  |  |
| 1 | Vvi-Vitvi07g01261\_t001 |  | Atr-ERN17622 |  |  |  |  |  |  |  |  |  |  |
| 1 | Vvi-Vitvi07g01262\_t001 |  | \| \| |  |  |  |  |  |  |  |  |  |  |
| 1 | Vvi-Vitvi07g04490\_t001 |  | \| \| |  |  |  |  |  |  |  |  |  |  |
| 1 | Vvi-Vitvi07g04491\_t001 |  | \| \| |  |  |  |  |  |  |  |  |  |  |
| 1 | Vvi-Vitvi07g01264\_t001 |  | Atr-ERN17626 |  |  |  |  |  |  |  |  |  |  |
| 1 | Vvi-Vitvi07g01265\_t001 |  | Atr-ERN17627 |  |  |  |  |  |  |  |  |  |  |
| 1 | Vvi-Vitvi07g01266\_t001 |  | Atr-ERN17630 |  |  |  |  |  |  |  |  |  |  |
| 1 | Vvi-Vitvi07g04492\_t001 |  | \| \| |  |  |  |  |  |  |  |  |  |  |
| 1 | Vvi-Vitvi07g02502\_t001 |  | Atr-ERN17631 |  |  |  |  |  |  |  |  |  |  |
| 1 | Vvi-Vitvi07g01267\_t001 |  | \| \| |  |  |  |  |  |  |  |  |  |  |
| 1 | Vvi-Vitvi07g01273\_t001 |  | \| \| |  |  |  |  |  |  |  |  |  |  |
| 1 | Vvi-Vitvi07g01274\_t001 |  | Atr-ERN17642 |  |  |  |  |  |  |  |  |  |  |
| 1 | Vvi-Vitvi07g04493\_t001 |  | \| \| |  |  |  |  |  |  |  |  |  |  |
| 1 | Vvi-Vitvi07g01275\_t001 |  | \| \| |  |  |  |  |  |  |  |  |  |  |
| 1 | Vvi-Vitvi07g01276\_t001 |  | Atr-ERN17647 |  |  |  |  |  |  |  |  |  |  |
| 1 | Vvi-Vitvi07g04494\_t001 |  | \| \| |  |  |  |  |  |  |  |  |  |  |
| 1 | Vvi-Vitvi07g01277\_t001 |  | Atr-ERN17651 |  |  |  |  |  |  |  |  |  |  |
| 1 | Vvi-Vitvi07g01278\_t002 |  | Atr-ERN17652 |  |  |  |  |  |  |  |  |  |  |
| 1 | Vvi-Vitvi07g01279\_t001 |  | Atr-ERN17653 |  |  |  |  |  |  |  |  |  |  |
| 1 | Vvi-Vitvi07g02504\_t001 |  | \| \| |  |  |  |  |  |  |  |  |  |  |
| 1 | Vvi-Vitvi07g01280\_t001 |  | Atr-ERN17667 |  |  |  |  |  |  |  |  |  |  |
| 1 | Vvi-Vitvi07g01281\_t001 |  | \| \| |  |  |  |  |  |  |  |  |  |  |
| 1 | Vvi-Vitvi07g04495\_t001 |  | \| \| |  |  |  |  |  |  |  |  |  |  |
| 1 | Vvi-Vitvi07g04496\_t001 |  | Atr-ERN17671 |  |  |  |  |  |  |  |  |  |  |
| 1 | Vvi-Vitvi07g04497\_t001 |  | \| \| |  |  |  |  |  |  |  |  |  |  |
| 1 | Vvi-Vitvi07g04498\_t001 |  | \| \| |  |  |  |  |  |  |  |  |  |  |
| 1 | Vvi-Vitvi07g01286\_t001 |  | Atr-ERN17673 |  |  |  |  |  |  |  |  |  |  |
| 1 | Vvi-Vitvi07g04499\_t001 |  | \| \| |  |  |  |  |  |  |  |  |  |  |
| 1 | Vvi-Vitvi07g01287\_t001 |  | Atr-ERN17674 |  |  |  |  |  |  |  |  |  |  |
| 1 | Vvi-Vitvi07g04500\_t001 |  | \| \| |  |  |  |  |  |  |  |  |  |  |
| 1 | Vvi-Vitvi07g04501\_t001 |  | \| \| |  |  |  |  |  |  |  |  |  |  |
| 1 | Vvi-Vitvi07g01288\_t001 |  | Atr-ERN17681 |  |  |  |  |  |  |  |  |  |  |
| 1 | Vvi-Vitvi07g04502\_t001 |  | \| \| |  |  |  |  |  |  |  |  |  |  |
| 1 | Vvi-Vitvi07g04503\_t001 |  | \| \| |  |  |  |  |  |  |  |  |  |  |
| 1 | Vvi-Vitvi07g04504\_t001 |  | \| \| |  |  |  |  |  |  |  |  |  |  |
| 1 | Vvi-Vitvi07g01291\_t001 |  | \| \| |  |  |  |  |  |  |  |  |  |  |
| 1 | Vvi-Vitvi07g01292\_t001 |  | \| \| |  |  |  |  |  |  |  |  |  |  |
| 1 | Vvi-Vitvi07g01293\_t001 |  | \| \| |  |  |  |  |  |  |  |  |  |  |
| 1 | Vvi-Vitvi07g01294\_t001 |  | Atr-ERN17696 |  |  |  |  |  |  |  |  |  |  |
| 1 | Vvi-Vitvi07g01295\_t001 |  | Atr-ERN17697 |  |  |  |  |  |  |  |  |  |  |
| 1 | Vvi-Vitvi07g01296\_t001 |  | Atr-ERN17698 |  |  |  |  |  |  |  |  |  |  |
| 1 | Vvi-Vitvi07g01297\_t001 |  | Atr-ERN17702 |  |  |  |  |  |  |  |  |  |  |
| 1 | Vvi-Vitvi07g01298\_t001 |  | Atr-ERN17704 |  |  |  |  |  |  |  |  |  |  |
| 1 | Vvi-Vitvi07g04505\_t001 |  | Atr-ERN17709 |  |  |  |  |  |  |  |  |  |  |
| 1 | Vvi-Vitvi07g04506\_t001 |  | \| \| |  |  |  |  |  |  |  |  |  |  |
| 1 | Vvi-Vitvi07g04507\_t001 |  | Atr-ERN17712 |  |  |  |  |  |  |  |  |  |  |
| 0 | Vvi-Vitvi07g04508\_t001 |  |  |  |  |  |  |  |  |  |  |  |  |
| 0 | Vvi-Vitvi07g02512\_t001 |  |  |  |  |  |  |  |  |  |  |  |  |
| 0 | Vvi-Vitvi07g02513\_t001 |  |  |  |  |  |  |  |  |  |  |  |  |
| 0 | Vvi-Vitvi07g01300\_t001 |  |  |  |  |  |  |  |  |  |  |  |  |
| 0 | Vvi-Vitvi07g02516\_t001 |  |  |  |  |  |  |  |  |  |  |  |  |
| 0 | Vvi-Vitvi07g02517\_t001 |  |  |  |  |  |  |  |  |  |  |  |  |
| 0 | Vvi-Vitvi07g04509\_t001 |  |  |  |  |  |  |  |  |  |  |  |  |
| 0 | Vvi-Vitvi07g01302\_t003 |  |  |  |  |  |  |  |  |  |  |  |  |
| 0 | Vvi-Vitvi07g04510\_t001 |  |  |  |  |  |  |  |  |  |  |  |  |
| 0 | Vvi-Vitvi07g01306\_t001 |  |  |  |  |  |  |  |  |  |  |  |  |
| 0 | Vvi-Vitvi07g04511\_t001 |  |  |  |  |  |  |  |  |  |  |  |  |
| 0 | Vvi-Vitvi07g04512\_t001 |  |  |  |  |  |  |  |  |  |  |  |  |
| 0 | Vvi-Vitvi07g01308\_t002 |  |  |  |  |  |  |  |  |  |  |  |  |
| 1 | Vvi-Vitvi07g01309\_t001 |  | Atr-ERN14426 |  |  |  |  |  |  |  |  |  |  |
| 1 | Vvi-Vitvi07g04513\_t001 |  | \| \| |  |  |  |  |  |  |  |  |  |  |
| 1 | Vvi-Vitvi07g04514\_t001 |  | \| \| |  |  |  |  |  |  |  |  |  |  |
| 1 | Vvi-Vitvi07g04515\_t001 |  | \| \| |  |  |  |  |  |  |  |  |  |  |
| 1 | Vvi-Vitvi07g02519\_t001 |  | \| \| |  |  |  |  |  |  |  |  |  |  |
| 1 | Vvi-Vitvi07g01312\_t001 |  | \| \| |  |  |  |  |  |  |  |  |  |  |
| 1 | Vvi-Vitvi07g01313\_t001 |  | Atr-ERN14424 |  |  |  |  |  |  |  |  |  |  |
| 1 | Vvi-Vitvi07g02521\_t001 |  | \| \| |  |  |  |  |  |  |  |  |  |  |
| 1 | Vvi-Vitvi07g04516\_t001 |  | \| \| |  |  |  |  |  |  |  |  |  |  |
| 1 | Vvi-Vitvi07g04517\_t001 |  | \| \| |  |  |  |  |  |  |  |  |  |  |
| 1 | Vvi-Vitvi07g04518\_t001 |  | \| \| |  |  |  |  |  |  |  |  |  |  |
| 1 | Vvi-Vitvi07g01315\_t001 |  | \| \| |  |  |  |  |  |  |  |  |  |  |
| 1 | Vvi-Vitvi07g04519\_t001 |  | \| \| |  |  |  |  |  |  |  |  |  |  |
| 1 | Vvi-Vitvi07g04520\_t001 |  | \| \| |  |  |  |  |  |  |  |  |  |  |
| 1 | Vvi-Vitvi07g01317\_t001 |  | \| \| |  |  |  |  |  |  |  |  |  |  |
| 1 | Vvi-Vitvi07g04521\_t001 |  | \| \| |  |  |  |  |  |  |  |  |  |  |
| 1 | Vvi-Vitvi07g04522\_t001 |  | \| \| |  |  |  |  |  |  |  |  |  |  |
| 1 | Vvi-Vitvi07g04523\_t001 |  | \| \| |  |  |  |  |  |  |  |  |  |  |
| 1 | Vvi-Vitvi07g04524\_t001 |  | \| \| |  |  |  |  |  |  |  |  |  |  |
| 1 | Vvi-Vitvi07g04525\_t001 |  | \| \| |  |  |  |  |  |  |  |  |  |  |
| 1 | Vvi-Vitvi07g01323\_t001 |  | Atr-ERN14422 |  |  |  |  |  |  |  |  |  |  |
| 1 | Vvi-Vitvi07g04526\_t001 |  | \| \| |  |  |  |  |  |  |  |  |  |  |
| 1 | Vvi-Vitvi07g01324\_t001 |  | Atr-ERN14421 |  |  |  |  |  |  |  |  |  |  |
| 1 | Vvi-Vitvi07g01325\_t001 |  | Atr-ERN14420 |  |  |  |  |  |  |  |  |  |  |
| 1 | Vvi-Vitvi07g04527\_t001 |  | \| \| |  |  |  |  |  |  |  |  |  |  |
| 1 | Vvi-Vitvi07g02529\_t001.1.6037826f |  | Atr-ERN14419 |  |  |  |  |  |  |  |  |  |  |
| 0 | Vvi-Vitvi07g04528\_t001 |  |  |  |  |  |  |  |  |  |  |  |  |
| 0 | Vvi-Vitvi07g01326\_t001 |  |  |  |  |  |  |  |  |  |  |  |  |
| 0 | Vvi-Vitvi07g04529\_t001 |  |  |  |  |  |  |  |  |  |  |  |  |
| 0 | Vvi-Vitvi07g01327\_t001 |  |  |  |  |  |  |  |  |  |  |  |  |
| 0 | Vvi-Vitvi07g01328\_t001 |  |  |  |  |  |  |  |  |  |  |  |  |
| 0 | Vvi-Vitvi07g01329\_t001 |  |  |  |  |  |  |  |  |  |  |  |  |
| 0 | Vvi-Vitvi07g04530\_t001 |  |  |  |  |  |  |  |  |  |  |  |  |
| 0 | Vvi-Vitvi07g02075\_t001 |  |  |  |  |  |  |  |  |  |  |  |  |
| 0 | Vvi-Vitvi07g02541\_t001 |  |  |  |  |  |  |  |  |  |  |  |  |
| 0 | Vvi-Vitvi07g01368\_t003 |  |  |  |  |  |  |  |  |  |  |  |  |
| 0 | Vvi-Vitvi07g04531\_t001 |  |  |  |  |  |  |  |  |  |  |  |  |
| 0 | Vvi-Vitvi07g01366\_t001 |  |  |  |  |  |  |  |  |  |  |  |  |
| 0 | Vvi-Vitvi07g04532\_t001 |  |  |  |  |  |  |  |  |  |  |  |  |
| 0 | Vvi-Vitvi07g04533\_t001 |  |  |  |  |  |  |  |  |  |  |  |  |
| 0 | Vvi-Vitvi07g01345\_t001 |  |  |  |  |  |  |  |  |  |  |  |  |
| 0 | Vvi-Vitvi07g01342\_t001 |  |  |  |  |  |  |  |  |  |  |  |  |
| 0 | Vvi-Vitvi07g04534\_t001 |  |  |  |  |  |  |  |  |  |  |  |  |
| 0 | Vvi-Vitvi07g04535\_t001 |  |  |  |  |  |  |  |  |  |  |  |  |
| 0 | Vvi-Vitvi07g01337\_t001 |  |  |  |  |  |  |  |  |  |  |  |  |
| 0 | Vvi-Vitvi07g01335\_t001 |  |  |  |  |  |  |  |  |  |  |  |  |
| 0 | Vvi-Vitvi07g04536\_t001 |  |  |  |  |  |  |  |  |  |  |  |  |
| 0 | Vvi-Vitvi07g01333\_t001 |  |  |  |  |  |  |  |  |  |  |  |  |
| 0 | Vvi-Vitvi07g01332\_t001 |  |  |  |  |  |  |  |  |  |  |  |  |
| 0 | Vvi-Vitvi07g02531\_t001 |  |  |  |  |  |  |  |  |  |  |  |  |
| 0 | Vvi-Vitvi07g04537\_t001 |  |  |  |  |  |  |  |  |  |  |  |  |
| 0 | Vvi-Vitvi07g01331\_t001 |  |  |  |  |  |  |  |  |  |  |  |  |
| 0 | Vvi-Vitvi07g01330\_t001 |  |  |  |  |  |  |  |  |  |  |  |  |
| 0 | Vvi-Vitvi07g04538\_t001 |  |  |  |  |  |  |  |  |  |  |  |  |
| 0 | Vvi-Vitvi10g02293\_t001 |  |  |  |  |  |  |  |  |  |  |  |  |
| 0 | Vvi-Vitvi07g04539\_t001 |  |  |  |  |  |  |  |  |  |  |  |  |
| 0 | Vvi-Vitvi07g04540\_t001 |  |  |  |  |  |  |  |  |  |  |  |  |
| 0 | Vvi-Vitvi07g04541\_t001 |  |  |  |  |  |  |  |  |  |  |  |  |
| 0 | Vvi-Vitvi07g04542\_t001 |  |  |  |  |  |  |  |  |  |  |  |  |
| 1 | Vvi-Vitvi07g04543\_t001 |  | Atr-ERM94218 |  |  |  |  |  |  |  |  |  |  |
| 1 | Vvi-Vitvi07g02535\_t001 |  | Atr-ERM94217 |  |  |  |  |  |  |  |  |  |  |
| 1 | Vvi-Vitvi07g02536\_t001 |  | Atr-ERM94215 |  |  |  |  |  |  |  |  |  |  |
| 1 | Vvi-Vitvi07g02537\_t001 |  | \| \| |  |  |  |  |  |  |  |  |  |  |
| 1 | Vvi-Vitvi07g02538\_t001 |  | \| \| |  |  |  |  |  |  |  |  |  |  |
| 1 | Vvi-Vitvi07g01350\_t001 |  | Atr-ERM94214 |  |  |  |  |  |  |  |  |  |  |
| 1 | Vvi-Vitvi07g01351\_t001 |  | \| \| |  |  |  |  |  |  |  |  |  |  |
| 1 | Vvi-Vitvi07g01352\_t001 |  | Atr-ERM94212 |  |  |  |  |  |  |  |  |  |  |
| 1 | Vvi-Vitvi07g01353\_t001 |  | \| \| |  |  |  |  |  |  |  |  |  |  |
| 1 | Vvi-Vitvi07g01354\_t001 |  | Atr-ERM94201 |  |  |  |  |  |  |  |  |  |  |
| 1 | Vvi-Vitvi07g04544\_t001 |  | \| \| |  |  |  |  |  |  |  |  |  |  |
| 1 | Vvi-Vitvi07g03131\_t001 |  | \| \| |  |  |  |  |  |  |  |  |  |  |
| 1 | Vvi-Vitvi07g03026\_t001 |  | \| \| |  |  |  |  |  |  |  |  |  |  |
| 1 | Vvi-Vitvi07g04545\_t001 |  | \| \| |  |  |  |  |  |  |  |  |  |  |
| 1 | Vvi-Vitvi07g03031\_t001 |  | Atr-ERM94196 |  |  |  |  |  |  |  |  |  |  |
| 1 | Vvi-Vitvi07g04546\_t001 |  | \| \| |  |  |  |  |  |  |  |  |  |  |
| 1 | Vvi-Vitvi07g03030\_t001 |  | \| \| |  |  |  |  |  |  |  |  |  |  |
| 1 | Vvi-Vitvi07g01365\_t001 |  | Atr-ERM94185 |  |  |  |  |  |  |  |  |  |  |
| 1 | Vvi-Vitvi07g04547\_t001 |  | \| \| |  |  |  |  |  |  |  |  |  |  |
| 1 | Vvi-Vitvi07g02540\_t001 |  | Atr-ERM94184 |  |  |  |  |  |  |  |  |  |  |
| 1 | Vvi-Vitvi07g01364\_t001 |  | Atr-ERM94176 |  |  |  |  |  |  |  |  |  |  |
| 1 | Vvi-Vitvi07g01363\_t001 |  | Atr-ERM94172 |  |  |  |  |  |  |  |  |  |  |
| 1 | Vvi-Vitvi07g01361\_t001 |  | Atr-ERM94169 |  |  |  |  |  |  |  |  |  |  |
| 1 | Vvi-Vitvi07g01360\_t001 |  | Atr-ERM94168 |  |  |  |  |  |  |  |  |  |  |
| 1 | Vvi-Vitvi07g01359\_t001 |  | Atr-ERM94166 |  |  |  |  |  |  |  |  |  |  |
| 1 | Vvi-Vitvi07g01358\_t001 |  | Atr-ERM94164 |  |  |  |  |  |  |  |  |  |  |
| 1 | Vvi-Vitvi07g04548\_t001 |  | \| \| |  |  |  |  |  |  |  |  |  |  |
| 1 | Vvi-Vitvi07g04549\_t001 |  | \| \| |  |  |  |  |  |  |  |  |  |  |
| 1 | Vvi-Vitvi07g04550\_t001 |  | \| \| |  |  |  |  |  |  |  |  |  |  |
| 1 | Vvi-Vitvi07g04551\_t001 |  | Atr-ERM94160 |  |  |  |  |  |  |  |  |  |  |
| 0 | Vvi-Vitvi07g04552\_t001 |  |  |  |  |  |  |  |  |  |  |  |  |
| 0 | Vvi-Vitvi07g04553\_t001 |  |  |  |  |  |  |  |  |  |  |  |  |
| 0 | Vvi-Vitvi07g04554\_t001 |  |  |  |  |  |  |  |  |  |  |  |  |
| 0 | Vvi-Vitvi07g01380\_t001 |  |  |  |  |  |  |  |  |  |  |  |  |
| 0 | Vvi-Vitvi07g02548\_t001 |  |  |  |  |  |  |  |  |  |  |  |  |
| 0 | Vvi-Vitvi07g01381\_t001 |  |  |  |  |  |  |  |  |  |  |  |  |
| 0 | Vvi-Vitvi07g04555\_t001 |  |  |  |  |  |  |  |  |  |  |  |  |
| 0 | Vvi-Vitvi07g04556\_t002 |  |  |  |  |  |  |  |  |  |  |  |  |
| 0 | Vvi-Vitvi07g04557\_t001 |  |  |  |  |  |  |  |  |  |  |  |  |
| 0 | Vvi-Vitvi07g04558\_t001 |  |  |  |  |  |  |  |  |  |  |  |  |
| 0 | Vvi-Vitvi07g04559\_t001 |  |  |  |  |  |  |  |  |  |  |  |  |
| 0 | Vvi-Vitvi07g04560\_t001 |  |  |  |  |  |  |  |  |  |  |  |  |
| 0 | Vvi-Vitvi07g04561\_t001 |  |  |  |  |  |  |  |  |  |  |  |  |
| 0 | Vvi-Vitvi07g04562\_t001 |  |  |  |  |  |  |  |  |  |  |  |  |
| 0 | Vvi-Vitvi07g04563\_t001 |  |  |  |  |  |  |  |  |  |  |  |  |
| 0 | Vvi-Vitvi07g03178\_t001 |  |  |  |  |  |  |  |  |  |  |  |  |
| 0 | Vvi-Vitvi07g04564\_t001 |  |  |  |  |  |  |  |  |  |  |  |  |
| 0 | Vvi-Vitvi07g04565\_t001 |  |  |  |  |  |  |  |  |  |  |  |  |
| 0 | Vvi-Vitvi07g04566\_t001 |  |  |  |  |  |  |  |  |  |  |  |  |
| 0 | Vvi-Vitvi07g04567\_t001 |  |  |  |  |  |  |  |  |  |  |  |  |
| 0 | Vvi-Vitvi07g04568\_t001 |  |  |  |  |  |  |  |  |  |  |  |  |
| 0 | Vvi-Vitvi07g04569\_t001 |  |  |  |  |  |  |  |  |  |  |  |  |
| 0 | Vvi-Vitvi07g04570\_t001 |  |  |  |  |  |  |  |  |  |  |  |  |
| 0 | Vvi-Vitvi07g04571\_t001 |  |  |  |  |  |  |  |  |  |  |  |  |
| 0 | Vvi-Vitvi07g04572\_t001 |  |  |  |  |  |  |  |  |  |  |  |  |
| 0 | Vvi-Vitvi07g04573\_t001 |  |  |  |  |  |  |  |  |  |  |  |  |
| 0 | Vvi-Vitvi07g01392\_t001 |  |  |  |  |  |  |  |  |  |  |  |  |
| 0 | Vvi-Vitvi07g04574\_t001 |  |  |  |  |  |  |  |  |  |  |  |  |
| 0 | Vvi-Vitvi07g01393\_t001 |  |  |  |  |  |  |  |  |  |  |  |  |
| 0 | Vvi-Vitvi07g01394\_t001 |  |  |  |  |  |  |  |  |  |  |  |  |
| 0 | Vvi-Vitvi07g02558\_t001 |  |  |  |  |  |  |  |  |  |  |  |  |
| 0 | Vvi-Vitvi07g03041\_t001 |  |  |  |  |  |  |  |  |  |  |  |  |
| 0 | Vvi-Vitvi07g02560\_t001 |  |  |  |  |  |  |  |  |  |  |  |  |
| 0 | Vvi-Vitvi07g01396\_t001 |  |  |  |  |  |  |  |  |  |  |  |  |
| 0 | Vvi-Vitvi07g03044\_t001 |  |  |  |  |  |  |  |  |  |  |  |  |
| 0 | Vvi-Vitvi07g04575\_t001 |  |  |  |  |  |  |  |  |  |  |  |  |
| 0 | Vvi-Vitvi07g04576\_t001 |  |  |  |  |  |  |  |  |  |  |  |  |
| 0 | Vvi-Vitvi07g03046\_t001 |  |  |  |  |  |  |  |  |  |  |  |  |
| 1 | Vvi-Vitvi07g04577\_t001 |  | Atr-ERM95478 |  |  |  |  |  |  |  |  |  |  |
| 1 | Vvi-Vitvi07g04578\_t001 |  | \| \| |  |  |  |  |  |  |  |  |  |  |
| 1 | Vvi-Vitvi07g03049\_t001 |  | Atr-ERM95470 |  |  |  |  |  |  |  |  |  |  |
| 1 | Vvi-Vitvi07g04579\_t001 |  | \| \| |  |  |  |  |  |  |  |  |  |  |
| 1 | Vvi-Vitvi07g04580\_t001 |  | \| \| |  |  |  |  |  |  |  |  |  |  |
| 1 | Vvi-Vitvi07g04581\_t001 |  | \| \| |  |  |  |  |  |  |  |  |  |  |
| 1 | Vvi-Vitvi07g04582\_t001 |  | \| \| |  |  |  |  |  |  |  |  |  |  |
| 1 | Vvi-Vitvi07g03112\_t001 |  | \| \| |  |  |  |  |  |  |  |  |  |  |
| 1 | Vvi-Vitvi07g02605\_t001 |  | \| \| |  |  |  |  |  |  |  |  |  |  |
| 1 | Vvi-Vitvi07g02604\_t001 |  | \| \| |  |  |  |  |  |  |  |  |  |  |
| 1 | Vvi-Vitvi07g04583\_t001 |  | \| \| |  |  |  |  |  |  |  |  |  |  |
| 1 | Vvi-Vitvi07g01504\_t001 |  | \| \| |  |  |  |  |  |  |  |  |  |  |
| 1 | Vvi-Vitvi07g04584\_t001 |  | \| \| |  |  |  |  |  |  |  |  |  |  |
| 1 | Vvi-Vitvi07g01503\_t001 |  | \| \| |  |  |  |  |  |  |  |  |  |  |
| 1 | Vvi-Vitvi07g04585\_t001 |  | \| \| |  |  |  |  |  |  |  |  |  |  |
| 1 | Vvi-Vitvi07g01502\_t001 |  | \| \| |  |  |  |  |  |  |  |  |  |  |
| 1 | Vvi-Vitvi07g01501\_t001 |  | \| \| |  |  |  |  |  |  |  |  |  |  |
| 1 | Vvi-Vitvi07g01500\_t001 |  | \| \| |  |  |  |  |  |  |  |  |  |  |
| 1 | Vvi-Vitvi07g01499\_t001 |  | \| \| |  |  |  |  |  |  |  |  |  |  |
| 1 | Vvi-Vitvi07g04586\_t001 |  | \| \| |  |  |  |  |  |  |  |  |  |  |
| 1 | Vvi-Vitvi07g01498\_t001 |  | Atr-ERM95455 |  |  |  |  |  |  |  |  |  |  |
| 1 | Vvi-Vitvi07g04587\_t001 |  | Atr-ERM95431 |  |  |  |  |  |  |  |  |  |  |
| 1 | Vvi-Vitvi07g01495\_t001 |  | \| \| |  |  |  |  |  |  |  |  |  |  |
| 1 | Vvi-Vitvi07g01494\_t001 |  | Atr-ERM95420 |  |  |  |  |  |  |  |  |  |  |
| 1 | Vvi-Vitvi07g01492\_t001 |  | Atr-ERM95402 |  |  |  |  |  |  |  |  |  |  |
| 1 | Vvi-Vitvi07g01489\_t001 |  | Atr-ERM95396 |  |  |  |  |  |  |  |  |  |  |
| 1 | Vvi-Vitvi07g04588\_t001 |  | \| \| |  |  |  |  |  |  |  |  |  |  |
| 1 | Vvi-Vitvi07g01488\_t001 |  | Atr-ERM95386 |  |  |  |  |  |  |  |  |  |  |
| 1 | Vvi-Vitvi07g01482\_t001 |  | Atr-ERM95384 |  |  |  |  |  |  |  |  |  |  |
| 1 | Vvi-Vitvi07g01480\_t001 |  | \| \| |  |  |  |  |  |  |  |  |  |  |
| 1 | Vvi-Vitvi07g02602\_t002 |  | \| \| |  |  |  |  |  |  |  |  |  |  |
| 1 | Vvi-Vitvi07g01476\_t001 |  | Atr-ERM95369 |  |  |  |  |  |  |  |  |  |  |
| 1 | Vvi-Vitvi07g01475\_t001 |  | Atr-ERM95366 |  |  |  |  |  |  |  |  |  |  |
| 1 | Vvi-Vitvi07g01473\_t001 |  | Atr-ERM95364 |  |  |  |  |  |  |  |  |  |  |
| 1 | Vvi-Vitvi07g02600\_t001 |  | Atr-ERM95363 |  |  |  |  |  |  |  |  |  |  |
| 1 | Vvi-Vitvi07g01472\_t001 |  | \| \| |  |  |  |  |  |  |  |  |  |  |
| 1 | Vvi-Vitvi07g04589\_t001 |  | \| \| |  |  |  |  |  |  |  |  |  |  |
| 1 | Vvi-Vitvi07g04590\_t001 |  | Atr-ERM95355 |  |  |  |  |  |  |  |  |  |  |
| 1 | Vvi-Vitvi07g01471\_t001 |  | \| \| |  |  |  |  |  |  |  |  |  |  |
| 1 | Vvi-Vitvi07g04591\_t001 |  | Atr-ERM95347 |  |  |  |  |  |  |  |  |  |  |
| 0 | Vvi-Vitvi07g01469\_t001 |  |  |  |  |  |  |  |  |  |  |  |  |
| 0 | Vvi-Vitvi07g02596\_t001 |  |  |  |  |  |  |  |  |  |  |  |  |
| 1 | Vvi-Vitvi07g01468\_t001 |  | Atr-ERN02923 |  |  |  |  |  |  |  |  |  |  |
| 1 | Vvi-Vitvi07g04592\_t001 |  | Atr-ERN02921 |  |  |  |  |  |  |  |  |  |  |
| 1 | Vvi-Vitvi07g04593\_t001 |  | \| \| |  |  |  |  |  |  |  |  |  |  |
| 1 | Vvi-Vitvi07g04594\_t001 |  | \| \| |  |  |  |  |  |  |  |  |  |  |
| 1 | Vvi-Vitvi07g01465\_t001 |  | \| \| |  |  |  |  |  |  |  |  |  |  |
| 1 | Vvi-Vitvi07g02594\_t001 |  | Atr-ERN02918 |  |  |  |  |  |  |  |  |  |  |
| 1 | Vvi-Vitvi07g01462\_t001 |  | \| \| |  |  |  |  |  |  |  |  |  |  |
| 1 | Vvi-Vitvi07g01460\_t001 |  | Atr-ERN02909 |  |  |  |  |  |  |  |  |  |  |
| 1 | Vvi-Vitvi07g04595\_t001 |  | \| \| |  |  |  |  |  |  |  |  |  |  |
| 1 | Vvi-Vitvi07g01459\_t001 |  | \| \| |  |  |  |  |  |  |  |  |  |  |
| 1 | Vvi-Vitvi07g04596\_t001 |  | \| \| |  |  |  |  |  |  |  |  |  |  |
| 1 | Vvi-Vitvi07g01457\_t001 |  | Atr-ERN02906 |  |  |  |  |  |  |  |  |  |  |
| 1 | Vvi-Vitvi07g02591\_t001 |  | \| \| |  |  |  |  |  |  |  |  |  |  |
| 1 | Vvi-Vitvi07g02588\_t002 |  | \| \| |  |  |  |  |  |  |  |  |  |  |
| 1 | Vvi-Vitvi07g02589\_t001 |  | Atr-ERN02903 |  |  |  |  |  |  |  |  |  |  |
| 1 | Vvi-Vitvi07g01451\_t001 |  | Atr-ERN02897 |  |  |  |  |  |  |  |  |  |  |
| 1 | Vvi-Vitvi07g04597\_t001 |  | \| \| |  |  |  |  |  |  |  |  |  |  |
| 1 | Vvi-Vitvi07g04598\_t001 |  | \| \| |  |  |  |  |  |  |  |  |  |  |
| 1 | Vvi-Vitvi07g04599\_t001 |  | Atr-ERN02890 |  |  |  |  |  |  |  |  |  |  |
| 1 | Vvi-Vitvi07g04600\_t001 |  | Atr-ERN02888 |  |  |  |  |  |  |  |  |  |  |
| 1 | Vvi-Vitvi07g01449\_t001 |  | Atr-ERN02883 |  |  |  |  |  |  |  |  |  |  |
| 1 | Vvi-Vitvi07g02582\_t001 |  | Atr-ERN02881 |  |  |  |  |  |  |  |  |  |  |
| 0 | Vvi-Vitvi07g02577\_t001 |  |  |  |  |  |  |  |  |  |  |  |  |
| 0 | Vvi-Vitvi07g04601\_t001 |  |  |  |  |  |  |  |  |  |  |  |  |
| 0 | Vvi-Vitvi07g02580\_t001 |  |  |  |  |  |  |  |  |  |  |  |  |
| 0 | Vvi-Vitvi07g04602\_t001 |  |  |  |  |  |  |  |  |  |  |  |  |
| 0 | Vvi-Vitvi07g04603\_t001 |  |  |  |  |  |  |  |  |  |  |  |  |
| 0 | Vvi-Vitvi07g01444\_t001 |  |  |  |  |  |  |  |  |  |  |  |  |
| 0 | Vvi-Vitvi07g04604\_t001 |  |  |  |  |  |  |  |  |  |  |  |  |
| 0 | Vvi-Vitvi07g01442\_t001 |  |  |  |  |  |  |  |  |  |  |  |  |
| 0 | Vvi-Vitvi07g04605\_t001 |  |  |  |  |  |  |  |  |  |  |  |  |
| 0 | Vvi-Vitvi07g01441\_t001 |  |  |  |  |  |  |  |  |  |  |  |  |
| 1 | Vvi-Vitvi07g04606\_t001 |  | Atr-ERN14413 |  |  |  |  |  |  |  |  |  |  |
| 1 | Vvi-Vitvi07g01439\_t001 |  | Atr-ERN14412 |  |  |  |  |  |  |  |  |  |  |
| 1 | Vvi-Vitvi07g01437\_t001 |  | Atr-ERN14410 |  |  |  |  |  |  |  |  |  |  |
| 1 | Vvi-Vitvi07g04607\_t001 |  | Atr-ERN14408 |  |  |  |  |  |  |  |  |  |  |
| 1 | Vvi-Vitvi07g01433\_t001 |  | Atr-ERN14405 |  |  |  |  |  |  |  |  |  |  |
| 1 | Vvi-Vitvi07g01431\_t001 |  | \| \| |  |  |  |  |  |  |  |  |  |  |
| 1 | Vvi-Vitvi07g04608\_t001 |  | Atr-ERN14396 |  |  |  |  |  |  |  |  |  |  |
| 1 | Vvi-Vitvi07g04609\_t001 |  | \| \| |  |  |  |  |  |  |  |  |  |  |
| 1 | Vvi-Vitvi07g01428\_t001 |  | Atr-ERN14394 |  |  |  |  |  |  |  |  |  |  |
| 1 | Vvi-Vitvi07g01425\_t002 |  | \| \| |  |  |  |  |  |  |  |  |  |  |
| 1 | Vvi-Vitvi07g04610\_t001 |  | \| \| |  |  |  |  |  |  |  |  |  |  |
| 1 | Vvi-Vitvi07g04611\_t002 |  | Atr-ERN14389 |  |  |  |  |  |  |  |  |  |  |
| 1 | Vvi-Vitvi07g01423\_t001 |  | \| \| |  |  |  |  |  |  |  |  |  |  |
| 1 | Vvi-Vitvi07g01419\_t001 |  | \| \| |  |  |  |  |  |  |  |  |  |  |
| 1 | Vvi-Vitvi07g02572\_t001 |  | Atr-ERN14380 |  |  |  |  |  |  |  |  |  |  |
| 1 | Vvi-Vitvi07g04612\_t001 |  | \| \| |  |  |  |  |  |  |  |  |  |  |
| 1 | Vvi-Vitvi07g04613\_t001 |  | \| \| |  |  |  |  |  |  |  |  |  |  |
| 1 | Vvi-Vitvi07g04614\_t001 |  | \| \| |  |  |  |  |  |  |  |  |  |  |
| 1 | Vvi-Vitvi07g04615\_t001 |  | Atr-ERN14369 |  |  |  |  |  |  |  |  |  |  |
| 1 | Vvi-Vitvi07g04616\_t001 |  | \| \| |  |  |  |  |  |  |  |  |  |  |
| 1 | Vvi-Vitvi07g04617\_t001 |  | Atr-ERN14365 |  |  |  |  |  |  |  |  |  |  |
| 1 | Vvi-Vitvi07g01415\_t001 |  | \| \| |  |  |  |  |  |  |  |  |  |  |
| 1 | Vvi-Vitvi07g04618\_t001 |  | \| \| |  |  |  |  |  |  |  |  |  |  |
| 1 | Vvi-Vitvi07g04619\_t001 |  | \| \| |  |  |  |  |  |  |  |  |  |  |
| 1 | Vvi-Vitvi07g01414\_t001 |  | \| \| |  |  |  |  |  |  |  |  |  |  |
| 1 | Vvi-Vitvi07g02564\_t001 |  | \| \| |  |  |  |  |  |  |  |  |  |  |
| 1 | Vvi-Vitvi07g04620\_t001 |  | \| \| |  |  |  |  |  |  |  |  |  |  |
| 1 | Vvi-Vitvi07g01412\_t001 |  | \| \| |  |  |  |  |  |  |  |  |  |  |
| 1 | Vvi-Vitvi07g01411\_t001 |  | \| \| |  |  |  |  |  |  |  |  |  |  |
| 1 | Vvi-Vitvi07g02563\_t001 |  | \| \| |  |  |  |  |  |  |  |  |  |  |
| 1 | Vvi-Vitvi07g01410\_t001 |  | \| \| |  |  |  |  |  |  |  |  |  |  |
| 1 | Vvi-Vitvi07g04621\_t001 |  | \| \| |  |  |  |  |  |  |  |  |  |  |
| 1 | Vvi-Vitvi07g01408\_t001 |  | Atr-ERN14360 |  |  |  |  |  |  |  |  |  |  |
| 1 | Vvi-Vitvi07g01407\_t001 |  | \| \| |  |  |  |  |  |  |  |  |  |  |
| 2 | Vvi-Vitvi07g01406\_t001 |  | \| \| |  | Atr-ERN18022 |  |  |  |  |  |  |  |  |
| 2 | Vvi-Vitvi07g01405\_t001 |  | \| \| |  | \| \| |  |  |  |  |  |  |  |  |
| 2 | Vvi-Vitvi07g01403\_t001 |  | Atr-ERN14349 |  | \| \| |  |  |  |  |  |  |  |  |
| 2 | Vvi-Vitvi07g01402\_t001 |  | Atr-ERN14348 |  | \| \| |  |  |  |  |  |  |  |  |
| 2 | Vvi-Vitvi07g04622\_t001 |  | \| \| |  | \| \| |  |  |  |  |  |  |  |  |
| 2 | Vvi-Vitvi07g01401\_t001 |  | Atr-ERN14347 |  | \| \| |  |  |  |  |  |  |  |  |
| 1 | Vvi-Vitvi07g01400\_t002 |  |  |  | \| \| |  |  |  |  |  |  |  |  |
| 1 | Vvi-Vitvi07g04623\_t001 |  |  |  | \| \| |  |  |  |  |  |  |  |  |
| 1 | Vvi-Vitvi07g04624\_t001 |  |  |  | \| \| |  |  |  |  |  |  |  |  |
| 1 | Vvi-Vitvi07g04625\_t001 |  |  |  | \| \| |  |  |  |  |  |  |  |  |
| 1 | Vvi-Vitvi07g01399\_t001 |  |  |  | \| \| |  |  |  |  |  |  |  |  |
| 1 | Vvi-Vitvi07g02561\_t001 |  |  |  | \| \| |  |  |  |  |  |  |  |  |
| 1 | Vvi-Vitvi07g02556\_t001 |  |  |  | \| \| |  |  |  |  |  |  |  |  |
| 1 | Vvi-Vitvi07g02555\_t001 |  |  |  | \| \| |  |  |  |  |  |  |  |  |
| 1 | Vvi-Vitvi07g04626\_t001 |  |  |  | \| \| |  |  |  |  |  |  |  |  |
| 1 | Vvi-Vitvi07g02554\_t001 |  |  |  | \| \| |  |  |  |  |  |  |  |  |
| 1 | Vvi-Vitvi07g01389\_t001 |  |  |  | \| \| |  |  |  |  |  |  |  |  |
| 1 | Vvi-Vitvi07g02553\_t001 |  |  |  | \| \| |  |  |  |  |  |  |  |  |
| 1 | Vvi-Vitvi07g01388\_t001 |  |  |  | Atr-ERN18040 |  |  |  |  |  |  |  |  |
| 1 | Vvi-Vitvi07g01387\_t001 |  |  |  | Atr-ERN18046 |  |  |  |  |  |  |  |  |
| 1 | Vvi-Vitvi07g01386\_t001 |  |  |  | \| \| |  |  |  |  |  |  |  |  |
| 1 | Vvi-Vitvi07g01385\_t001 |  |  |  | \| \| |  |  |  |  |  |  |  |  |
| 1 | Vvi-Vitvi07g04627\_t001 |  |  |  | \| \| |  |  |  |  |  |  |  |  |
| 1 | Vvi-Vitvi07g04628\_t001 |  |  |  | \| \| |  |  |  |  |  |  |  |  |
| 1 | Vvi-Vitvi07g04629\_t001 |  |  |  | \| \| |  |  |  |  |  |  |  |  |
| 1 | Vvi-Vitvi07g04630\_t001 |  |  |  | \| \| |  |  |  |  |  |  |  |  |
| 1 | Vvi-Vitvi07g01520\_t001 |  |  |  | Atr-ERN18058 |  |  |  |  |  |  |  |  |
| 1 | Vvi-Vitvi07g04631\_t001 |  |  |  | \| \| |  |  |  |  |  |  |  |  |
| 1 | Vvi-Vitvi07g02610\_t001 |  |  |  | Atr-ERN18075 |  |  |  |  |  |  |  |  |
| 1 | Vvi-Vitvi07g04632\_t001 |  |  |  | \| \| |  |  |  |  |  |  |  |  |
| 1 | Vvi-Vitvi07g01516\_t001 |  |  |  | \| \| |  |  |  |  |  |  |  |  |
| 1 | Vvi-Vitvi07g04633\_t001 |  |  |  | \| \| |  |  |  |  |  |  |  |  |
| 1 | Vvi-Vitvi07g04634\_t001 |  |  |  | \| \| |  |  |  |  |  |  |  |  |
| 1 | Vvi-Vitvi07g02608\_t009 |  |  |  | \| \| |  |  |  |  |  |  |  |  |
| 1 | Vvi-Vitvi07g04635\_t001 |  |  |  | \| \| |  |  |  |  |  |  |  |  |
| 1 | Vvi-Vitvi07g01511\_t001 |  |  |  | \| \| |  |  |  |  |  |  |  |  |
| 1 | Vvi-Vitvi07g01510\_t001 |  |  |  | Atr-ERN18078 |  |  |  |  |  |  |  |  |
| 1 | Vvi-Vitvi07g04636\_t002 |  |  |  | Atr-ERN18085 |  |  |  |  |  |  |  |  |
| 1 | Vvi-Vitvi07g01508\_t001 |  |  |  | Atr-ERN18086 |  |  |  |  |  |  |  |  |
| 1 | Vvi-Vitvi07g01507\_t001 |  |  |  | Atr-ERN18090 |  |  |  |  |  |  |  |  |
| 1 | Vvi-Vitvi07g01506\_t001 |  |  |  | Atr-ERN18091 |  |  |  |  |  |  |  |  |
| 1 | Vvi-Vitvi07g01524\_t001 |  |  |  | Atr-ERN18094 |  |  |  |  |  |  |  |  |
| 1 | Vvi-Vitvi07g01523\_t001 |  |  |  | Atr-ERN18098 |  |  |  |  |  |  |  |  |
| 1 | Vvi-Vitvi07g04637\_t001 |  |  |  | Atr-ERN18099 |  |  |  |  |  |  |  |  |
| 0 | Vvi-Vitvi07g04638\_t001 |  |  |  |  |  |  |  |  |  |  |  |  |
| 0 | Vvi-Vitvi07g01522\_t001 |  |  |  |  |  |  |  |  |  |  |  |  |
| 0 | Vvi-Vitvi07g02613\_t001 |  |  |  |  |  |  |  |  |  |  |  |  |
| 0 | Vvi-Vitvi07g01543\_t001 |  |  |  |  |  |  |  |  |  |  |  |  |
| 0 | Vvi-Vitvi07g04639\_t001 |  |  |  |  |  |  |  |  |  |  |  |  |
| 0 | Vvi-Vitvi07g04640\_t001 |  |  |  |  |  |  |  |  |  |  |  |  |
| 0 | Vvi-Vitvi07g04641\_t001 |  |  |  |  |  |  |  |  |  |  |  |  |
| 0 | Vvi-Vitvi07g04642\_t003 |  |  |  |  |  |  |  |  |  |  |  |  |
| 0 | Vvi-Vitvi07g04643\_t001 |  |  |  |  |  |  |  |  |  |  |  |  |
| 1 | Vvi-Vitvi07g04644\_t001 |  | Atr-ERN14167 |  |  |  |  |  |  |  |  |  |  |
| 1 | Vvi-Vitvi07g01541\_t001 |  | \| \| |  |  |  |  |  |  |  |  |  |  |
| 1 | Vvi-Vitvi07g04645\_t001 |  | \| \| |  |  |  |  |  |  |  |  |  |  |
| 1 | Vvi-Vitvi07g04646\_t001 |  | \| \| |  |  |  |  |  |  |  |  |  |  |
| 1 | Vvi-Vitvi07g04647\_t001 |  | \| \| |  |  |  |  |  |  |  |  |  |  |
| 1 | Vvi-Vitvi07g01538\_t001 |  | \| \| |  |  |  |  |  |  |  |  |  |  |
| 1 | Vvi-Vitvi07g04648\_t001 |  | Atr-ERN14177 |  |  |  |  |  |  |  |  |  |  |
| 1 | Vvi-Vitvi07g01536\_t001 |  | Atr-ERN14178 |  |  |  |  |  |  |  |  |  |  |
| 1 | Vvi-Vitvi07g01535\_t001 |  | Atr-ERN14183 |  |  |  |  |  |  |  |  |  |  |
| 1 | Vvi-Vitvi07g01534\_t001 |  | \| \| |  |  |  |  |  |  |  |  |  |  |
| 1 | Vvi-Vitvi07g01533\_t001 |  | \| \| |  |  |  |  |  |  |  |  |  |  |
| 1 | Vvi-Vitvi07g01532\_t001 |  | Atr-ERN14196 |  |  |  |  |  |  |  |  |  |  |
| 1 | Vvi-Vitvi07g01531\_t001 |  | \| \| |  |  |  |  |  |  |  |  |  |  |
| 1 | Vvi-Vitvi07g01530\_t001 |  | \| \| |  |  |  |  |  |  |  |  |  |  |
| 1 | Vvi-Vitvi07g01529\_t001 |  | \| \| |  |  |  |  |  |  |  |  |  |  |
| 1 | Vvi-Vitvi07g04649\_t001 |  | \| \| |  |  |  |  |  |  |  |  |  |  |
| 1 | Vvi-Vitvi07g03072\_t001 |  | \| \| |  |  |  |  |  |  |  |  |  |  |
| 1 | Vvi-Vitvi07g04650\_t001 |  | \| \| |  |  |  |  |  |  |  |  |  |  |
| 1 | Vvi-Vitvi07g04651\_t001 |  | \| \| |  |  |  |  |  |  |  |  |  |  |
| 1 | Vvi-Vitvi07g04652\_t001 |  | Atr-ERN14197 |  |  |  |  |  |  |  |  |  |  |
| 1 | Vvi-Vitvi07g04653\_t001 |  | \| \| |  |  |  |  |  |  |  |  |  |  |
| 1 | Vvi-Vitvi07g03070\_t001 |  | Atr-ERN14199 |  |  |  |  |  |  |  |  |  |  |
| 1 | Vvi-Vitvi07g04654\_t001 |  | Atr-ERN14208 |  |  |  |  |  |  |  |  |  |  |
| 1 | Vvi-Vitvi07g04655\_t001 |  | \| \| |  |  |  |  |  |  |  |  |  |  |
| 1 | Vvi-Vitvi07g04656\_t001 |  | Atr-ERN14213 |  |  |  |  |  |  |  |  |  |  |
| 1 | Vvi-Vitvi07g04657\_t001 |  | Atr-ERN14216 |  |  |  |  |  |  |  |  |  |  |
| 1 | Vvi-Vitvi07g04658\_t001 |  | Atr-ERN14231 |  |  |  |  |  |  |  |  |  |  |
| 1 | Vvi-Vitvi07g04659\_t001 |  | \| \| |  |  |  |  |  |  |  |  |  |  |
| 1 | Vvi-Vitvi07g03065\_t001 |  | \| \| |  |  |  |  |  |  |  |  |  |  |
| 1 | Vvi-Vitvi07g03064\_t001 |  | Atr-ERN14235 |  |  |  |  |  |  |  |  |  |  |
| 1 | Vvi-Vitvi07g03063\_t002 |  | Atr-ERN14236 |  |  |  |  |  |  |  |  |  |  |
| 0 | Vvi-Vitvi07g04660\_t001 |  |  |  |  |  |  |  |  |  |  |  |  |
| 0 | Vvi-Vitvi07g03061\_t001 |  |  |  |  |  |  |  |  |  |  |  |  |
| 0 | Vvi-Vitvi07g04661\_t001 |  |  |  |  |  |  |  |  |  |  |  |  |
| 0 | Vvi-Vitvi07g04662\_t001 |  |  |  |  |  |  |  |  |  |  |  |  |
| 0 | Vvi-Vitvi07g03056\_t002 |  |  |  |  |  |  |  |  |  |  |  |  |
| 0 | Vvi-Vitvi07g03099\_t001 |  |  |  |  |  |  |  |  |  |  |  |  |
| 0 | Vvi-Vitvi07g04663\_t001 |  |  |  |  |  |  |  |  |  |  |  |  |
| 0 | Vvi-Vitvi07g04664\_t001 |  |  |  |  |  |  |  |  |  |  |  |  |
| 0 | Vvi-Vitvi07g03132\_t001 |  |  |  |  |  |  |  |  |  |  |  |  |
| 0 | Vvi-Vitvi07g03055\_t001 |  |  |  |  |  |  |  |  |  |  |  |  |
| 0 | Vvi-Vitvi07g04665\_t001 |  |  |  |  |  |  |  |  |  |  |  |  |
| 0 | Vvi-Vitvi07g04666\_t001 |  |  |  |  |  |  |  |  |  |  |  |  |
| 0 | Vvi-Vitvi07g04667\_t001 |  |  |  |  |  |  |  |  |  |  |  |  |
| 0 | Vvi-Vitvi07g04668\_t001 |  |  |  |  |  |  |  |  |  |  |  |  |
| 0 | Vvi-Vitvi07g04669\_t001 |  |  |  |  |  |  |  |  |  |  |  |  |
| 0 | Vvi-Vitvi07g01570\_t001 |  |  |  |  |  |  |  |  |  |  |  |  |
| 0 | Vvi-Vitvi07g04670\_t004 |  |  |  |  |  |  |  |  |  |  |  |  |
| 0 | Vvi-Vitvi07g01572\_t001 |  |  |  |  |  |  |  |  |  |  |  |  |
| 1 | Vvi-Vitvi07g04671\_t001 |  | Atr-ERN02791 |  |  |  |  |  |  |  |  |  |  |
| 1 | Vvi-Vitvi07g01577\_t001 |  | Atr-ERN02779 |  |  |  |  |  |  |  |  |  |  |
| 1 | Vvi-Vitvi07g01578\_t001 |  | \| \| |  |  |  |  |  |  |  |  |  |  |
| 1 | Vvi-Vitvi07g01580\_t002 |  | Atr-ERN02774 |  |  |  |  |  |  |  |  |  |  |
| 1 | Vvi-Vitvi07g02073\_t001 |  | Atr-ERN02773 |  |  |  |  |  |  |  |  |  |  |
| 1 | Vvi-Vitvi07g04672\_t001 |  | \| \| |  |  |  |  |  |  |  |  |  |  |
| 1 | Vvi-Vitvi07g01581\_t001 |  | Atr-ERN02771 |  |  |  |  |  |  |  |  |  |  |
| 1 | Vvi-Vitvi07g02633\_t001 |  | Atr-ERN02767 |  |  |  |  |  |  |  |  |  |  |
| 1 | Vvi-Vitvi07g01590\_t001 |  | Atr-ERN02764 |  |  |  |  |  |  |  |  |  |  |
| 1 | Vvi-Vitvi07g01591\_t001 |  | Atr-ERN02759 |  |  |  |  |  |  |  |  |  |  |
| 1 | Vvi-Vitvi07g04673\_t001 |  | \| \| |  |  |  |  |  |  |  |  |  |  |
| 1 | Vvi-Vitvi07g01593\_t001 |  | Atr-ERN02757 |  |  |  |  |  |  |  |  |  |  |
| 0 | Vvi-Vitvi07g01595\_t001 |  |  |  |  |  |  |  |  |  |  |  |  |
| 0 | Vvi-Vitvi07g04674\_t001 |  |  |  |  |  |  |  |  |  |  |  |  |
| 0 | Vvi-Vitvi07g04675\_t001 |  |  |  |  |  |  |  |  |  |  |  |  |
| 0 | Vvi-Vitvi07g01598\_t001 |  |  |  |  |  |  |  |  |  |  |  |  |
| 0 | Vvi-Vitvi07g01599\_t001 |  |  |  |  |  |  |  |  |  |  |  |  |
| 0 | Vvi-Vitvi07g01600\_t001 |  |  |  |  |  |  |  |  |  |  |  |  |
| 0 | Vvi-Vitvi07g04676\_t001 |  |  |  |  |  |  |  |  |  |  |  |  |
| 1 | Vvi-Vitvi07g01602\_t001 |  | Atr-ERM93944 |  |  |  |  |  |  |  |  |  |  |
| 1 | Vvi-Vitvi07g01603\_t003 |  | Atr-ERM93939 |  |  |  |  |  |  |  |  |  |  |
| 1 | Vvi-Vitvi07g01604\_t001 |  | Atr-ERM93938 |  |  |  |  |  |  |  |  |  |  |
| 1 | Vvi-Vitvi07g04677\_t001 |  | \| \| |  |  |  |  |  |  |  |  |  |  |
| 1 | Vvi-Vitvi07g01605\_t001 |  | Atr-ERM93937 |  |  |  |  |  |  |  |  |  |  |
| 1 | Vvi-Vitvi07g04678\_t001 |  | \| \| |  |  |  |  |  |  |  |  |  |  |
| 1 | Vvi-Vitvi07g01606\_t001 |  | Atr-ERM93934 |  |  |  |  |  |  |  |  |  |  |
| 1 | Vvi-Vitvi07g01608\_t001 |  | Atr-ERM93933 |  |  |  |  |  |  |  |  |  |  |
| 1 | Vvi-Vitvi07g04679\_t001 |  | \| \| |  |  |  |  |  |  |  |  |  |  |
| 1 | Vvi-Vitvi07g02635\_t001 |  | \| \| |  |  |  |  |  |  |  |  |  |  |
| 1 | Vvi-Vitvi07g04680\_t001 |  | \| \| |  |  |  |  |  |  |  |  |  |  |
| 1 | Vvi-Vitvi07g01610\_t001 |  | Atr-ERM93932 |  |  |  |  |  |  |  |  |  |  |
| 1 | Vvi-Vitvi07g01612\_t001 |  | Atr-ERM93927 |  |  |  |  |  |  |  |  |  |  |
| 1 | Vvi-Vitvi07g01613\_t001 |  | Atr-ERN03646 |  |  |  |  |  |  |  |  |  |  |
| 1 | Vvi-Vitvi07g01614\_t001 |  | \| \| |  |  |  |  |  |  |  |  |  |  |
| 1 | Vvi-Vitvi07g04681\_t001 |  | \| \| |  |  |  |  |  |  |  |  |  |  |
| 1 | Vvi-Vitvi07g01616\_t001 |  | \| \| |  |  |  |  |  |  |  |  |  |  |
| 1 | Vvi-Vitvi07g01617\_t001 |  | \| \| |  |  |  |  |  |  |  |  |  |  |
| 1 | Vvi-Vitvi07g01619\_t001 |  | \| \| |  |  |  |  |  |  |  |  |  |  |
| 1 | Vvi-Vitvi07g01620\_t002 |  | Atr-ERN03656 |  |  |  |  |  |  |  |  |  |  |
| 1 | Vvi-Vitvi07g01621\_t001 |  | Atr-ERN03663 |  |  |  |  |  |  |  |  |  |  |
| 1 | Vvi-Vitvi07g01622\_t001 |  | \| \| |  |  |  |  |  |  |  |  |  |  |
| 1 | Vvi-Vitvi07g04682\_t001 |  | \| \| |  |  |  |  |  |  |  |  |  |  |
| 1 | Vvi-Vitvi07g01623\_t001 |  | \| \| |  |  |  |  |  |  |  |  |  |  |
| 1 | Vvi-Vitvi07g01624\_t001 |  | Atr-ERN03667 |  |  |  |  |  |  |  |  |  |  |
| 1 | Vvi-Vitvi07g01625\_t001 |  | Atr-ERN03668 |  |  |  |  |  |  |  |  |  |  |
| 1 | Vvi-Vitvi07g01626\_t001 |  | Atr-ERN03669 |  |  |  |  |  |  |  |  |  |  |
| 1 | Vvi-Vitvi07g01627\_t001 |  | Atr-ERN03672 |  |  |  |  |  |  |  |  |  |  |
| 1 | Vvi-Vitvi07g04683\_t001 |  | \| \| |  |  |  |  |  |  |  |  |  |  |
| 1 | Vvi-Vitvi07g01630\_t001 |  | Atr-ERN03683 |  |  |  |  |  |  |  |  |  |  |
| 1 | Vvi-Vitvi07g02637\_t001 |  | \| \| |  |  |  |  |  |  |  |  |  |  |
| 1 | Vvi-Vitvi07g01631\_t001 |  | \| \| |  |  |  |  |  |  |  |  |  |  |
| 1 | Vvi-Vitvi07g01633\_t001 |  | \| \| |  |  |  |  |  |  |  |  |  |  |
| 1 | Vvi-Vitvi07g01634\_t001 |  | Atr-ERN03693 |  |  |  |  |  |  |  |  |  |  |
| 0 | Vvi-Vitvi07g01635\_t001 |  |  |  |  |  |  |  |  |  |  |  |  |
| 0 | Vvi-Vitvi07g01636\_t001 |  |  |  |  |  |  |  |  |  |  |  |  |
| 0 | Vvi-Vitvi07g01637\_t001 |  |  |  |  |  |  |  |  |  |  |  |  |
| 1 | Vvi-Vitvi07g01638\_t001 |  | Atr-ERM96714 |  |  |  |  |  |  |  |  |  |  |
| 1 | Vvi-Vitvi07g04684\_t001 |  | \| \| |  |  |  |  |  |  |  |  |  |  |
| 1 | Vvi-Vitvi07g01641\_t002 |  | Atr-ERM96713 |  |  |  |  |  |  |  |  |  |  |
| 1 | Vvi-Vitvi07g01642\_t001 |  | Atr-ERM96711 |  |  |  |  |  |  |  |  |  |  |
| 1 | Vvi-Vitvi07g04685\_t001 |  | Atr-ERM96707 |  |  |  |  |  |  |  |  |  |  |
| 1 | Vvi-Vitvi07g04686\_t001 |  | \| \| |  |  |  |  |  |  |  |  |  |  |
| 1 | Vvi-Vitvi07g01644\_t001 |  | \| \| |  |  |  |  |  |  |  |  |  |  |
| 1 | Vvi-Vitvi07g01645\_t001 |  | Atr-ERM96698 |  |  |  |  |  |  |  |  |  |  |
| 1 | Vvi-Vitvi07g01646\_t001 |  | Atr-ERM96697 |  |  |  |  |  |  |  |  |  |  |
| 0 | Vvi-Vitvi07g01647\_t003 |  |  |  |  |  |  |  |  |  |  |  |  |
| 0 | Vvi-Vitvi07g04687\_t001 |  |  |  |  |  |  |  |  |  |  |  |  |
| 0 | Vvi-Vitvi07g01648\_t001 |  |  |  |  |  |  |  |  |  |  |  |  |
| 0 | Vvi-Vitvi07g01650\_t001 |  |  |  |  |  |  |  |  |  |  |  |  |
| 0 | Vvi-Vitvi07g04688\_t001 |  |  |  |  |  |  |  |  |  |  |  |  |
| 0 | Vvi-Vitvi07g04689\_t001 |  |  |  |  |  |  |  |  |  |  |  |  |
| 0 | Vvi-Vitvi07g04690\_t001 |  |  |  |  |  |  |  |  |  |  |  |  |
| 0 | Vvi-Vitvi07g02640\_t001 |  |  |  |  |  |  |  |  |  |  |  |  |
| 0 | Vvi-Vitvi07g04691\_t001 |  |  |  |  |  |  |  |  |  |  |  |  |
| 0 | Vvi-Vitvi07g04692\_t001 |  |  |  |  |  |  |  |  |  |  |  |  |
| 0 | Vvi-Vitvi07g01657\_t001 |  |  |  |  |  |  |  |  |  |  |  |  |
| 0 | Vvi-Vitvi07g04693\_t001 |  |  |  |  |  |  |  |  |  |  |  |  |
| 0 | Vvi-Vitvi07g04694\_t001 |  |  |  |  |  |  |  |  |  |  |  |  |
| 0 | Vvi-Vitvi07g01658\_t001 |  |  |  |  |  |  |  |  |  |  |  |  |
| 0 | Vvi-Vitvi07g04695\_t001 |  |  |  |  |  |  |  |  |  |  |  |  |
| 0 | Vvi-Vitvi07g04696\_t001 |  |  |  |  |  |  |  |  |  |  |  |  |
| 0 | Vvi-Vitvi07g02642\_t001 |  |  |  |  |  |  |  |  |  |  |  |  |
| 0 | Vvi-Vitvi07g01662\_t001 |  |  |  |  |  |  |  |  |  |  |  |  |
| 0 | Vvi-Vitvi07g02643\_t001 |  |  |  |  |  |  |  |  |  |  |  |  |
| 0 | Vvi-Vitvi07g01663\_t001 |  |  |  |  |  |  |  |  |  |  |  |  |
| 0 | Vvi-Vitvi07g01665\_t001 |  |  |  |  |  |  |  |  |  |  |  |  |
| 0 | Vvi-Vitvi07g04697\_t001 |  |  |  |  |  |  |  |  |  |  |  |  |
| 0 | Vvi-Vitvi07g01667\_t001 |  |  |  |  |  |  |  |  |  |  |  |  |
| 0 | Vvi-Vitvi07g01669\_t001 |  |  |  |  |  |  |  |  |  |  |  |  |
| 0 | Vvi-Vitvi07g04698\_t001 |  |  |  |  |  |  |  |  |  |  |  |  |
| 0 | Vvi-Vitvi07g02644\_t001 |  |  |  |  |  |  |  |  |  |  |  |  |
| 0 | Vvi-Vitvi07g02645\_t001 |  |  |  |  |  |  |  |  |  |  |  |  |
| 0 | Vvi-Vitvi07g04699\_t001 |  |  |  |  |  |  |  |  |  |  |  |  |
| 0 | Vvi-Vitvi07g02647\_t001 |  |  |  |  |  |  |  |  |  |  |  |  |
| 0 | Vvi-Vitvi07g01670\_t001 |  |  |  |  |  |  |  |  |  |  |  |  |
| 0 | Vvi-Vitvi07g02648\_t001 |  |  |  |  |  |  |  |  |  |  |  |  |
| 0 | Vvi-Vitvi07g02649\_t001 |  |  |  |  |  |  |  |  |  |  |  |  |
| 0 | Vvi-Vitvi07g04700\_t001 |  |  |  |  |  |  |  |  |  |  |  |  |
| 0 | Vvi-Vitvi07g01671\_t001 |  |  |  |  |  |  |  |  |  |  |  |  |
| 0 | Vvi-Vitvi07g02650\_t001 |  |  |  |  |  |  |  |  |  |  |  |  |
| 0 | Vvi-Vitvi07g01672\_t001 |  |  |  |  |  |  |  |  |  |  |  |  |
| 0 | Vvi-Vitvi07g01673\_t001 |  |  |  |  |  |  |  |  |  |  |  |  |
| 0 | Vvi-Vitvi07g04701\_t001 |  |  |  |  |  |  |  |  |  |  |  |  |
| 0 | Vvi-Vitvi07g01674\_t001 |  |  |  |  |  |  |  |  |  |  |  |  |
| 0 | Vvi-Vitvi07g01676\_t001 |  |  |  |  |  |  |  |  |  |  |  |  |
| 0 | Vvi-Vitvi07g04702\_t001 |  |  |  |  |  |  |  |  |  |  |  |  |
| 0 | Vvi-Vitvi07g04703\_t001 |  |  |  |  |  |  |  |  |  |  |  |  |
| 0 | Vvi-Vitvi07g01677\_t001 |  |  |  |  |  |  |  |  |  |  |  |  |
| 0 | Vvi-Vitvi07g01678\_t001 |  |  |  |  |  |  |  |  |  |  |  |  |
| 0 | Vvi-Vitvi07g01679\_t001 |  |  |  |  |  |  |  |  |  |  |  |  |
| 0 | Vvi-Vitvi07g04704\_t001 |  |  |  |  |  |  |  |  |  |  |  |  |
| 0 | Vvi-Vitvi07g01681\_t001 |  |  |  |  |  |  |  |  |  |  |  |  |
| 0 | Vvi-Vitvi07g01682\_t002 |  |  |  |  |  |  |  |  |  |  |  |  |
| 0 | Vvi-Vitvi07g01683\_t001 |  |  |  |  |  |  |  |  |  |  |  |  |
| 0 | Vvi-Vitvi07g01684\_t001 |  |  |  |  |  |  |  |  |  |  |  |  |
| 0 | Vvi-Vitvi07g01685\_t001 |  |  |  |  |  |  |  |  |  |  |  |  |
| 0 | Vvi-Vitvi07g01686\_t001 |  |  |  |  |  |  |  |  |  |  |  |  |
| 0 | Vvi-Vitvi07g04705\_t001 |  |  |  |  |  |  |  |  |  |  |  |  |
| 0 | Vvi-Vitvi07g01688\_t001 |  |  |  |  |  |  |  |  |  |  |  |  |
| 0 | Vvi-Vitvi07g01689\_t001 |  |  |  |  |  |  |  |  |  |  |  |  |
| 0 | Vvi-Vitvi07g04706\_t001 |  |  |  |  |  |  |  |  |  |  |  |  |
| 0 | Vvi-Vitvi07g01690\_t001 |  |  |  |  |  |  |  |  |  |  |  |  |
| 0 | Vvi-Vitvi07g01692\_t001 |  |  |  |  |  |  |  |  |  |  |  |  |
| 0 | Vvi-Vitvi07g01694\_t001 |  |  |  |  |  |  |  |  |  |  |  |  |
| 0 | Vvi-Vitvi07g01695\_t001 |  |  |  |  |  |  |  |  |  |  |  |  |
| 0 | Vvi-Vitvi07g01696\_t002 |  |  |  |  |  |  |  |  |  |  |  |  |
| 0 | Vvi-Vitvi07g01697\_t001 |  |  |  |  |  |  |  |  |  |  |  |  |
| 0 | Vvi-Vitvi07g01698\_t001 |  |  |  |  |  |  |  |  |  |  |  |  |
| 0 | Vvi-Vitvi07g01699\_t002 |  |  |  |  |  |  |  |  |  |  |  |  |
| 0 | Vvi-Vitvi07g01700\_t001 |  |  |  |  |  |  |  |  |  |  |  |  |
| 0 | Vvi-Vitvi07g01701\_t002 |  |  |  |  |  |  |  |  |  |  |  |  |
| 0 | Vvi-Vitvi07g01702\_t001 |  |  |  |  |  |  |  |  |  |  |  |  |
| 0 | Vvi-Vitvi07g01703\_t001 |  |  |  |  |  |  |  |  |  |  |  |  |
| 0 | Vvi-Vitvi07g01705\_t001 |  |  |  |  |  |  |  |  |  |  |  |  |
| 0 | Vvi-Vitvi07g01706\_t001 |  |  |  |  |  |  |  |  |  |  |  |  |
| 0 | Vvi-Vitvi07g01707\_t002 |  |  |  |  |  |  |  |  |  |  |  |  |
| 0 | Vvi-Vitvi07g01708\_t001 |  |  |  |  |  |  |  |  |  |  |  |  |
| 0 | Vvi-Vitvi07g01709\_t001 |  |  |  |  |  |  |  |  |  |  |  |  |
| 0 | Vvi-Vitvi07g01710\_t001 |  |  |  |  |  |  |  |  |  |  |  |  |
| 0 | Vvi-Vitvi07g04707\_t001 |  |  |  |  |  |  |  |  |  |  |  |  |
| 0 | Vvi-Vitvi07g04708\_t001 |  |  |  |  |  |  |  |  |  |  |  |  |
| 0 | Vvi-Vitvi07g01711\_t001 |  |  |  |  |  |  |  |  |  |  |  |  |
| 0 | Vvi-Vitvi07g04709\_t001 |  |  |  |  |  |  |  |  |  |  |  |  |
| 0 | Vvi-Vitvi07g01713\_t001 |  |  |  |  |  |  |  |  |  |  |  |  |
| 0 | Vvi-Vitvi07g01714\_t001 |  |  |  |  |  |  |  |  |  |  |  |  |
| 0 | Vvi-Vitvi07g04710\_t001 |  |  |  |  |  |  |  |  |  |  |  |  |
| 0 | Vvi-Vitvi07g01718\_t001 |  |  |  |  |  |  |  |  |  |  |  |  |
| 0 | Vvi-Vitvi07g01719\_t001 |  |  |  |  |  |  |  |  |  |  |  |  |
| 0 | Vvi-Vitvi07g04711\_t001 |  |  |  |  |  |  |  |  |  |  |  |  |
| 0 | Vvi-Vitvi07g04712\_t001 |  |  |  |  |  |  |  |  |  |  |  |  |
| 0 | Vvi-Vitvi07g01721\_t001 |  |  |  |  |  |  |  |  |  |  |  |  |
| 0 | Vvi-Vitvi07g01722\_t001 |  |  |  |  |  |  |  |  |  |  |  |  |
| 0 | Vvi-Vitvi07g02656\_t001 |  |  |  |  |  |  |  |  |  |  |  |  |
| 0 | Vvi-Vitvi07g01723\_t001 |  |  |  |  |  |  |  |  |  |  |  |  |
| 0 | Vvi-Vitvi07g01724\_t001 |  |  |  |  |  |  |  |  |  |  |  |  |
| 0 | Vvi-Vitvi07g02658\_t001 |  |  |  |  |  |  |  |  |  |  |  |  |
| 0 | Vvi-Vitvi07g04713\_t001 |  |  |  |  |  |  |  |  |  |  |  |  |
| 0 | Vvi-Vitvi07g02660\_t001 |  |  |  |  |  |  |  |  |  |  |  |  |
| 0 | Vvi-Vitvi07g02661\_t001 |  |  |  |  |  |  |  |  |  |  |  |  |
| 0 | Vvi-Vitvi07g04714\_t001 |  |  |  |  |  |  |  |  |  |  |  |  |
| 0 | Vvi-Vitvi07g04715\_t001 |  |  |  |  |  |  |  |  |  |  |  |  |
| 0 | Vvi-Vitvi07g02662\_t001 |  |  |  |  |  |  |  |  |  |  |  |  |
| 0 | Vvi-Vitvi07g01728\_t001 |  |  |  |  |  |  |  |  |  |  |  |  |
| 0 | Vvi-Vitvi07g01729\_t001 |  |  |  |  |  |  |  |  |  |  |  |  |
| 1 | Vvi-Vitvi07g01730\_t002 |  | Atr-ERN08694 |  |  |  |  |  |  |  |  |  |  |
| 1 | Vvi-Vitvi07g01731\_t001 |  | Atr-ERN08693 |  |  |  |  |  |  |  |  |  |  |
| 1 | Vvi-Vitvi07g01732\_t001 |  | \| \| |  |  |  |  |  |  |  |  |  |  |
| 1 | Vvi-Vitvi07g01733\_t001 |  | Atr-ERN08691 |  |  |  |  |  |  |  |  |  |  |
| 1 | Vvi-Vitvi07g04716\_t001 |  | \| \| |  |  |  |  |  |  |  |  |  |  |
| 1 | Vvi-Vitvi07g01734\_t001 |  | Atr-ERN08685 |  |  |  |  |  |  |  |  |  |  |
| 1 | Vvi-Vitvi07g01735\_t001 |  | \| \| |  |  |  |  |  |  |  |  |  |  |
| 1 | Vvi-Vitvi07g01736\_t001 |  | Atr-ERN08670 |  |  |  |  |  |  |  |  |  |  |
| 1 | Vvi-Vitvi07g01737\_t001 |  | \| \| |  |  |  |  |  |  |  |  |  |  |
| 1 | Vvi-Vitvi07g01739\_t001 |  | Atr-ERN08659 |  |  |  |  |  |  |  |  |  |  |
| 0 | Vvi-Vitvi07g01740\_t001 |  |  |  |  |  |  |  |  |  |  |  |  |
| 0 | Vvi-Vitvi07g02664\_t001 |  |  |  |  |  |  |  |  |  |  |  |  |
| 0 | Vvi-Vitvi07g02665\_t001 |  |  |  |  |  |  |  |  |  |  |  |  |
| 0 | Vvi-Vitvi07g01742\_t002 |  |  |  |  |  |  |  |  |  |  |  |  |
| 0 | Vvi-Vitvi07g01743\_t004 |  |  |  |  |  |  |  |  |  |  |  |  |
| 0 | Vvi-Vitvi07g01744\_t001 |  |  |  |  |  |  |  |  |  |  |  |  |
| 0 | Vvi-Vitvi07g01745\_t001 |  |  |  |  |  |  |  |  |  |  |  |  |
| 0 | Vvi-Vitvi07g01746\_t001 |  |  |  |  |  |  |  |  |  |  |  |  |
| 0 | Vvi-Vitvi07g01747\_t001 |  |  |  |  |  |  |  |  |  |  |  |  |
| 0 | Vvi-Vitvi07g02666\_t001 |  |  |  |  |  |  |  |  |  |  |  |  |
| 0 | Vvi-Vitvi07g01748\_t001 |  |  |  |  |  |  |  |  |  |  |  |  |
| 0 | Vvi-Vitvi07g01749\_t001 |  |  |  |  |  |  |  |  |  |  |  |  |
| 0 | Vvi-Vitvi07g01750\_t001 |  |  |  |  |  |  |  |  |  |  |  |  |
| 0 | Vvi-Vitvi07g01751\_t001 |  |  |  |  |  |  |  |  |  |  |  |  |
| 0 | Vvi-Vitvi07g01752\_t001 |  |  |  |  |  |  |  |  |  |  |  |  |
| 0 | Vvi-Vitvi07g01753\_t001 |  |  |  |  |  |  |  |  |  |  |  |  |
| 0 | Vvi-Vitvi07g04717\_t001 |  |  |  |  |  |  |  |  |  |  |  |  |
| 0 | Vvi-Vitvi07g04718\_t001 |  |  |  |  |  |  |  |  |  |  |  |  |
| 0 | Vvi-Vitvi07g04719\_t001 |  |  |  |  |  |  |  |  |  |  |  |  |
| 0 | Vvi-Vitvi07g01755\_t001 |  |  |  |  |  |  |  |  |  |  |  |  |
| 0 | Vvi-Vitvi07g02069\_t001 |  |  |  |  |  |  |  |  |  |  |  |  |
| 0 | Vvi-Vitvi07g01756\_t001 |  |  |  |  |  |  |  |  |  |  |  |  |
| 0 | Vvi-Vitvi07g04720\_t001 |  |  |  |  |  |  |  |  |  |  |  |  |
| 0 | Vvi-Vitvi07g01757\_t001 |  |  |  |  |  |  |  |  |  |  |  |  |
| 0 | Vvi-Vitvi07g01759\_t001 |  |  |  |  |  |  |  |  |  |  |  |  |
| 1 | Vvi-Vitvi07g01760\_t001 |  | Atr-ERM97154 |  |  |  |  |  |  |  |  |  |  |
| 1 | Vvi-Vitvi07g01761\_t001 |  | Atr-ERM97149 |  |  |  |  |  |  |  |  |  |  |
| 1 | Vvi-Vitvi07g01762\_t001 |  | \| \| |  |  |  |  |  |  |  |  |  |  |
| 1 | Vvi-Vitvi07g01763\_t001 |  | Atr-ERM97137 |  |  |  |  |  |  |  |  |  |  |
| 1 | Vvi-Vitvi07g02667\_t002 |  | Atr-ERM97131 |  |  |  |  |  |  |  |  |  |  |
| 1 | Vvi-Vitvi07g01765\_t001 |  | Atr-ERM97124 |  |  |  |  |  |  |  |  |  |  |
| 1 | Vvi-Vitvi07g01766\_t001 |  | Atr-ERM97110 |  |  |  |  |  |  |  |  |  |  |
| 1 | Vvi-Vitvi07g01767\_t001 |  | \| \| |  |  |  |  |  |  |  |  |  |  |
| 1 | Vvi-Vitvi07g01768\_t001 |  | Atr-ERM97097 |  |  |  |  |  |  |  |  |  |  |
| 0 | Vvi-Vitvi07g01769\_t001 |  |  |  |  |  |  |  |  |  |  |  |  |
| 0 | Vvi-Vitvi07g01770\_t001 |  |  |  |  |  |  |  |  |  |  |  |  |
| 0 | Vvi-Vitvi07g01772\_t001 |  |  |  |  |  |  |  |  |  |  |  |  |
| 0 | Vvi-Vitvi07g04721\_t001 |  |  |  |  |  |  |  |  |  |  |  |  |
| 0 | Vvi-Vitvi07g04722\_t001 |  |  |  |  |  |  |  |  |  |  |  |  |
| 0 | Vvi-Vitvi07g01773\_t001 |  |  |  |  |  |  |  |  |  |  |  |  |
| 0 | Vvi-Vitvi07g04723\_t001 |  |  |  |  |  |  |  |  |  |  |  |  |
| 0 | Vvi-Vitvi07g02668\_t001 |  |  |  |  |  |  |  |  |  |  |  |  |
| 0 | Vvi-Vitvi07g02670\_t001 |  |  |  |  |  |  |  |  |  |  |  |  |
| 0 | Vvi-Vitvi07g04724\_t001 |  |  |  |  |  |  |  |  |  |  |  |  |
| 0 | Vvi-Vitvi07g04725\_t001 |  |  |  |  |  |  |  |  |  |  |  |  |
| 0 | Vvi-Vitvi07g04726\_t001 |  |  |  |  |  |  |  |  |  |  |  |  |
| 0 | Vvi-Vitvi07g04727\_t001 |  |  |  |  |  |  |  |  |  |  |  |  |
| 1 | Vvi-Vitvi07g01777\_t001 |  | Atr-ERM94221 |  |  |  |  |  |  |  |  |  |  |
| 1 | Vvi-Vitvi07g01778\_t001 |  | Atr-ERM94223 |  |  |  |  |  |  |  |  |  |  |
| 1 | Vvi-Vitvi07g01779\_t001 |  | Atr-ERM94226 |  |  |  |  |  |  |  |  |  |  |
| 1 | Vvi-Vitvi07g01780\_t001 |  | \| \| |  |  |  |  |  |  |  |  |  |  |
| 1 | Vvi-Vitvi07g01783\_t002 |  | Atr-ERM94229 |  |  |  |  |  |  |  |  |  |  |
| 1 | Vvi-Vitvi07g02672\_t001 |  | \| \| |  |  |  |  |  |  |  |  |  |  |
| 1 | Vvi-Vitvi07g02674\_t001 |  | Atr-ERM94236 |  |  |  |  |  |  |  |  |  |  |
| 1 | Vvi-Vitvi07g02675\_t001 |  | \| \| |  |  |  |  |  |  |  |  |  |  |
| 1 | Vvi-Vitvi07g02676\_t001 |  | \| \| |  |  |  |  |  |  |  |  |  |  |
| 1 | Vvi-Vitvi07g04728\_t001 |  | \| \| |  |  |  |  |  |  |  |  |  |  |
| 1 | Vvi-Vitvi07g01784\_t001 |  | \| \| |  |  |  |  |  |  |  |  |  |  |
| 1 | Vvi-Vitvi07g01787\_t001 |  | \| \| |  |  |  |  |  |  |  |  |  |  |
| 1 | Vvi-Vitvi07g04729\_t001 |  | \| \| |  |  |  |  |  |  |  |  |  |  |
| 1 | Vvi-Vitvi07g01789\_t001 |  | \| \| |  |  |  |  |  |  |  |  |  |  |
| 1 | Vvi-Vitvi07g04730\_t001 |  | \| \| |  |  |  |  |  |  |  |  |  |  |
| 1 | Vvi-Vitvi07g01790\_t001 |  | \| \| |  |  |  |  |  |  |  |  |  |  |
| 1 | Vvi-Vitvi07g01791\_t001 |  | \| \| |  |  |  |  |  |  |  |  |  |  |
| 1 | Vvi-Vitvi07g04731\_t001 |  | \| \| |  |  |  |  |  |  |  |  |  |  |
| 1 | Vvi-Vitvi07g01792\_t001 |  | Atr-ERM94241 |  |  |  |  |  |  |  |  |  |  |
| 1 | Vvi-Vitvi07g04732\_t003 |  | Atr-ERM94242 |  |  |  |  |  |  |  |  |  |  |
| 1 | Vvi-Vitvi07g01794\_t001 |  | Atr-ERM94243 |  |  |  |  |  |  |  |  |  |  |
| 1 | Vvi-Vitvi07g01795\_t001 |  | Atr-ERM94244 |  |  |  |  |  |  |  |  |  |  |
| 1 | Vvi-Vitvi07g01796\_t001 |  | Atr-ERM94246 |  |  |  |  |  |  |  |  |  |  |
| 1 | Vvi-Vitvi07g01797\_t001 |  | \| \| |  |  |  |  |  |  |  |  |  |  |
| 1 | Vvi-Vitvi07g01798\_t001 |  | Atr-ERM94247 |  |  |  |  |  |  |  |  |  |  |
| 1 | Vvi-Vitvi07g01800\_t001 |  | Atr-ERM94248 |  |  |  |  |  |  |  |  |  |  |
| 1 | Vvi-Vitvi07g01802\_t001 |  | Atr-ERM94250 |  |  |  |  |  |  |  |  |  |  |
| 1 | Vvi-Vitvi07g01803\_t002 |  | Atr-ERM94251 |  |  |  |  |  |  |  |  |  |  |
| 1 | Vvi-Vitvi07g01804\_t001 |  | Atr-ERM94253 |  |  |  |  |  |  |  |  |  |  |
| 1 | Vvi-Vitvi07g02679\_t001 |  | Atr-ERM94266 |  |  |  |  |  |  |  |  |  |  |
| 1 | Vvi-Vitvi07g02680\_t001 |  | \| \| |  |  |  |  |  |  |  |  |  |  |
| 1 | Vvi-Vitvi07g02681\_t001 |  | \| \| |  |  |  |  |  |  |  |  |  |  |
| 1 | Vvi-Vitvi07g01805\_t001 |  | \| \| |  |  |  |  |  |  |  |  |  |  |
| 1 | Vvi-Vitvi07g02682\_t001 |  | \| \| |  |  |  |  |  |  |  |  |  |  |
| 1 | Vvi-Vitvi07g01806\_t001 |  | Atr-ERM94275 |  |  |  |  |  |  |  |  |  |  |
| 1 | Vvi-Vitvi07g02683\_t001 |  | Atr-ERM94280 |  |  |  |  |  |  |  |  |  |  |
| 1 | Vvi-Vitvi07g02684\_t001 |  | \| \| |  |  |  |  |  |  |  |  |  |  |
| 1 | Vvi-Vitvi07g04733\_t001 |  | \| \| |  |  |  |  |  |  |  |  |  |  |
| 1 | Vvi-Vitvi07g01807\_t001 |  | Atr-ERM94291 |  |  |  |  |  |  |  |  |  |  |
| 1 | Vvi-Vitvi07g01808\_t001 |  | Atr-ERM94293 |  |  |  |  |  |  |  |  |  |  |
| 1 | Vvi-Vitvi07g01809\_t001 |  | \| \| |  |  |  |  |  |  |  |  |  |  |
| 1 | Vvi-Vitvi07g01810\_t001 |  | \| \| |  |  |  |  |  |  |  |  |  |  |
| 1 | Vvi-Vitvi07g01811\_t001 |  | \| \| |  |  |  |  |  |  |  |  |  |  |
| 1 | Vvi-Vitvi07g01813\_t001 |  | \| \| |  |  |  |  |  |  |  |  |  |  |
| 1 | Vvi-Vitvi07g02686\_t001 |  | \| \| |  |  |  |  |  |  |  |  |  |  |
| 1 | Vvi-Vitvi07g01814\_t001 |  | \| \| |  |  |  |  |  |  |  |  |  |  |
| 1 | Vvi-Vitvi07g01816\_t001 |  | \| \| |  |  |  |  |  |  |  |  |  |  |
| 1 | Vvi-Vitvi07g01817\_t001 |  | \| \| |  |  |  |  |  |  |  |  |  |  |
| 1 | Vvi-Vitvi07g01818\_t001 |  | Atr-ERM94317 |  |  |  |  |  |  |  |  |  |  |
| 1 | Vvi-Vitvi07g04734\_t001 |  | \| \| |  |  |  |  |  |  |  |  |  |  |
| 1 | Vvi-Vitvi07g01821\_t001 |  | \| \| |  |  |  |  |  |  |  |  |  |  |
| 1 | Vvi-Vitvi07g01822\_t001 |  | \| \| |  |  |  |  |  |  |  |  |  |  |
| 1 | Vvi-Vitvi07g01823\_t001 |  | Atr-ERM94322 |  |  |  |  |  |  |  |  |  |  |
| 1 | Vvi-Vitvi07g01824\_t001 |  | \| \| |  |  |  |  |  |  |  |  |  |  |
| 1 | Vvi-Vitvi07g01825\_t001 |  | Atr-ERM94326 |  |  |  |  |  |  |  |  |  |  |
| 1 | Vvi-Vitvi07g01826\_t001 |  | Atr-ERM94329 |  |  |  |  |  |  |  |  |  |  |
| 1 | Vvi-Vitvi07g04735\_t001 |  | \| \| |  |  |  |  |  |  |  |  |  |  |
| 1 | Vvi-Vitvi07g01827\_t001 |  | Atr-ERM94330 |  |  |  |  |  |  |  |  |  |  |
| 1 | Vvi-Vitvi07g02687\_t001 |  | \| \| |  |  |  |  |  |  |  |  |  |  |
| 1 | Vvi-Vitvi07g01828\_t001 |  | Atr-ERM94332 |  |  |  |  |  |  |  |  |  |  |
| 1 | Vvi-Vitvi07g02688\_t001 |  | \| \| |  |  |  |  |  |  |  |  |  |  |
| 1 | Vvi-Vitvi07g01829\_t001 |  | Atr-ERM94334 |  |  |  |  |  |  |  |  |  |  |
| 1 | Vvi-Vitvi07g01830\_t001 |  | Atr-ERM94335 |  |  |  |  |  |  |  |  |  |  |
| 1 | Vvi-Vitvi07g01831\_t002 |  | Atr-ERM94336 |  |  |  |  |  |  |  |  |  |  |
| 1 | Vvi-Vitvi07g01832\_t001 |  | Atr-ERM94338 |  |  |  |  |  |  |  |  |  |  |
| 1 | Vvi-Vitvi07g04736\_t001 |  | \| \| |  |  |  |  |  |  |  |  |  |  |
| 1 | Vvi-Vitvi07g04737\_t001 |  | \| \| |  |  |  |  |  |  |  |  |  |  |
| 1 | Vvi-Vitvi07g02689\_t001 |  | Atr-ERM94345 |  |  |  |  |  |  |  |  |  |  |
| 1 | Vvi-Vitvi07g01835\_t001 |  | Atr-ERM94353 |  |  |  |  |  |  |  |  |  |  |
| 1 | Vvi-Vitvi07g01836\_t001 |  | Atr-ERM94359 |  |  |  |  |  |  |  |  |  |  |
| 1 | Vvi-Vitvi07g01837\_t001 |  | \| \| |  |  |  |  |  |  |  |  |  |  |
| 1 | Vvi-Vitvi07g01838\_t001 |  | Atr-ERM94360 |  |  |  |  |  |  |  |  |  |  |
| 1 | Vvi-Vitvi07g04738\_t001 |  | \| \| |  |  |  |  |  |  |  |  |  |  |
| 1 | Vvi-Vitvi07g02691\_t001 |  | Atr-ERM94361 |  |  |  |  |  |  |  |  |  |  |
| 1 | Vvi-Vitvi07g01840\_t001 |  | Atr-ERM94363 |  |  |  |  |  |  |  |  |  |  |
| 0 | Vvi-Vitvi07g01841\_t001.2.6037826f |  |  |  |  |  |  |  |  |  |  |  |  |
| 0 | Vvi-Vitvi07g01842\_t001 |  |  |  |  |  |  |  |  |  |  |  |  |
| 0 | Vvi-Vitvi07g02692\_t001 |  |  |  |  |  |  |  |  |  |  |  |  |
| 0 | Vvi-Vitvi07g01843\_t001 |  |  |  |  |  |  |  |  |  |  |  |  |
| 0 | Vvi-Vitvi07g04739\_t001 |  |  |  |  |  |  |  |  |  |  |  |  |
| 0 | Vvi-Vitvi07g01844\_t001 |  |  |  |  |  |  |  |  |  |  |  |  |
| 0 | Vvi-Vitvi07g01845\_t001 |  |  |  |  |  |  |  |  |  |  |  |  |
| 1 | Vvi-Vitvi07g01846\_t001 |  | Atr-ERN03838 |  |  |  |  |  |  |  |  |  |  |
| 1 | Vvi-Vitvi07g01847\_t001 |  | Atr-ERN03839 |  |  |  |  |  |  |  |  |  |  |
| 1 | Vvi-Vitvi07g01848\_t001 |  | Atr-ERN03840 |  |  |  |  |  |  |  |  |  |  |
| 1 | Vvi-Vitvi07g01849\_t001 |  | Atr-ERN03842 |  |  |  |  |  |  |  |  |  |  |
| 1 | Vvi-Vitvi07g01850\_t001 |  | \| \| |  |  |  |  |  |  |  |  |  |  |
| 1 | Vvi-Vitvi07g01851\_t001 |  | Atr-ERN03844 |  |  |  |  |  |  |  |  |  |  |
| 1 | Vvi-Vitvi07g01852\_t001 |  | Atr-ERN03865 |  |  |  |  |  |  |  |  |  |  |
| 1 | Vvi-Vitvi07g01853\_t001 |  | \| \| |  |  |  |  |  |  |  |  |  |  |
| 1 | Vvi-Vitvi07g01855\_t001 |  | \| \| |  |  |  |  |  |  |  |  |  |  |
| 1 | Vvi-Vitvi07g01856\_t001 |  | \| \| |  |  |  |  |  |  |  |  |  |  |
| 1 | Vvi-Vitvi07g01857\_t001 |  | Atr-ERN03873 |  |  |  |  |  |  |  |  |  |  |
| 1 | Vvi-Vitvi07g01858\_t002 |  | Atr-ERN03875 |  |  |  |  |  |  |  |  |  |  |
| 1 | Vvi-Vitvi07g01859\_t001 |  | Atr-ERN03878 |  |  |  |  |  |  |  |  |  |  |
| 1 | Vvi-Vitvi07g01860\_t001 |  | Atr-ERN03881 |  |  |  |  |  |  |  |  |  |  |
| 1 | Vvi-Vitvi07g01861\_t001 |  | \| \| |  |  |  |  |  |  |  |  |  |  |
| 1 | Vvi-Vitvi07g01862\_t001 |  | \| \| |  |  |  |  |  |  |  |  |  |  |
| 1 | Vvi-Vitvi07g02693\_t001 |  | \| \| |  |  |  |  |  |  |  |  |  |  |
| 1 | Vvi-Vitvi07g01863\_t001 |  | \| \| |  |  |  |  |  |  |  |  |  |  |
| 1 | Vvi-Vitvi07g01864\_t001 |  | \| \| |  |  |  |  |  |  |  |  |  |  |
| 2 | Vvi-Vitvi07g01865\_t001 |  | \| \| |  | Atr-ERN08699 |  |  |  |  |  |  |  |  |
| 2 | Vvi-Vitvi07g01866\_t001 |  | \| \| |  | \| \| |  |  |  |  |  |  |  |  |
| 2 | Vvi-Vitvi07g01867\_t001 |  | \| \| |  | \| \| |  |  |  |  |  |  |  |  |
| 2 | Vvi-Vitvi07g04740\_t001 |  | \| \| |  | \| \| |  |  |  |  |  |  |  |  |
| 2 | Vvi-Vitvi07g01869\_t001 |  | \| \| |  | \| \| |  |  |  |  |  |  |  |  |
| 2 | Vvi-Vitvi07g01870\_t001 |  | \| \| |  | \| \| |  |  |  |  |  |  |  |  |
| 2 | Vvi-Vitvi07g02694\_t001 |  | Atr-ERN03886 |  | \| \| |  |  |  |  |  |  |  |  |
| 2 | Vvi-Vitvi07g04741\_t001 |  | \| \| |  | \| \| |  |  |  |  |  |  |  |  |
| 2 | Vvi-Vitvi07g04742\_t001 |  | \| \| |  | \| \| |  |  |  |  |  |  |  |  |
| 2 | Vvi-Vitvi07g01871\_t001 |  | \| \| |  | Atr-ERN08705 |  |  |  |  |  |  |  |  |
| 2 | Vvi-Vitvi07g01872\_t001 |  | \| \| |  | \| \| |  |  |  |  |  |  |  |  |
| 2 | Vvi-Vitvi07g01874\_t001 |  | \| \| |  | \| \| |  |  |  |  |  |  |  |  |
| 2 | Vvi-Vitvi07g01875\_t001 |  | \| \| |  | Atr-ERN08710 |  |  |  |  |  |  |  |  |
| 2 | Vvi-Vitvi07g02696\_t001 |  | \| \| |  | \| \| |  |  |  |  |  |  |  |  |
| 2 | Vvi-Vitvi07g04743\_t001 |  | \| \| |  | \| \| |  |  |  |  |  |  |  |  |
| 2 | Vvi-Vitvi07g01876\_t001 |  | \| \| |  | Atr-ERN08711 |  |  |  |  |  |  |  |  |
| 2 | Vvi-Vitvi07g01877\_t001 |  | \| \| |  | Atr-ERN08713 |  |  |  |  |  |  |  |  |
| 2 | Vvi-Vitvi07g01878\_t001 |  | \| \| |  | \| \| |  |  |  |  |  |  |  |  |
| 2 | Vvi-Vitvi07g01879\_t001 |  | \| \| |  | Atr-ERN08718 |  |  |  |  |  |  |  |  |
| 2 | Vvi-Vitvi07g01880\_t001 |  | \| \| |  | \| \| |  |  |  |  |  |  |  |  |
| 2 | Vvi-Vitvi07g02697\_t001 |  | \| \| |  | \| \| |  |  |  |  |  |  |  |  |
| 2 | Vvi-Vitvi07g01881\_t001 |  | \| \| |  | Atr-ERN08733 |  |  |  |  |  |  |  |  |
| 2 | Vvi-Vitvi07g01882\_t001 |  | \| \| |  | Atr-ERN08734 |  |  |  |  |  |  |  |  |
| 1 | Vvi-Vitvi07g01883\_t001 |  | \| \| |  |  |  |  |  |  |  |  |  |  |
| 1 | Vvi-Vitvi07g02698\_t001 |  | \| \| |  |  |  |  |  |  |  |  |  |  |
| 1 | Vvi-Vitvi07g01884\_t004 |  | \| \| |  |  |  |  |  |  |  |  |  |  |
| 1 | Vvi-Vitvi07g01885\_t001 |  | \| \| |  |  |  |  |  |  |  |  |  |  |
| 2 | Vvi-Vitvi07g01886\_t002 |  | Atr-ERN03909 |  | Atr-ERN17185 |  |  |  |  |  |  |  |  |
| 1 | Vvi-Vitvi07g01887\_t001 |  |  |  | Atr-ERN17182 |  |  |  |  |  |  |  |  |
| 1 | Vvi-Vitvi07g01888\_t001 |  |  |  | \| \| |  |  |  |  |  |  |  |  |
| 1 | Vvi-Vitvi07g01889\_t001 |  |  |  | Atr-ERN17166 |  |  |  |  |  |  |  |  |
| 1 | Vvi-Vitvi07g04744\_t001 |  |  |  | \| \| |  |  |  |  |  |  |  |  |
| 1 | Vvi-Vitvi07g01890\_t001 |  |  |  | Atr-ERN17159 |  |  |  |  |  |  |  |  |
| 1 | Vvi-Vitvi07g01891\_t001 |  |  |  | Atr-ERN17151 |  |  |  |  |  |  |  |  |
| 1 | Vvi-Vitvi07g01892\_t001 |  |  |  | Atr-ERN17148 |  |  |  |  |  |  |  |  |
| 1 | Vvi-Vitvi07g01893\_t001 |  |  |  | Atr-ERN17141 |  |  |  |  |  |  |  |  |
| 1 | Vvi-Vitvi07g01894\_t001 |  |  |  | \| \| |  |  |  |  |  |  |  |  |
| 1 | Vvi-Vitvi07g04745\_t001 |  |  |  | Atr-ERN17134 |  |  |  |  |  |  |  |  |
| 1 | Vvi-Vitvi07g01896\_t001 |  |  |  | \| \| |  |  |  |  |  |  |  |  |
| 1 | Vvi-Vitvi07g02702\_t001 |  |  |  | Atr-ERN17116 |  |  |  |  |  |  |  |  |
| 1 | Vvi-Vitvi07g01897\_t001 |  |  |  | Atr-ERN17114 |  |  |  |  |  |  |  |  |
| 1 | Vvi-Vitvi07g01898\_t001 |  |  |  | Atr-ERN17113 |  |  |  |  |  |  |  |  |
| 1 | Vvi-Vitvi07g01899\_t001 |  |  |  | \| \| |  |  |  |  |  |  |  |  |
| 1 | Vvi-Vitvi07g01900\_t001 |  |  |  | Atr-ERN17112 |  |  |  |  |  |  |  |  |
| 1 | Vvi-Vitvi07g01901\_t001 |  |  |  | Atr-ERN17111 |  |  |  |  |  |  |  |  |
| 0 | Vvi-Vitvi07g01902\_t001 |  |  |  |  |  |  |  |  |  |  |  |  |
| 0 | Vvi-Vitvi07g04746\_t001 |  |  |  |  |  |  |  |  |  |  |  |  |
| 0 | Vvi-Vitvi07g01903\_t001 |  |  |  |  |  |  |  |  |  |  |  |  |
| 0 | Vvi-Vitvi07g04747\_t001 |  |  |  |  |  |  |  |  |  |  |  |  |
| 0 | Vvi-Vitvi07g01905\_t001 |  |  |  |  |  |  |  |  |  |  |  |  |
| 0 | Vvi-Vitvi07g01906\_t001 |  |  |  |  |  |  |  |  |  |  |  |  |
| 0 | Vvi-Vitvi07g02703\_t001 |  |  |  |  |  |  |  |  |  |  |  |  |
| 0 | Vvi-Vitvi07g04748\_t001 |  |  |  |  |  |  |  |  |  |  |  |  |
| 0 | Vvi-Vitvi07g04749\_t001 |  |  |  |  |  |  |  |  |  |  |  |  |
| 0 | Vvi-Vitvi07g01908\_t001 |  |  |  |  |  |  |  |  |  |  |  |  |
| 0 | Vvi-Vitvi07g01909\_t001 |  |  |  |  |  |  |  |  |  |  |  |  |
| 0 | Vvi-Vitvi07g01910\_t002 |  |  |  |  |  |  |  |  |  |  |  |  |
| 0 | Vvi-Vitvi07g01911\_t001 |  |  |  |  |  |  |  |  |  |  |  |  |
| 0 | Vvi-Vitvi07g04750\_t001 |  |  |  |  |  |  |  |  |  |  |  |  |
| 0 | Vvi-Vitvi07g02707\_t001 |  |  |  |  |  |  |  |  |  |  |  |  |
| 0 | Vvi-Vitvi07g01913\_t001 |  |  |  |  |  |  |  |  |  |  |  |  |
| 0 | Vvi-Vitvi07g04751\_t001 |  |  |  |  |  |  |  |  |  |  |  |  |
| 0 | Vvi-Vitvi07g01916\_t001 |  |  |  |  |  |  |  |  |  |  |  |  |
| 0 | Vvi-Vitvi07g01917\_t001 |  |  |  |  |  |  |  |  |  |  |  |  |
| 0 | Vvi-Vitvi07g04752\_t001 |  |  |  |  |  |  |  |  |  |  |  |  |
| 0 | Vvi-Vitvi07g02708\_t001 |  |  |  |  |  |  |  |  |  |  |  |  |
| 0 | Vvi-Vitvi07g01918\_t001 |  |  |  |  |  |  |  |  |  |  |  |  |
| 0 | Vvi-Vitvi07g01919\_t001.1.6037826f |  |  |  |  |  |  |  |  |  |  |  |  |
| 0 | Vvi-Vitvi07g01920\_t001 |  |  |  |  |  |  |  |  |  |  |  |  |
| 0 | Vvi-Vitvi07g04753\_t001 |  |  |  |  |  |  |  |  |  |  |  |  |
| 0 | Vvi-Vitvi07g04754\_t001 |  |  |  |  |  |  |  |  |  |  |  |  |
| 0 | Vvi-Vitvi07g04755\_t001 |  |  |  |  |  |  |  |  |  |  |  |  |
| 0 | Vvi-Vitvi07g01921\_t001 |  |  |  |  |  |  |  |  |  |  |  |  |
| 0 | Vvi-Vitvi07g01923\_t001 |  |  |  |  |  |  |  |  |  |  |  |  |
| 0 | Vvi-Vitvi07g01924\_t001 |  |  |  |  |  |  |  |  |  |  |  |  |
| 0 | Vvi-Vitvi07g01927\_t001 |  |  |  |  |  |  |  |  |  |  |  |  |
| 0 | Vvi-Vitvi07g01929\_t001 |  |  |  |  |  |  |  |  |  |  |  |  |
| 0 | Vvi-Vitvi07g01930\_t001 |  |  |  |  |  |  |  |  |  |  |  |  |
| 0 | Vvi-Vitvi07g04756\_t001 |  |  |  |  |  |  |  |  |  |  |  |  |
| 0 | Vvi-Vitvi07g01932\_t001 |  |  |  |  |  |  |  |  |  |  |  |  |
| 0 | Vvi-Vitvi07g01934\_t002 |  |  |  |  |  |  |  |  |  |  |  |  |
| 0 | Vvi-Vitvi07g01936\_t001 |  |  |  |  |  |  |  |  |  |  |  |  |
| 0 | Vvi-Vitvi07g01937\_t002 |  |  |  |  |  |  |  |  |  |  |  |  |
| 0 | Vvi-Vitvi07g01941\_t001 |  |  |  |  |  |  |  |  |  |  |  |  |
| 0 | Vvi-Vitvi07g01942\_t001 |  |  |  |  |  |  |  |  |  |  |  |  |
| 0 | Vvi-Vitvi07g01943\_t001 |  |  |  |  |  |  |  |  |  |  |  |  |
| 0 | Vvi-Vitvi07g01946\_t001 |  |  |  |  |  |  |  |  |  |  |  |  |
| 0 | Vvi-Vitvi07g04757\_t001 |  |  |  |  |  |  |  |  |  |  |  |  |
| 0 | Vvi-Vitvi07g01947\_t001 |  |  |  |  |  |  |  |  |  |  |  |  |
| 0 | Vvi-Vitvi07g01948\_t002 |  |  |  |  |  |  |  |  |  |  |  |  |
| 0 | Vvi-Vitvi07g01949\_t003 |  |  |  |  |  |  |  |  |  |  |  |  |
| 0 | Vvi-Vitvi07g01950\_t001 |  |  |  |  |  |  |  |  |  |  |  |  |
| 0 | Vvi-Vitvi07g01951\_t001 |  |  |  |  |  |  |  |  |  |  |  |  |
| 0 | Vvi-Vitvi07g01952\_t001 |  |  |  |  |  |  |  |  |  |  |  |  |
| 0 | Vvi-Vitvi07g01954\_t001 |  |  |  |  |  |  |  |  |  |  |  |  |
| 0 | Vvi-Vitvi07g04758\_t001 |  |  |  |  |  |  |  |  |  |  |  |  |
| 0 | Vvi-Vitvi07g01955\_t001 |  |  |  |  |  |  |  |  |  |  |  |  |
| 0 | Vvi-Vitvi07g01956\_t001 |  |  |  |  |  |  |  |  |  |  |  |  |
| 0 | Vvi-Vitvi07g02711\_t001 |  |  |  |  |  |  |  |  |  |  |  |  |
| 0 | Vvi-Vitvi07g04759\_t001 |  |  |  |  |  |  |  |  |  |  |  |  |
| 0 | Vvi-Vitvi07g01957\_t003 |  |  |  |  |  |  |  |  |  |  |  |  |
| 0 | Vvi-Vitvi07g01958\_t001 |  |  |  |  |  |  |  |  |  |  |  |  |
| 0 | Vvi-Vitvi07g04760\_t001 |  |  |  |  |  |  |  |  |  |  |  |  |
| 0 | Vvi-Vitvi07g01959\_t001 |  |  |  |  |  |  |  |  |  |  |  |  |
| 0 | Vvi-Vitvi07g01961\_t001 |  |  |  |  |  |  |  |  |  |  |  |  |
| 0 | Vvi-Vitvi07g04761\_t001 |  |  |  |  |  |  |  |  |  |  |  |  |
| 0 | Vvi-Vitvi07g01964\_t001 |  |  |  |  |  |  |  |  |  |  |  |  |
| 0 | Vvi-Vitvi07g01965\_t001 |  |  |  |  |  |  |  |  |  |  |  |  |
| 0 | Vvi-Vitvi07g04762\_t001 |  |  |  |  |  |  |  |  |  |  |  |  |
| 0 | Vvi-Vitvi07g01966\_t001 |  |  |  |  |  |  |  |  |  |  |  |  |
| 0 | Vvi-Vitvi07g04763\_t001 |  |  |  |  |  |  |  |  |  |  |  |  |
| 0 | Vvi-Vitvi07g02714\_t001 |  |  |  |  |  |  |  |  |  |  |  |  |
| 0 | Vvi-Vitvi07g01969\_t001 |  |  |  |  |  |  |  |  |  |  |  |  |
| 0 | Vvi-Vitvi07g01970\_t001 |  |  |  |  |  |  |  |  |  |  |  |  |
| 0 | Vvi-Vitvi07g01973\_t001 |  |  |  |  |  |  |  |  |  |  |  |  |
| 0 | Vvi-Vitvi07g01977\_t001 |  |  |  |  |  |  |  |  |  |  |  |  |
| 0 | Vvi-Vitvi07g01978\_t001 |  |  |  |  |  |  |  |  |  |  |  |  |
| 0 | Vvi-Vitvi07g04764\_t001 |  |  |  |  |  |  |  |  |  |  |  |  |
| 0 | Vvi-Vitvi07g01979\_t001 |  |  |  |  |  |  |  |  |  |  |  |  |
| 0 | Vvi-Vitvi07g01980\_t001 |  |  |  |  |  |  |  |  |  |  |  |  |
| 0 | Vvi-Vitvi07g01981\_t001 |  |  |  |  |  |  |  |  |  |  |  |  |
| 0 | Vvi-Vitvi07g01982\_t001 |  |  |  |  |  |  |  |  |  |  |  |  |
| 0 | Vvi-Vitvi07g01983\_t001 |  |  |  |  |  |  |  |  |  |  |  |  |
| 0 | Vvi-Vitvi07g01984\_t005 |  |  |  |  |  |  |  |  |  |  |  |  |
| 0 | Vvi-Vitvi07g01985\_t001 |  |  |  |  |  |  |  |  |  |  |  |  |
| 0 | Vvi-Vitvi07g01988\_t001 |  |  |  |  |  |  |  |  |  |  |  |  |
| 0 | Vvi-Vitvi07g01989\_t001 |  |  |  |  |  |  |  |  |  |  |  |  |
| 0 | Vvi-Vitvi07g01990\_t001 |  |  |  |  |  |  |  |  |  |  |  |  |
| 0 | Vvi-Vitvi07g02716\_t001 |  |  |  |  |  |  |  |  |  |  |  |  |
| 0 | Vvi-Vitvi07g02717\_t001 |  |  |  |  |  |  |  |  |  |  |  |  |
| 0 | Vvi-Vitvi07g01991\_t001 |  |  |  |  |  |  |  |  |  |  |  |  |
| 0 | Vvi-Vitvi07g01993\_t001 |  |  |  |  |  |  |  |  |  |  |  |  |
| 0 | Vvi-Vitvi07g04765\_t001 |  |  |  |  |  |  |  |  |  |  |  |  |
| 0 | Vvi-Vitvi07g01994\_t001 |  |  |  |  |  |  |  |  |  |  |  |  |
| 0 | Vvi-Vitvi07g04766\_t001 |  |  |  |  |  |  |  |  |  |  |  |  |
| 0 | Vvi-Vitvi07g04767\_t001 |  |  |  |  |  |  |  |  |  |  |  |  |
| 0 | Vvi-Vitvi07g01995\_t001 |  |  |  |  |  |  |  |  |  |  |  |  |
| 0 | Vvi-Vitvi07g01996\_t004 |  |  |  |  |  |  |  |  |  |  |  |  |
| 0 | Vvi-Vitvi07g04768\_t001 |  |  |  |  |  |  |  |  |  |  |  |  |
| 0 | Vvi-Vitvi07g01998\_t001 |  |  |  |  |  |  |  |  |  |  |  |  |
| 0 | Vvi-Vitvi07g01999\_t001 |  |  |  |  |  |  |  |  |  |  |  |  |
| 0 | Vvi-Vitvi07g02000\_t001 |  |  |  |  |  |  |  |  |  |  |  |  |
| 0 | Vvi-Vitvi07g02001\_t001 |  |  |  |  |  |  |  |  |  |  |  |  |
| 0 | Vvi-Vitvi07g04769\_t001 |  |  |  |  |  |  |  |  |  |  |  |  |
| 0 | Vvi-Vitvi07g02003\_t001 |  |  |  |  |  |  |  |  |  |  |  |  |
| 0 | Vvi-Vitvi07g02005\_t001 |  |  |  |  |  |  |  |  |  |  |  |  |
| 0 | Vvi-Vitvi07g02006\_t001 |  |  |  |  |  |  |  |  |  |  |  |  |
| 0 | Vvi-Vitvi07g04770\_t001 |  |  |  |  |  |  |  |  |  |  |  |  |
| 0 | Vvi-Vitvi07g02720\_t001 |  |  |  |  |  |  |  |  |  |  |  |  |
| 0 | Vvi-Vitvi07g02007\_t002 |  |  |  |  |  |  |  |  |  |  |  |  |
| 0 | Vvi-Vitvi07g02009\_t001 |  |  |  |  |  |  |  |  |  |  |  |  |
| 0 | Vvi-Vitvi07g02010\_t001 |  |  |  |  |  |  |  |  |  |  |  |  |
| 0 | Vvi-Vitvi07g02011\_t001 |  |  |  |  |  |  |  |  |  |  |  |  |
| 0 | Vvi-Vitvi07g02012\_t001 |  |  |  |  |  |  |  |  |  |  |  |  |
| 0 | Vvi-Vitvi07g02013\_t001 |  |  |  |  |  |  |  |  |  |  |  |  |
| 0 | Vvi-Vitvi07g02014\_t001 |  |  |  |  |  |  |  |  |  |  |  |  |
| 0 | Vvi-Vitvi07g02015\_t001 |  |  |  |  |  |  |  |  |  |  |  |  |
| 0 | Vvi-Vitvi07g02721\_t001 |  |  |  |  |  |  |  |  |  |  |  |  |
| 0 | Vvi-Vitvi07g02722\_t001 |  |  |  |  |  |  |  |  |  |  |  |  |
| 0 | Vvi-Vitvi07g02016\_t001 |  |  |  |  |  |  |  |  |  |  |  |  |
| 0 | Vvi-Vitvi07g02019\_t001 |  |  |  |  |  |  |  |  |  |  |  |  |
| 0 | Vvi-Vitvi07g02020\_t001 |  |  |  |  |  |  |  |  |  |  |  |  |
| 0 | Vvi-Vitvi07g02021\_t001 |  |  |  |  |  |  |  |  |  |  |  |  |
| 0 | Vvi-Vitvi07g02022\_t001 |  |  |  |  |  |  |  |  |  |  |  |  |
| 0 | Vvi-Vitvi07g02723\_t001 |  |  |  |  |  |  |  |  |  |  |  |  |
| 1 | Vvi-Vitvi07g02023\_t001 |  | Atr-ERM99739 |  |  |  |  |  |  |  |  |  |  |
| 1 | Vvi-Vitvi07g04771\_t001 |  | \| \| |  |  |  |  |  |  |  |  |  |  |
| 1 | Vvi-Vitvi07g04772\_t001 |  | Atr-ERM99743 |  |  |  |  |  |  |  |  |  |  |
| 1 | Vvi-Vitvi07g02028\_t001 |  | Atr-ERM99751 |  |  |  |  |  |  |  |  |  |  |
| 1 | Vvi-Vitvi07g02029\_t001 |  | Atr-ERM99771 |  |  |  |  |  |  |  |  |  |  |
| 1 | Vvi-Vitvi07g02031\_t001 |  | Atr-ERM99772 |  |  |  |  |  |  |  |  |  |  |
| 1 | Vvi-Vitvi07g02032\_t001 |  | Atr-ERM99777 |  |  |  |  |  |  |  |  |  |  |
| 0 | Vvi-Vitvi07g02034\_t001 |  |  |  |  |  |  |  |  |  |  |  |  |
| 0 | Vvi-Vitvi07g04773\_t001 |  |  |  |  |  |  |  |  |  |  |  |  |
| 0 | Vvi-Vitvi07g02035\_t001 |  |  |  |  |  |  |  |  |  |  |  |  |
| 0 | Vvi-Vitvi07g02036\_t001 |  |  |  |  |  |  |  |  |  |  |  |  |
| 0 | Vvi-Vitvi07g02038\_t001 |  |  |  |  |  |  |  |  |  |  |  |  |
| 0 | Vvi-Vitvi07g02039\_t001 |  |  |  |  |  |  |  |  |  |  |  |  |
| 0 | Vvi-Vitvi07g02724\_t001 |  |  |  |  |  |  |  |  |  |  |  |  |
| 0 | Vvi-Vitvi07g02040\_t001 |  |  |  |  |  |  |  |  |  |  |  |  |
| 0 | Vvi-Vitvi07g02041\_t001 |  |  |  |  |  |  |  |  |  |  |  |  |
| 0 | Vvi-Vitvi07g02042\_t001 |  |  |  |  |  |  |  |  |  |  |  |  |
| 0 | Vvi-Vitvi07g02043\_t001 |  |  |  |  |  |  |  |  |  |  |  |  |
| 0 | Vvi-Vitvi07g04774\_t001 |  |  |  |  |  |  |  |  |  |  |  |  |
| 0 | Vvi-Vitvi07g02045\_t001 |  |  |  |  |  |  |  |  |  |  |  |  |
| 0 | Vvi-Vitvi07g04775\_t001 |  |  |  |  |  |  |  |  |  |  |  |  |
| 0 | Vvi-Vitvi07g02047\_t001 |  |  |  |  |  |  |  |  |  |  |  |  |
| 0 | Vvi-Vitvi07g02048\_t001 |  |  |  |  |  |  |  |  |  |  |  |  |
| 0 | Vvi-Vitvi07g02049\_t001 |  |  |  |  |  |  |  |  |  |  |  |  |
| 0 | Vvi-Vitvi07g02050\_t001 |  |  |  |  |  |  |  |  |  |  |  |  |
| 0 | Vvi-Vitvi07g02053\_t001 |  |  |  |  |  |  |  |  |  |  |  |  |
| 0 | Vvi-Vitvi07g02055\_t001 |  |  |  |  |  |  |  |  |  |  |  |  |
